# Implications of Targeted Genomic Disruption of β-Catenin in BxPC-3 Pancreatic Adenocarcinoma Cells

**DOI:** 10.1371/journal.pone.0115496

**Published:** 2014-12-23

**Authors:** Petter Angell Olsen, Nina Therese Solberg, Kaja Lund, Tore Vehus, Monika Gelazauskaite, Steven Ray Wilson, Stefan Krauss

**Affiliations:** 1 Unit for Cell Signaling, Cancer Stem Cell Innovation Centre (SFI-CAST), Oslo University Hospital-Rikshospitalet, Oslo, Norway; 2 Department of Chemistry, University of Oslo, Oslo, Norway; Northwestern University Feinberg School of Medicine, United States of America

## Abstract

Pancreatic adenocarcinoma (PA) is among the most aggressive human tumors with an overall 5-year survival rate of <5% and available treatments are only minimal effective. WNT/β-catenin signaling has been identified as one of 12 core signaling pathways that are commonly mutated in PA. To obtain more insight into the role of WNT/β-catenin signaling in PA we established human PA cell lines that are deficient of the central canonical WNT signaling protein β-catenin by using zinc-finger nuclease (ZFN) mediated targeted genomic disruption in the β-catenin gene (*CTNNB1*). Five individual *CTNNB1* gene disrupted clones (BxPC3ΔCTNNB1) were established from a BxPC-3 founder cell line. Despite the complete absence of β-catenin, all clones displayed normal cell cycle distribution profiles, overall normal morphology and no elevated levels of apoptosis although increased doubling times were observed in three of the five BxPC3ΔCTNNB1 clones. This confirms that WNT/β-catenin signaling is not mandatory for long term cell growth and survival in BxPC-3 cells. Despite a normal morphology of the β-catenin deficient cell lines, quantitative proteomic analysis combined with pathway analysis showed a significant down regulation of proteins implied in cell adhesion combined with an up-regulation of plakoglobin. Treatment of BxPC3ΔCTNNB1 cell lines with siRNA for plakoglobin induced morphological changes compatible with a deficiency in the formation of functional cell to cell contacts. In addition, a re-localization of E-cadherin from membranous in untreated to accumulation in cytoplasmatic puncta in plakoglobin siRNA treated BxPC3ΔCTNNB1 cells was observed. In conclusion we describe in β-catenin deficient BxPC-3 cells a rescue function for plakoglobin on cell to cell contacts and maintaining the localization of E-cadherin at the cellular surface, but not on canonical WNT signaling as measured by TFC/LEF mediated transcription.

## Introduction

Pancreatic adenocarcinoma (PA) is the most common type of cancers in the pancreas and is the fourth leading cause of cancer deaths in developed countries [1]. PA is an aggressive cancer type where available treatments are only minimal effective. The expected 5 year survival rate is less than 5%, a statistic that has remained largely unchanged the past 40 years [2]. Given that human cancers primarily are genetic diseases, characterization of the genetic changes present in the cancer and validating their impact on cancer progression is important for developing better treatment and prevention strategies. For advanced pancreatic adenocarcinoma, global genomic analysis has shown an average of 63 genetic alterations in 12 key cellular signaling pathways [3]. Although there are genes that are found to be mutated in the majority of PAs (*KRAS*, *CDKN2A*, *TP53* and *SMAD4*), PA is a highly heterogenetic disease [3] and type and number of mutations varies considerably even within the same tumor [4].

WNT/β-catenin signaling is one of the pathways that were identified as a core signaling pathway that is altered in most pancreatic cancers [3]. In healthy cells tightly controlled WNT/β-catenin signaling regulates processes such as cell proliferation, energy metabolism, cell migration and asymmetric cell division and thus has central roles in embryonic development and maintenance of tissue homeostasis [5]. In general, activation of the WNT signaling pathway is initiated by binding of secreted WNT proteins to their receptor complexes which leads, context dependent, to triggering of intracellular signaling transduction cascades. These cascades are frequently subdivided in three branches: non canonical WNT/calcium signaling, non canonical planar cell polarity pathway (PCP) and canonical WNT signaling. In non-canonical WNT signaling the pathway is independent of β-catenin while in canonical WNT signaling the regulation of WNT target genes is mediated by the β-catenin protein. Activation of canonical WNT signaling involves stabilization and translocation of β-catenin from the cytoplasm to the nucleus where it binds predominantly to transcription factors of the TCF/LEF family to activate transcription of WNT target genes [6]. Besides its role as a transcriptional regulator β-catenin is also involved in cell adhesion. A significant pool of β-catenin is located at adherens junctions where β-catenin interacts with the cytoplasmatic domain of E-cadherin. Binding of β-catenin along with α-catenin to E-cadherin links adherens junctions with the actin cytoskeleton. The association of β-catenin to E-cadherin has been shown to prevent proteosomal degradation of both E-cadherin and β-catenin [7].

Although the WNT/β-catenin signaling pathway has been identified as one of the key pathways commonly mutated in PA, the complex role of β-catenin mediated signaling in PA has been unclear. In mouse models constitutive activation of WNT signaling is unable to initiate PA [8] and somatic mutations of key intracellular WNT regulatory molecules such as *APC*, *AXIN1* and *CTNNB1* are rare in human PA [3].

In this study we investigated the consequence of a complete β-catenin depletion in PA by using zinc-finger nucleases (ZFNs) to generate cell lines in which β-catenin is absent due to targeted genomic disruption of the β-catenin gene (*CTNNB1*). While siRNA knockdown will reduce the protein load of a cell, leaving room for residual biological activity of a targeted protein, a targeted knockout allows eliminating the biological impact of residual amounts of a protein.

Of the three PA cell lines, BxPC-3, PANC-1 and PANC-03.27, that were subjected to ZFN mediated *CTNNB1* targeting, β-catenin deficient cells could only be derived from BxPC-3 cells. BxPC-3 is a cell line that shows very low levels of WNT activity in an un-stimulated state as measured by a STF pathway reporter [9].

The β-catenin deficient BxPC-3 clones did not display altered morphology or increased levels of apoptosis and the cell cycle distribution was similar to wild type cells; nevertheless three of the clones showed reduced proliferation rates. A common feature of the β-catenin deficient clones was increased protein levels of plakoglobin (γ-catenin). Plakoglobin localizes at the cell membranes where it interacts with E-cadherin in a similar way as β-catenin, thus indicating a functional substitution for β-catenin at the adherens junctions. Only when in addition to a β-catenin knockout, also levels of plakoglobin were reduced by small interfering RNA (siRNA), cells changed their shape and displayed a rounded morphology with an apparent disability to form normal cell to cell connections. Analysis of core adherens junction proteins in the β-catenin and plakoglobin deficient cells revealed a significant reduction of α-catenin and p120-catenin. In addition, the localization of E-cadherin in the double β-catenin and plakoglobin deficient cells was changed from being predominantly membranous to being localized in intracellular puncta. The data from the β-catenin deficient BxPC-3 PA cells points towards a central role of β-catenin in enabling cell-cell contacts.

## Materials and Methods

### Cell lines

The human pancreatic adenocarcinoma BxPC-3 (ATCC CRL-1687) epithelial cell line was grown in RPMI-1640 (Sigma-Aldrich, St Louis, MO, USA) supplemented with 10% fetal bovine serum, 1% penicillin/Streptomycin and 0.002 x Insulin-Transferrin-Selenium (Life Technologies, Carlsbad, CA, USA). PANC-03.27 (ATCC CRL-1469) was grown in the same media as BxPC-3 cells apart from being supplemented with 0.1 x Insulin-Transferrin-Selenium (Life Technologies). PANC-1 (ATCC, CRL-2549) were grown in DMEM supplemented with 10% fetal bovine serum and 1% penicillin/Streptomycin. All cells were propagated at 37°C in a humidified atmosphere containing 5% CO_2_.

### Generation of β-catenin deficient cells using CompoZr custom ZFNs

**Figure 1:**
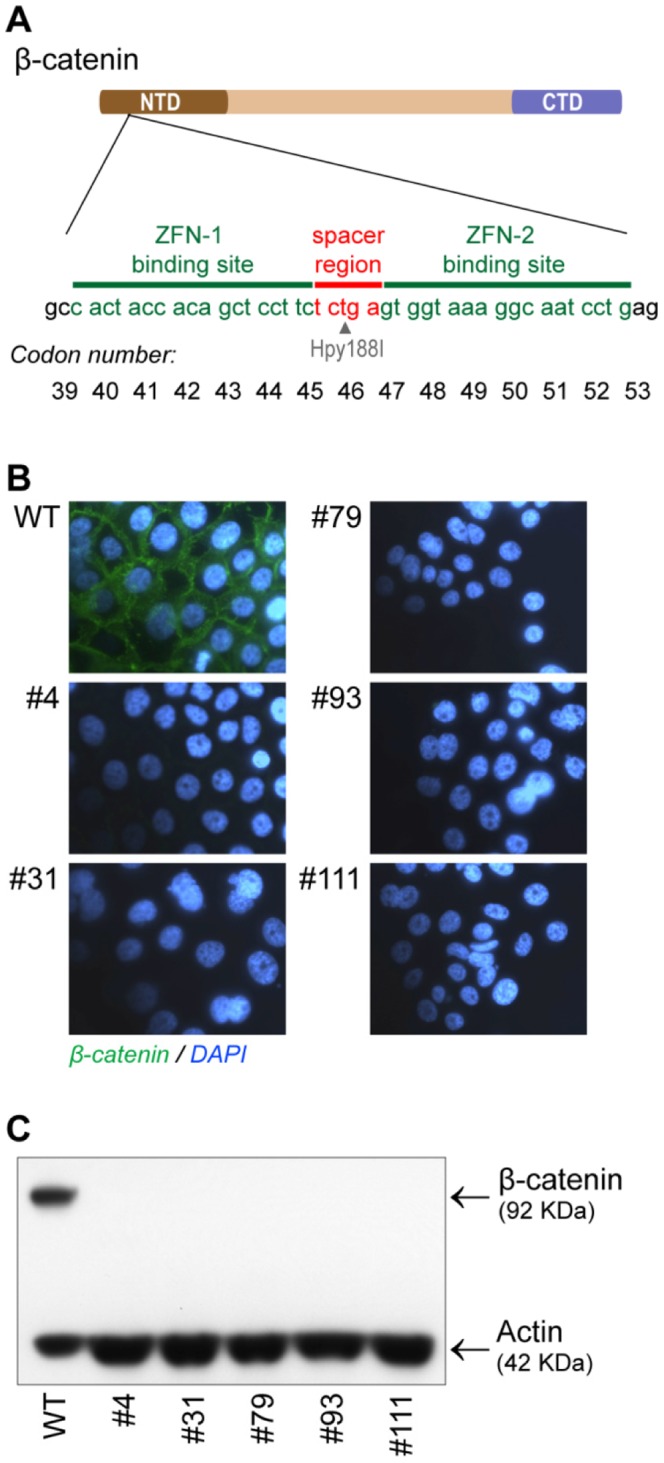
Genomic disruption of the β-catenin gene (*CTNNB1*) in BxPC-3 cells using ZFNs. A) Outline of the *CTNNB1* gene with the N-terminal (NTD) and C-terminal (CTD) domains colored in brown and blue, respectively. Binding sequence for ZFN-1 and -2 (green) flanking the spacer region (red) incorporating the recognition site for the Hpy188I restriction endonuclease are shown with the corresponding codon number in the *CTNNB1* gene below. B) β-catenin immunostaining of BxPC-3 wild type (WT) and *CTNNB1* gene disrupted clones (#4, #31, #79, #93 and #111) with DAPI nuclear counterstaining. In the wild type cells β-catenin is predominantly localized at the cell membranes. C) Western blot analysis of WT and gene disrupted clones using β-catenin (CTD) and actin specific Abs. For wild type cells 5 µg of total protein (TP) and for the gene disrupted clones 20 µg of TP was applied for each lane. The position and molecular weight of the β-catenin and actin bands are indicated.

BxPC-3 cells with targeted disruption of the β-catenin gene (*CTNNB1*) were established using CompoZr custom Zinc Finger Nucleases (ZFNs) (Sigma-Aldrich). In this technology, distinct ZFNs are engineered to introduce site specific genomic double strand breaks (DSBs) that would stimulate recombination processes resulting in introduction of small genomic changes at the target site [10]. The ZFNs used in this study each consisted of a 6 zinc finger DNA binding domain fused to obligate heterodimer FokI cleavage domains designed to introduce specific DSBs in exon 3 of the human *CTNNB1* gene (Fig. 1A). To achieve high transfection efficiencies and transient expression patterns, BxPC-3 cells were transfected with mRNA encoding the ZFNs. Capped and Poly(A) tailed ZFN mRNA was produced using the mMESSAGE mMACHINE T7 Ultra Kit (Life Technologies) and purified using the MEGAclear kit (Life Technologies) according to the manufactures instructions. Amaxa nucleofector kit L was used for introducing the ZFNs mRNA into the cells. Briefly, 2.0×10^6^ cells were mixed with 2.0 µg of each ZFNs mRNA and nucleofected using program V-001. Immediately after nucleofection the cells were transferred to 10 cm dishes and incubated at 30°C for 72 hours before returning to 37°C for 24 hours. Incubating cells at for a period at lowered temperatures has previously been shown to increase the frequency of ZFNs mediated genome modifications [11]. Next, monoclonal cell populations were obtained by limiting dilution cloning and analyzed for β-catenin expression by immunostaining (see below). From 150 initial clones 5 clones negative for β-catenin immunostaining were identified and selected for further analysis (clone #4, #31, #79, #93 and #111).

### Immunostaining of cells

For immunostaining cells were fixed (4% paraformaldehyde) for 10 minutes at room temperature (RT) and blocked (5% bovine serum albumin (BSA), 5% goat serum, 0.1% Tween-20 in phosphate buffered saline (PBS)) for 1 hour at RT followed by incubation with primary antibody (Ab) (0.5% BSA, 0.5% goat serum, 0.1% Tween-20 in PBS) at 4°C over night. After washing in PBS cells were incubated with fluorescently labeled secondary Ab (0.5% BSA, 0.5% goat serum, 0.1% Tween-20 in PBS) for 30 minutes at RT. For nuclear counterstaining the cells were incubated with DAPI (1 µg/ml) for 10 minutes at RT. Cells stained on cover slips were mounted on slides using Fluorescence Mounting Medium (DAKO, Denmark). The following primary Abs and dilutions were used: β-catenin CTD (BD Transduction Laboratories, BD Biosciences, CA, USA, #610153, 1∶2000), γ-catenin (BD Transduction Laboratories, #610253, 1∶2000) β-catenin NTD (Abcam, Cambridge, UK, ab32572, 1∶250). Secondary Abs used were: Alexa Fluor 488 Donkey Anti-Mouse, (Life Technologies, A-21202, 1∶500), Alexa Fluor 488 Donkey Anti-Rabbit (Life Technologies, A-21206, 1∶500). Images were visualized under a fluorescence microscope (Zeiss Axiovert 200 M, Carl Zeiss, Germany) and acquired with CCD camera (Zeiss Axiocam HR).

### Western blot analysis

Cells were lysed in RIPA buffer containing protease inhibitor and phosphatase inhibitor tablets (Roche, IN, USA). Cell lysates were cleared by centrifugation and protein concentration determined by Bio-Rad Protein Assay kit (Bio-Rad Laboratories, CA, USA). 5–30 µg (as indicated in the figure legends) of total protein in SDS sample buffer was loaded per lane and separated on NuPAGE Tris-Acetate precast polyacrylamide gels (Life Technologies). Detection of proteins was done with the following primary Abs and dilutions: β-catenin CTD (BD Transduction Laboratories, #610153, 1∶2000), γ-catenin (BD Transduction Laboratories, #610253, 1∶5000) β-catenin NTD (Abcam, ab32572, 1∶5000), E-cadherin (BD Transduction Laboratories, #610181, 1∶4000), α-Catenin (BD Transduction Laboratories, #610193, 1∶500), p120-catenin (BD Transduction Laboratories, #610133, 1∶2000), Actin (Sigma-Aldrich, A2066, 1∶2000). Secondary Abs were: donkey anti-mouse IgG-HRP (Santa Cruz Biotechnology, CA, USA, sc-2314, 1∶5000), donkey anti-rabbit IgG-HRP (Santa Cruz biotechnology, sc-2313, 1∶5000). Signals were developed using ECL Prime Western Blotting Detection Reagent (GE Healthcare, Buckinghamshire, UK) and quantification of signal intensities was done using the Image Studio Lite software (LI-COR, NB, USA).

### PCR genotyping and genomic sequencing

Genomic DNA was isolated using GenElute Mammalian Genomic DNA Purification Kit (Sigma-Aldrich) and PCR amplification of *CTNNB1* exon 3 was performed by standard PCR using Phusion DNA polymerase (New England Biolabs, MA, USA) with primers Ex3-F (5′-caa tgg gtc ata tca cag att ctt-3′) and Ex3-R (5′-tca aaa ctg cat tct gac ttt ca-3′). For RFLP analysis the PCR products from *CTNNB1* exon 3 were purified (illustra GFX PCR DNA and Gel Band Purification Kit, GE Healthcare) and digested with Hpy188I restriction enzyme (New England Biolabs) before separation on 4% agarose gel (Agarose 1000, Invitrogen, CA, USA).

For sequencing, PCR products form *CTNNB1* exon 3 were TOPO cloned (Zero Blunt TOPO PCR Cloning Kit, Life Technologies) and plasmid DNA was isolated (illustra plasmidPrep Mini Spin Kit, GE Healthcare) and sent for sequencing (GATC Biotech, Germany) using the M13 forward primer (5′- tgt aaa acg acg gcc agt-3′). For each of the β-catenin deficient cell clones (#4, #31, #79 and #93), plasmid DNA from 10 individual bacterial colonies were sequenced.

**Figure 2:**
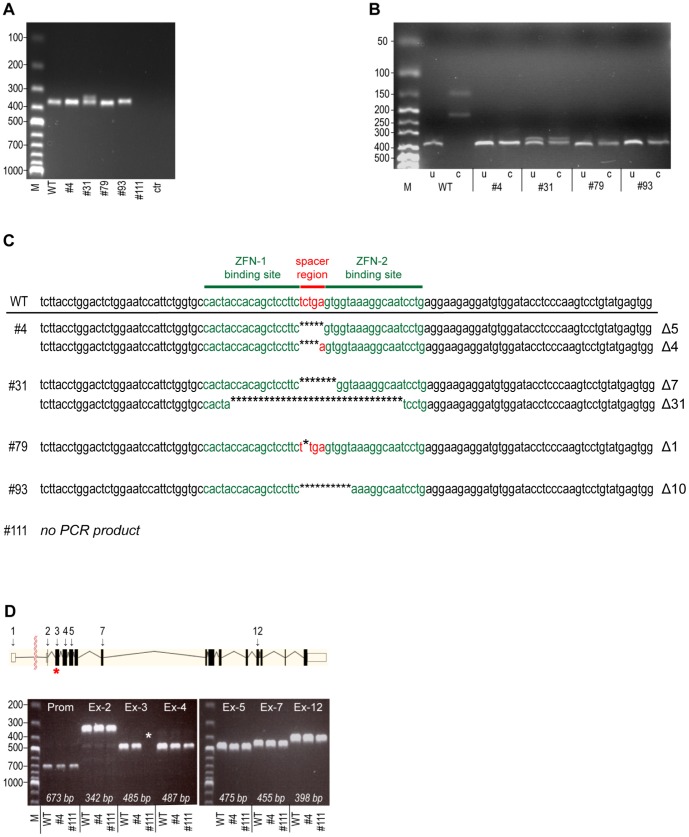
Characterization of genomic changes present in the *CTNNB1* gene disrupted cells. A) PCR analysis of the integrity of exon 3 in the *CTNNB1* gene using genomic DNA isolated from WT and gene disrupted clones (#4, #31, #79, #93 and #111) as template. ctr: negative control devoid of gDNA, M: molecular size marker. B) Genotyping of WT and gene disrupted clones by RFLP. Digestion of PCR products spanning exon 3 of the *CTNNB1* gene with the Hpy188I restriction endonuclease that would cut undisrupted wild type sequences. u: PCR product without Hpy188I, c: PCR product with Hpy188I, M: molecular size marker. C) Sequencing of PCR products spanning exon 3 of *CTNNB1* gene from WT and gene disrupted clones. The binding sequence for ZFN-1 and -2 (green) flanking the spacer region (red) is indicated above. * in the sequence indicates deleted base and the total number of deleted bases are shown at the right. D) PCR analysis of exon integrity in WT and gene disrupted clones #4 and #111 using primers designed for amplifying the promoter region (Prom) and exons 2, 3, 4, 5, 7 and 12 (Ex-2, 3, 4, 5, 7 and 12 respectively). Expected sizes of amplification products of intact sequences are indicated. M: molecular size marker. Upper panel: Schematic representation of the *CTNNB1* gene structure.

For investigation of the *CTNNB1* exon integrity in the β-catenin deficient clone #111 (Fig. 2D) PCR was performed (Phusion DNA polymerase) on genomic DNA with the following primer pairs (5′-3′): Prom-F/R (tga att gtg acc aca acc aat/ttg tgg gga ttt ttc ttt gg), Ex2-F/R (gat gga gct gtg gtt gag gt/aag cag gga gag agg aaa gc), Ex3-F/R (ccc tgg cta tca ttc tgc tt/tct ctt ttc ttc acc aca aca ttt), Ex4-F/R (gca atg tca ctt tta cca ttt agg/tgc agc ctt att aac cac ca), Ex5-F/R (gac gag gac cag gta agc aa/cca ctg gtg aac tgg gaa ga), Ex7-F/R (gtt gga tag ggc ccc agt at/tgg ctg caa act gaa tag ga), Ex12-F/R (tgt gaa tgc ctc ttg cac tc/tct tgt ggc ttg tcc tca ga).

### Quantitative real time PCR (qRT-PCR)

Total mRNA was isolated (GenElute Mammalian Total RNA Purification Kit, Sigma-Aldrich) following the manufacturer's instructions. cDNA was synthesized using the SuperScript VILO kit (Life Technologies), and qRT-PCR was carried out using TaqMan gene expression master mix (Life Technologies) according to the manufacturer's instructions on a StepOnePlus cycler (Life Technologies). GAPDH was used to normalize the amount of cDNA in each sample and to guarantee the comparability of the calculated mRNA expression in all samples analyzed. The following TaqMan probes were used: GAPDH (Hs02758991_g1), CTNNB1 (Hs00355049_m1), JUP (plakoglobin) (Hs00158408_m1) all from Life Technologies.

### Cell cycle analysis

Single cell suspensions were fixed overnight at −20°C in 70% EtOH before resuspension in propidium iodide (PI) staining solution (20 µg/ml PI, 200 µg/ml RNaseA, 0.1% Triton X-100) for 30 minutes at RT. Flow cytometric analysis was performed on a FacsARIA (BD Biosciences) where the pulse width versus pulse area signal was used to exclude doublets. For each sample 50 000 cells were acquired.

### Live cell proliferation assay

For measurement of cell proliferation the IncuCyte FLR live cell imaging system (Essen BioScience, MI, USA) was used to quantify the confluency of living cells. Calculation of the doubling times (Td) was done using the online calculator at http://www.doubling-time.com/compute.php (equation used: amount  = 0.5288×e^0.0317×time^).

### Supertop flash luciferase reporter gene assay

The day before transfection 200 000 cells were plated out in 12 well dishes. For each transfection, 1.8 µg of the TCF/LEF reporter pSuperTopFlash (8xTCF/LEF binding sites driving Firefly luciferase expression) [12] or 1.8 µg of the control pSuperFopFlash (mutated TCF/LEF binding sites) [12] were mixed with 0.2 µg of the internal control construct pRL-TK (constitutive Renilla luciferace expression) (Promega, WI, USA) and the transfection was done using 2.2 ul Lipofectamine 2000 ((LF2000), Life Techonologies) for wild type, clone #4 and clone #31 cells or 2.2 ul Lipofectamine 2000 for clone #79, clone #93 and clone #111 cells in growth medium without antibiotics. 24 hours after transfection the media was replaced with growth medium with or without 20 mM LiCl_2_ (Sigma-Aldrich). 48 hours after transfection the cells were harvested in passive lysis buffer and both Firefly and Renilla luciferase activity was measured in the same sample using the Dual-Luciferase Reporter Assay System (Promega). The luminescence was quantified on a GloMax-Multi microplate reader (Promega) according to the manufactures instructions.

### Quantitative proteonomics by SILAC

Detailed description of the SILAC analysis is included in the S1 Materials and Methods. Briefly, following growth and labeling of BxPC-3 wild type and *CTNNB1* gene disrupted clones (#4, #31, #79, #93 and #111) in Light and Heavy media, respectively the cells were harvested and lysates from each of the *CTNNB1* gene disrupted clones were combined in a 1∶1 ratio (protein concentration) with lysates from wild type cells. After enzymatic digestion the samples were subjected to liquid chromatography-tandem mass spectrometry and isotopic peptide pairs were quantified with proteome discoverer (Thermo Fisher Scientific, MA, USA). Perseus (www.maxquant.org) was used to generate the heat map and Ontology enrichment analysis was done using DAVID [13] with the GOTERM_BP_2 annotation. A complete list of all proteins detected and quantified in the individual β-catenin deficient clones is included in S5 Table.

### Microarray analysis

Total RNA (GenElute Mammalian Total RNA Purification Kit, Sigma-Aldrich) was isolated from wild type BxPC-3 cells and *CTNNB1* gene disrupted clones #4 and #111. The RNA was subjected to microarray analysis using Illumina HumanHT-12 v4 Expression BeadChips (Illumina, CA, USA) at the Norwegian Genomics Consortium core facility (Oslo University Hospital, Norway). For each sample 6 biological replicates were analyzed. Data extraction and quality control was performed in GenomeStudio (Illumina) and the data analysis was performed using J-Express [14]. Ontology enrichment analysis was done using DAVID [13] with the GOTERM_BP_2 annotation. The results from the microarray analysis have been deposited to the Gene Expression Omnibus repository with accession number GSE63072.

### AnnexinV apoptosis assay

Cells (1×10^5^–1×10^6^) were resuspended in 500 µl Annexin V binding buffer before addition of 1 µl of 5xAnnexin V-FITC apoptosis detection reagent (Abcam, Cambridge, UK). After 5 minutes incubation at RT the number of FITC positive cells were quantified by flow cytometry (Partec PAS, Partec GmbH, Germany).

### Co-immunoprecipitation

Cultured cells were trypsinized and lysed for 30 min on ice in cold non-denaturing lysis buffer (50 mM Tris pH 8.0, 150 mM NaCl, 1% NP40, 2 mM EDTA) containing protease inhibitor cocktail (cOmplete Tablets, Roche). Lysates were spun for 15 min at 13 000 rpm to remove cell debris, and protein concentrations were measured using Quick Start Bradford 1x Dye Reagent (Bio-Rad Laboratories) according to the manufacturer's instructions. 50 µl Dynabeads Protein G magnetic beads (Invitrogen) was used according to the manufacturer's instructions. 5 µg anti-rabbit plakoglobin (γ-catenin) antibody (ab15153, Abcam) was bound to pre-washed beads in 100 µl MQ H_2_O for one hour on a spinning wheel at RT, with a parallel containing 5 µg normal rabbit IgG (sc2027, Santa Cruz Biotechnology) as negative control. Dynabeads/antibody complex was incubated with 400 µg protein on a rotation wheel at 4°C over night. Immunoprecipitated proteins were eluted for 5 min at RT on a spinning wheel with 2×40 µl elution buffer (0.1 M citric acid). 20 µl 5x loading buffer (LB) for SDS-PAGE was added to the eluates, and 30 µl was further resolved by NuPAGE Novex 3–8% Tris-Acetate (Life Technologies) together with 10 µg input (positive control), immunoblotted and probed with primary antibodies against mouse γ-catenin (BD Transduction Laboratories #610253, 1∶5000) and mouse E-Cadherin (BD Transduction Laboratories #610181, 1∶4000). Primary antibodies were visualized with HRP-conjugated antibodies (Santa Cruz Biotechnology) (1∶10000) and chemiluminescent substrate ECL Prime Western Blotting Detection Reagent (GE Healthcare).

### siRNA mediated gene knockdown

The day before transfection 200 000 cells were plated out in 6 well plates. Transfection were done with 40 nM of either MISSION siRNA Universal Negative Control #1 with no homology to all mature and predicted RefSeq mRNA sequences (SIC001, Sigma-Aldrich) or MISSION plakoglobin siRNA (ID: SASI_Hs01_00246520, Sigma-Aldrich) using 5 µl of LF2000 in a total of 2.5 ml growth medium without antibiotics. After 24 hours the medium was replaced and the cells were incubated for another 48 hours before analysis/harvesting.

### Statistical analysis and replicates

**Figure 3:**
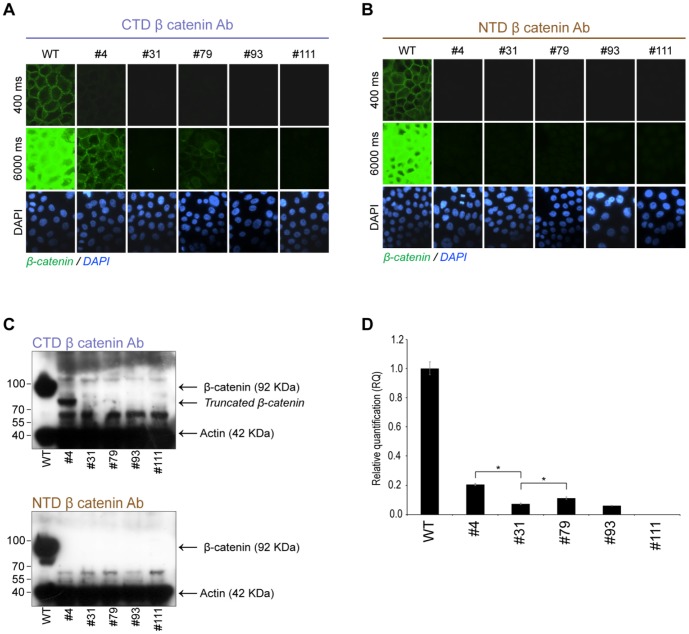
Detection of truncated β-catenin protein in the *CTNNB1* gene disrupted clones #4 and #79. A) Immunostaining of WT and gene disrupted clones (#4, #31, #79, #93 and #111) with a CTD specific anti β-catenin Ab (green). Images were acquired using both normal (400 milliseconds (ms)) or extended (6000 ms) exposure times as indicated. Nuclear counterstaining with DAPI is shown at the bottom row. B) Immunostaining of WT and gene disrupted clones with a NTD specific anti β-catenin Ab (green) and acquired as in A. C) WB analysis of total cell extracts from WT and gene disrupted cells using CTD (upper panel) or NTD (lower panel) specific anti β-catenin Abs together with anti actin Ab. The position and full length β-catenin, truncated β-catenin and actin bands are indicated. For wild type cells 5 µg of TP and for the gene disrupted clones 30 µg of TP was applied for each lane. D) Relative quantification of β-catenin mRNA levels using qRT-PCR in wild type and gene disrupted clones. Error bars represent SD of the mean relative quantification (RQ) value and * indicates P<0.002 between the compared bars.

Error bars in figures indicates standard deviation (SD) of the mean from three measurements in an experiment. Student t-test (unpaired) was performed to access statistical significance between the mean CTNNB1 relative quantification (RQ) value of three parallel measurement from BxPC3ΔCTNNB1 clone #4 versus clone #31 and clone #79 versus clone #31 as indicated in Fig. 3D. In the Ontology analysis significantly enriched terms were identified and sorted on the basis on the Bonferroni adjusted p-value provided by DAVID [13]. Significant differentially expressed probes from the microarray experiments were identified and sorted based on fold change, q-value and d-score obtained from “Significance analysis of microarrays” using the J-Express software [14]. The WB, Immunostaining, qRT-PCR, cell cycle, luciferase, apoptosis, co-immunoprecipitation and siRNA knockdown experiments were carried out at least three times on individually prepared samples (experimental replicates). In the figures results from representative experiments are shown.

## Results

### Generation of β-catenin deficient cells using ZFNs

Although functional studies based on the convenient knockdown strategy by siRNA can provide significant information on the function of a targeted protein, siRNA only reduces the amount of a given protein, leaving the possibility that the protein can maintain some of its core functions [15]. In contrast, disruption of both copies of the genomic sequence of a gene by targeted knockout strategies that are enabled by ZFNs, TALENs or CRISPR/Cas9, will lead to a complete and permanent loss of the targeted protein expression [16], [17].

To study the functional implications of a complete deletion of β-catenin in PA, several pancreas adenoma cell lines, including PANC-03.27, PANC-1 and BxPC-3 were targeted with ZFNs [10] designed to introduce genomic double strand breaks (DSBs) in exon 3 of the β-catenin gene (*CTNNB1*) (Fig. 1A). A knockout in a cancer cell line can be demanding since tumor cell lines may have abnormal numbers of chromosomes and large scale structural rearrangements of chromosomes [18], [19]. Cytogenetic analyses of PA cell lines have shown highly complex karyotypes and due to genetic drifting, there may be differences within the cell lines and between passages and batch numbers [19], [20]. In addition, if the activity of the targeted gene is essential for cell proliferation or cell survival, isolation and propagation of knockout clones would not be possible.

After transfection of the PA cell lines with mRNA of β-catenin exon 3 specific ZFNs (Fig. 1A), clones from individual cells were isolated by limiting dilution cloning and subsequently expanded and analyzed for β-catenin expression by immunostaining. Despite repeated attempts, in our hands, β-catenin negative clones could only be obtained from the BxPC-3 cell line. In this cell line, from the 150 individual ZFN treated clones initially analyzed, 5 clones negative for β-catenin immunostaining were identified and chosen for further experimental exploration (Fig. 1B, BxPC3ΔCTNNB1 clone #4, #31, #79, #93 and #111). In agreement with the immunohistochemistry results for these clones, Western blot analysis identified a band corresponding to the β-catenin protein (92 kDa) solely in lysates from the wild type cells and not in lysates from the isolated clones #4, #31, #79, #93 and #111 (Fig. 1C).

To characterize the genomic alterations in the β-catenin deficient cells PCR analysis on genomic DNA (gDNA) with primers spanning exon 3 of the *CTNNB1* gene (incorporating the ZFNs binding sites), was carried out (Fig. 2A). In clone #4, #79 and #93 a single amplification product with the expected size of undisrupted gDNA (364 bp) was obtained. Clone #31 displayed an extra shorter band in addition to the band with the expected size and in clone #111 no PCR product could be obtained. Thus it appeared that in clone #4, #31, #79 and #93 the ZFN treatment had provoked minor genomic modifications while in clone #111 a larger deletion or rearrangement of exon 3 had been produced. To further characterize the genetic changes that had been produced in the clones, restriction fragment length polymorphism (RFLP) genotyping was carried out. In the wild type sequence, the spacer region that is flanked by the ZFNs binding sites incorporates a recognition site for the restriction enzyme Hpy188I (TCN'GA) (Fig. 1A). To test if the wild type sequence was present in the genome of the β-catenin deficient clones, the PCR products from the gDNA was subjected to Hpy188I digestion (Fig. 2B). As expected, the PCR product from the wild type cells produced Hpy188I digestion products compatible with an undisrupted recognition site (14 bp+141 bp+209 bp). The PCR products from all the clones (#4, #31, #79 and #93) were not digested by Hpy188I, indicating that the Hpy188I recognition site had been disrupted on both alleles. To map the induced genomic changes accurately, sequencing analysis of the PCR products were done (Fig. 2C). In concurrence with the results from the Hpy188I digestion, all clones displayed small deletions in the spacer region that would lead to an interrupted Hpy188I recognition site. Clone #4 and #31 contained two sequence variants each whereby clone #4 incorporated both a 4 bp and a 5 bp deletion while clone #31 contained both a 7 bp and a larger 31 bp deletion. The presence of a 31 bp deletion in clone #31 was in agreement with the observed shorter additional amplification product in Fig. 2A. Sequencing of the PCR products from clone #79 and #93 revealed that these clones harbored only one sequence variant: clone #79 incorporated a single bp deletion while clone #93 enclosed a 10 bp deletion in the spacer region. In clone #111 no amplification product could be obtained when primers specific for exon 3 were used (Fig. 2A). To get a further insight in the genomic changes that had occurred in clone #111, PCR with primers specific for the promoter region and primers amplifying exon 2, 3, 4, 5, 7 and 12 was done. As seen in Fig. 2D, amplification products with the expected size for undisrupted gDNA were obtained with all primers except for the exon 3 specific primer pair. This indicates that in clone #111, exon 3 had been deleted or rearranged while the flanking exons were still intact.

In summary, by employing the ZFN technology, we have isolated 5 individual clones of BxPC-3 cells that contained targeted deletions of the *CTNNB1* gene in exon 3 resulting in a disruption of the β-catenin reading frame leading to a loss of protein expression.

### BxPC3ΔCTNNB1 clone #4 and #79 express low levels of a truncated form of β-catenin

When the knockout cells were stained with an anti β-catenin antibody (Ab) recognizing the C-terminal domain (CTD), no β-catenin staining could be observed in the isolated clones (#4, #31, #79, #93 and #111) using exposure settings that generated an optimal β-catenin signal in wild type cells (Fig. 3A, upper panel (400 ms exposure time)). However, when using a prolonged exposure time (6000 ms) that resulted in substantial overexposure in the wild type cells, clone #4 exhibited clear β-catenin staining while in clone #79 weak β-catenin staining could be seen (Fig. 3A, middle panel). Given that the gDNA of both clone #4 and #79 incorporated deletions in exon 3 of *CTNNB1* gene that would disrupt the reading frame (Fig. 2B, 2C), the detection of a β-catenin immunofluorescent signal in clone #4 and #79 could be caused by the presence of a truncated β-catenin protein lacking the N-terminal domain (NTD). To check if this was the case, immunofluorescent staining using an Ab recognizing the NTD of β-catenin was carried out. As seen in Fig. 3B, the NTD specific Ab detected distinct β-catenin staining in the wild type cells, however, neither of the clones displayed β-catenin staining regardless of the exposure times used. In addition, WB analysis confirmed the presence of a truncated β-catenin protein in clone #4 which could only be detected with the Ab specific for the CTD domain (Fig. 3C, upper panel) and not with the NTD specific β-catenin Ab (Fig. 3C, lower panel). In concurrence with the immunostaining, increased protein amounts and long exposure times were required to detect the truncated β-catenin protein in clone #4 in the WB analysis, underscoring the low amounts of the protein in the clone. The apparent molecular weight of the truncated protein is between 70 and 80 kDa. This would correspond to a translation initiation from the first ATG present in exon 4 (codon 88) of *CTNNB1* coding sequence that would generate a protein of 689 amino acids with a predicted size around 75 kDa. Interestingly, when the CTD Ab was used in the WB, all clones displayed a band around 60 kDa that was not present in wild type cells (Fig. 2C). This shorter band could in theory also be attributed to translational initiation alternative ATG codons further downstream in the *CTNNB1* coding sequence.

To examine β-catenin transcription levels, quantitative real time (qRT) PCR was carried out on the wild type and β-catenin deficient clones. Interestingly, in addition to a loss of β-catenin protein (Fig. 3C), the clones with disrupted *CTNNB1* gDNA also displayed reduced amounts of β-catenin mRNA levels (Fig. 3D). Clone #4 and #79 exhibited a 80% and 89% reduction while clone #31 and #93 exhibited a 93% and 96% reduction in β-catenin mRNA levels relative to what was observed in wild type cells. In clone #111 no β-catenin mRNA could be detected. Although, these results could indicate that β-catenin mRNA levels are influenced by β-catenin protein levels, the reduced levels of β-catenin mRNA in the gene disrupted clones are most probably due to RNA decay pathways that degrades mRNA that incorporates premature translation termination codons (PTCs) [21].

### TCF/LEF mediated transcription cannot be activated in BxPC-3 cells deficient of β-catenin

**Figure 4:**
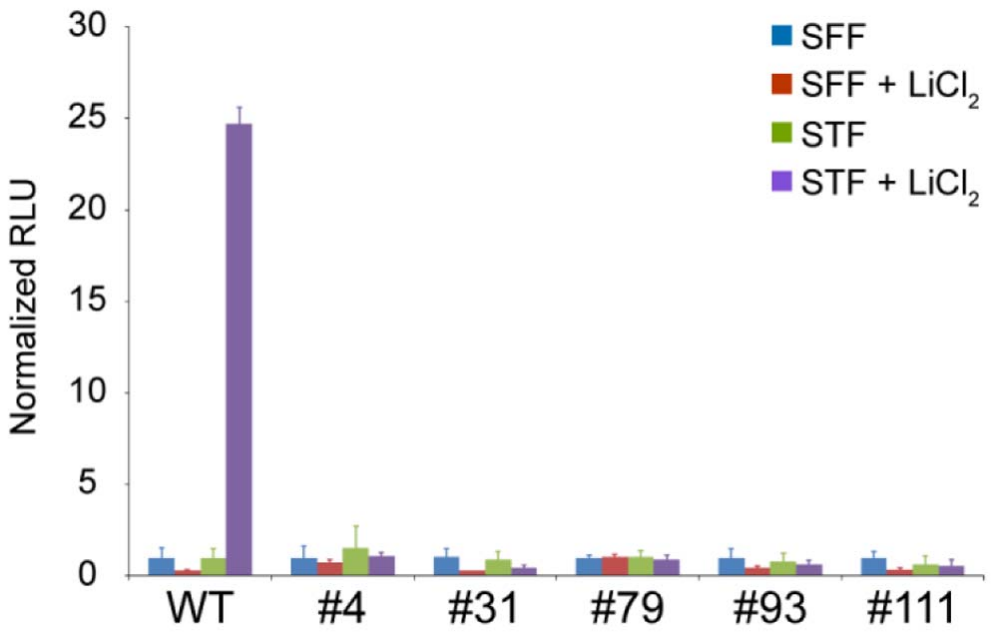
TCF/LEF mediated transcription cannot be activated in gene *CTNNB1* disrupted cells. Wild type and gene disrupted clones were transfected with the SFF control construct or the STF TCF/LEF responsive reporter gene plasmid and left untreated or treated with the GSK3β inhibitor, LiCl_2_ to induce WNT signaling mediated transcriptional activation. Three days after transfection lysates were harvested and luciferase reporter gene activity was quantified as Relative Light Units (RLU) as described in Materials and Methods. Error bars represent SD between three parallels.

One of the main functions of β-catenin is to mediate canonical WNT signaling through association with transcription factors predominantly of the TCF/LEF family to activate target gene expression [22]. To determine whether the absence of β-catenin completely abolished TCF/LEF mediated transcription, the BxPC-3 clones were transfected with the SuperTop Flash (STF) and SuperFop Flash (SFF) reporter gene constructs (containing wild type and mutated TCF/LEF binding sites respectively). Wild type cells that were transfected with the reporter constructs did not show measurable STF reporter activity in a native state (Fig. 4). However, when wild type cells were treated with the GSK3β inhibitor LiCl_2_ to block β-catenin degradation and thereby stimulate WNT signaling, a 25 fold increase in the STF reporter activity was observed (compared to the control SFF activity) demonstrating the presence of a viable WNT/β-catenin signaling pathway at the level of the destruction complex and downstream in BxPC-3 cells. In contrast, all β-catenin deficient clones neither showed a STF basal activity nor the ability to induce STF activity upon GSK3β inhibition, indicating that β-catenin mediated transcriptional activation by TCF/LEF in this cellular model cannot be rescued by alternative proteins or pathways.

### Growth characteristics of β-catenin deficient clones

**Figure 5:**
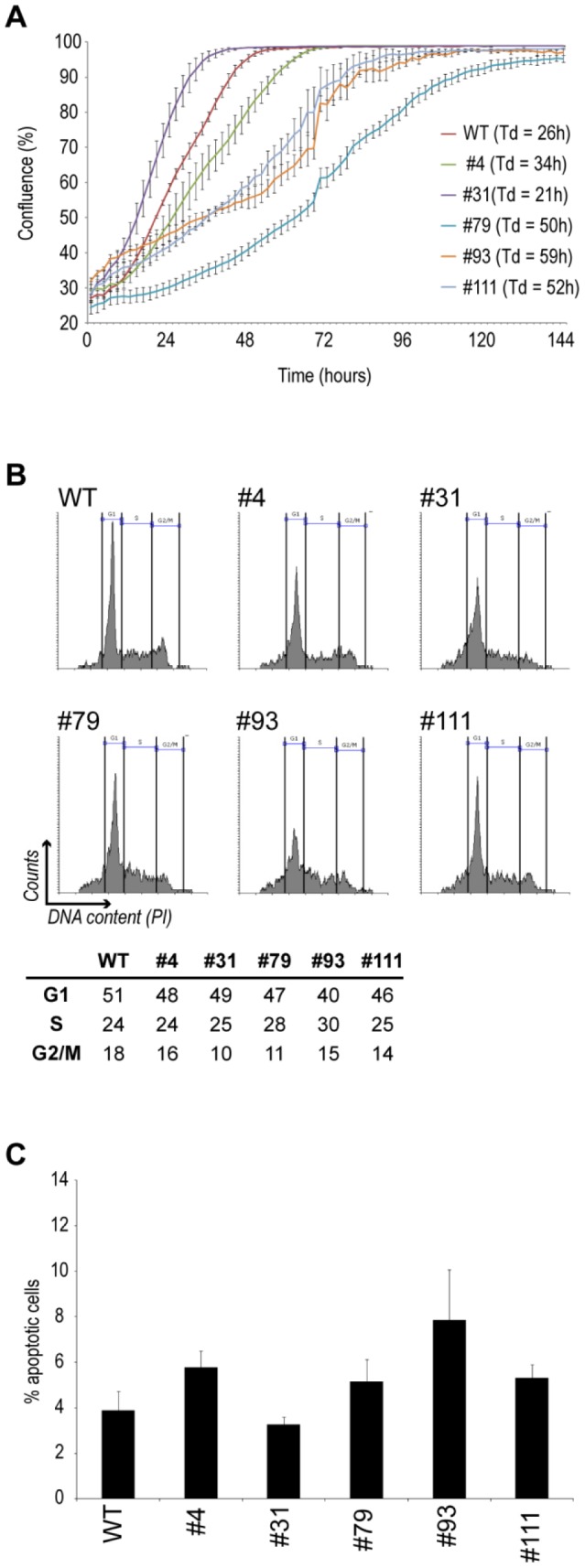
Proliferation rates, cell cycle distribution and apoptosis levels in wild type and *CTNNB1* gene disrupted cells. A) Analysis of proliferation rates in wild type and gene disrupted clones by the IncuCyte live cell imaging system as described in Materials and Methods. The calculated doubling time (Td) for each clone is also shown. B) Cell cycle distribution of WT and gene disrupted clones by quantification of cellular DNA amount using PI staining and flow cytometry. The percentage of cells in the different cell cycle phases (G1, S and G2/M) is shown. C) Quantification of apoptosis levels in WT and gene disrupted clones as indicated. Shown are the percentage AnnexinV-FITC positive cells as determined by flow cytometry. Error bars represent SD between three parallels.

To examine whether the depletion of β-catenin affected cell growth, cells were grown in an IncuCyte live cell imaging system. As seen in Fig. 5A, the β-catenin deficient clones showed a range of proliferation rates; Clone #31 and #4 displayed doubling times (Tds) (21 and 34 hrs, respectively) that are moderately variant from the wild type cells (26 hrs) while clones #79, #93 and #111 exhibited reduced proliferation rates with Tds more than twice as long (50, 59 and 52 hrs respectively) as observed in the wild type cells. The reduced growth rate observed in clone #79, #93 and #111 could not be coupled to significant changes in a specific cell cycle phase, as all the BxPC3ΔCTNNB1 clones displayed a similar cell cycle distribution of (Fig. 5B).

To test whether mutant BxPC-3 clones showed altered levels of apoptosis, an AnnexinV assay was carried out. As seen in Fig. 5C, levels of apoptosis were in principle similar in wild type and all the BxPC3ΔCTNNB1 clones, suggesting that in BxPC-3 cells β-catenin did not have a major influence on cellular viability.

Overall, these data suggest that β-catenin is not essential for normal survival in BxPC-3 cells although its depletion can lead context dependent to reduced proliferation rates.

### Global expression profiling of *CTNNB1* gene disrupted clones

**Figure 6:**
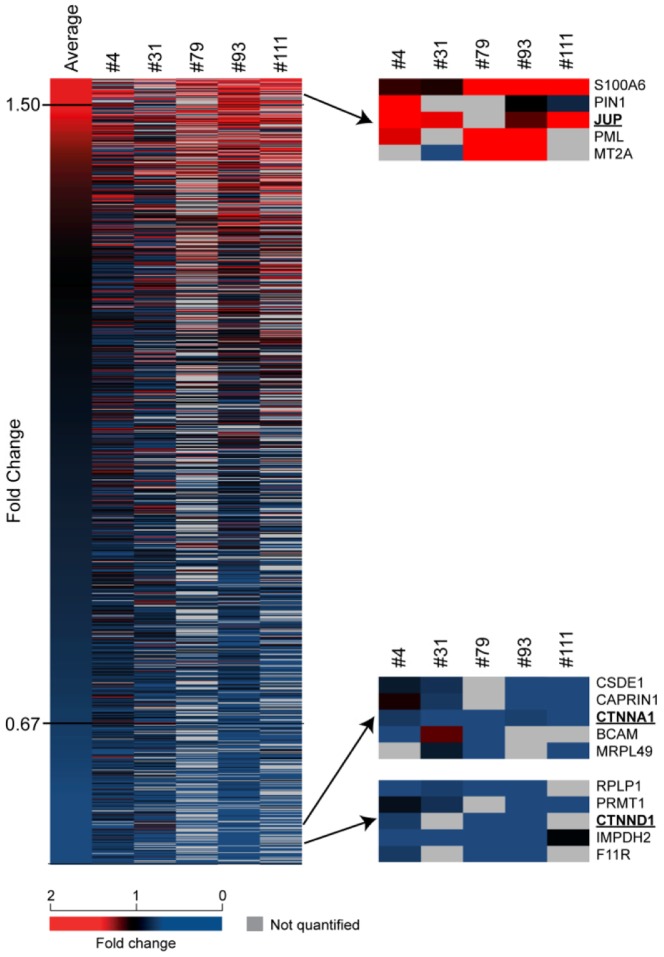
Heat map of protein abundance in the β-catenin deficient clones relative to wild type cells as determined by comparative SILAC analysis. The heat map was generated from all proteins that were detected and quantified by SILAC in at least three of the five β-catenin deficient clones. The position of the key adherens junction proteins JUP (plakoglobin), CTNNA1 (α-catenin) and CTNND1 (p120-catenin) is indicated and enlarged. A fully annotated version of the heat map is included in S4 Table. Up regulated proteins are in blue, down regulated proteins are in red and not quantified proteins are in grey as indicated in the color legend below.

With the aim of providing an overview over potential global changes in protein expression levels in the β-catenin deficient clones, relative protein quantification using stable isotope labeling by amino acids in cell culture (SILAC) was performed [23]. In this analysis the protein levels in the individual β-catenin deficient clones (#4, #31, #79, #93 and #111) were quantified relative to the protein levels in wild type cells on a global scale. In Fig. 6 a heat map representing the abundance of the proteins that were quantified in at least three of the five β-catenin deficient clones is shown. In neither of the *CTNNB1* gene disrupted clones β-catenin protein was detected. This was most likely due to exclusion from the list of quantified proteins, since for proteins that are absent or at very low levels in either of the heavy or light labeled cell lysates, a ratio of peak intensities will not be generated in the SILAC analysis and the protein will thus be excluded. To identify relevant shared biological functions associated with the up and down regulated proteins (<0.67 or>1.5 fold change), Gene Ontology (GO) term enrichment analysis was performed on those differentially expressed proteins that were quantified in at least three of the five *CTNNB1* gene disrupted clones. For the 45 up regulated proteins eight significantly enriched GO terms were identified while for the 100 down regulated proteins 16 significantly enriched GO terms were identified (Table 1 and 2, respectively). Importantly, the GO term that displayed the most significant enrichment was “Cell adhesion” (Bonferroni adjusted P value: 0.017) and included 15 of the proteins that were down regulated in the mutant clones (the identity of all proteins included in the enriched GO terms are shown in S1 Table).

**Table 1:** Ontology enrichment analysis of proteins quantified by SILAC as up regulated in at least three of the β-catenin deficient clones (average fold change>1.5).

Term (GOTERM_BP_2)	Number of proteins	Bonferroni P value
Cellular macromolecular complex subunit organization	6	0.217
Macromolecular complex subunit organization	8	0.220
Regulation of cellular component organization	6	0.504
Cellular component assembly	8	0.567
DNA packaging	3	0.976
Interspecies interaction between organisms	4	0.979
Cell death	6	0.984
Protein complex biogenesis	5	0.988

**Table 2:** Ontology enrichment analysis of proteins quantified by SILAC as down regulated in at least three of the β-catenin deficient clones (average fold change <0.67).

Term (GOTERM_BP_2)	Number of proteins	Bonferroni P value
Cell adhesion	15	0.017
Cellular component assembly	15	0.162
Cellular macromolecular complex subunit organization	9	0.214
Macromolecular complex subunit organization	12	0.486
Biosynthetic process	35	0.508
Interspecies interaction between organisms	7	0.677
Macromolecule localization	14	0.901
Establishment or maintenance of cell polarity	3	0.990
DNA packaging	4	0.990
Cellular component morphogenesis	7	0.993
Regulation of metabolic process	32	0.995
Cell junction organization	3	0.998
Cell cycle	10	0.999
Maintenance of location	3	0.999
Actin filament-based process	5	1.000
Cellular localization	11	1.000
Organelle organization	14	1.000

In the SILAC analysis, the proteins identified to be associated with cell adhesion included components of integrins (ITGA3, ITGAV and ITGB1), laminins (LAMB1), focal adhesion (LPXN, PPFIBP1), tight junctions (F11R) and desmosomes (DSG2 and PKP1) (S1 Table). Moreover, three key components of the adherens junction complex were found to be differentially expressed in the β-catenin deficient clones; α-catenin (CTNNA1) and p120-catenin (CTNND1) were found to be downregulated while plakoglobin (JUP) was identified as being up regulated in the SILAC analysis (Fig. 6).

To analyze if β-catenin depletion led to changes in the global transcription levels in the BxPC-3 cells, cDNA microarray analysis was carried out. Comparison of the transcription levels in wild type cells with two of the *CTNNB1* gene disrupted clones (#4 and #111) identified 85 transcripts that were the most differentially regulated (fold change>2, q-value = 0, S2 Table). When GO term enrichment analysis was performed on these transcripts the most significantly enriched GO term was again “Cell adhesion” (Bonferroni adjusted P value: 0.46, S3 Table). Even though “Cell adhesion” was also found to be the most enriched GO term in the SILAC results (Table 2), there was no overlap between the transcripts and the proteins classified in the “Cell adhesion” GO term (S1 and S2 Tables). When the all 85 most differential regulated transcripts from the microarray analysis was compared with the list of proteins that were quantified in at least three of the five gene disrupted clones from the SILAC, only MET was found to be differentially (down) regulated both on transcription and protein level. Interestingly, in colon cancer cell lines MET has been identified as a direct transcriptional target of β-catenin [24] and in pancreatic cancer MET has been recognized as a cancer stem cell marker [25]. Notably another known target of WNT signaling, NEDD9 (HEF1) was also found to be down regulated in the microarray analysis of mutant BxPC3ΔCTNNB1 clones (S2 Table). NEDD9 is an adhesion docking molecule and high levels of NEDD9 has been observed in many cancer types including PA [26], [27]. Thus, although we could not detect basal activation of TCF/LEF mediated transcription in the STF/SFF reporter gene assay (Fig. 4), wild type BxPC-3 cells still transcribes β-catenin downstream target genes that are not longer transcribed in BxPC3ΔCTNNB1 cells. In summary the results from the global expression profiling in BxPC-3 cells reflects a structural and adhesive function of β-catenin as well as a regulatory function in the transcription of components of adhesive functions.

### Loss of β catenin is compensated by increased levels of plakoglobin protein at the adherens junctions

The results from the SILAC analysis (Fig. 6, Tables 1 and 2) together with the observed localization of β-catenin at the cell membranes (Fig. 1B) and hardly detectable levels of WNT signaling (Fig. 4) indicated that in BxPC-3 cells a core function of β-catenin is at the adherens junctions. Adherens junctions comprises centrally of the transmembrane protein E-cadherin. In epithelial adherens junctions the extracellular domain of the transmembrane protein E-cadherin establishes Ca^2+^ dependent interactions with neighboring cells [28]. β-catenin or plakoglobin binds to the intracellular domain of E-cadherin and connects E-cadherin to the actin cytoskeleton via α-catenin. Interactions with a further members of the armadillo family - p120-catenin - regulates E-cadherin stability at the cytoplasmic shaft of E-cadherin [28]. The close β-catenin homolog, plakoglobin (γ-catenin) shares multiple interaction partners with β-catenin and it has been shown that in adherens junctions β-catenin and plakoglobin can bind to E-cadherin in a mutually exclusive manner [29].

**Figure 7:**
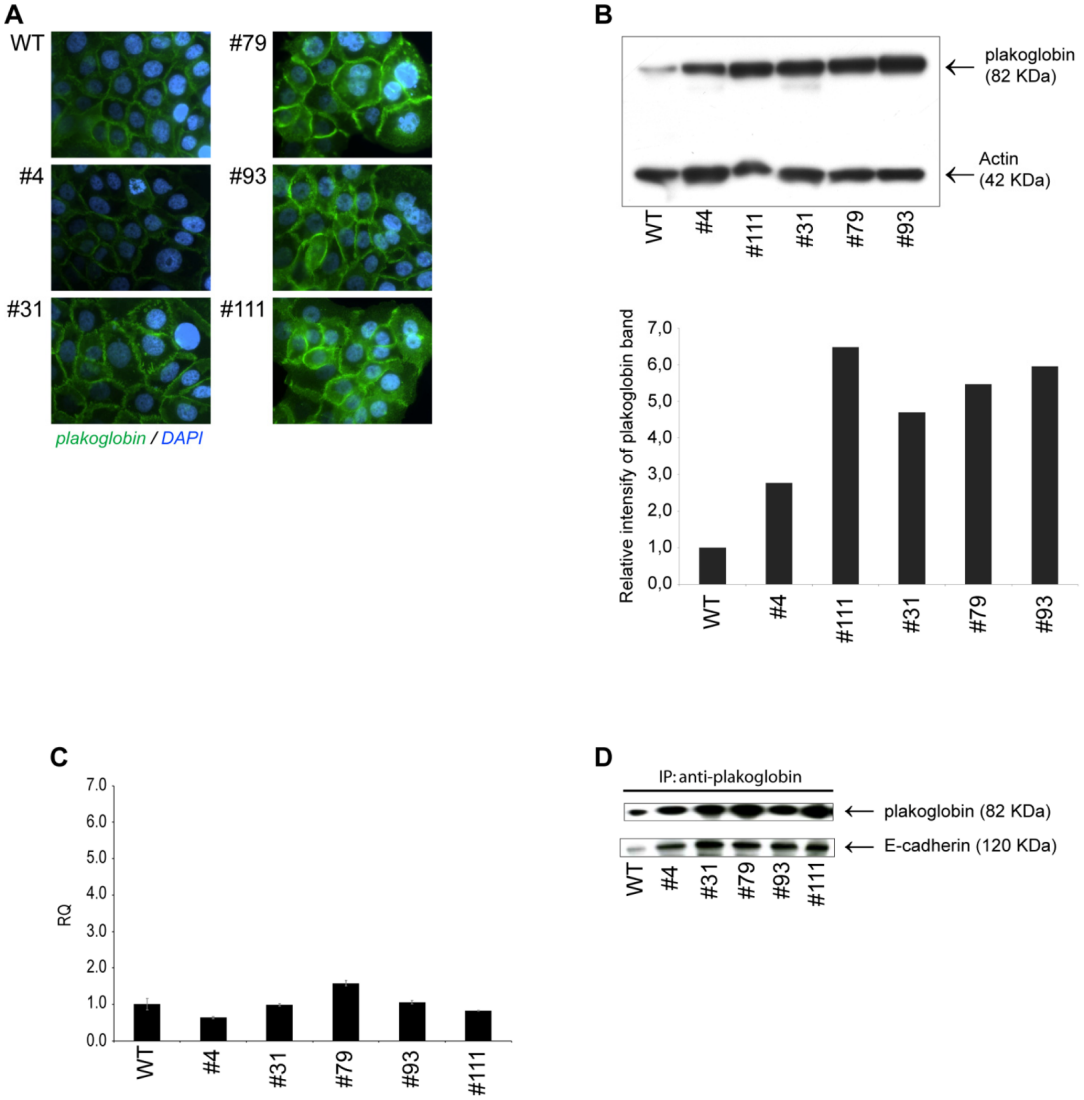
*CTNNB1* gene disrupted cells displays increased levels of plakoglobin protein. A) Immunostaining of WT and gene disrupted clones with plakoglobin Ab (green) and DAPI nuclear counter stain (blue). B) WB analysis of total cell lysates from wild type and gene disrupted clones. Position and molecular weight of plakoglobin and actin specific bands are indicated. Quantification of the intensities of the plakoglobin bands relative to the actin bands are shown below. C) Relative quantification of plakoglobin mRNA levels using qRT-PCR in WT and gene disrupted clones. Error bars represent SD of the mean RQ value. D) Co-immunoprecipitation of total cell lysates from WT or gene disrupted cells using anti plakoglobin Ab for immunoprecipitation and anti plakoglobin and anti E-cadherin Abs for detection as indicated.

Both in the wild type and β-catenin deficient cells plakoglobin was localized at the cell membranes (Fig. 7A) and, as seen in Fig. 7B, quantitative WB analysis was in agreement with the results from the SILAC analysis demonstrating that the protein levels of plakoglobin were distinctly increased in the clones that were devoid of β-catenin. In a quantitative WB analysis clone #4 displayed a 2.8 fold increase in the plakoglobin protein levels while in the rest of the β-catenin deficient clones (clone #31, #79, #93 and #111) the protein levels of plakoglobin were increased by 4.8 to 6.5 fold. The increased levels of plakoglobin protein were not a result of altered transcriptional activation as qRT-PCR analysis established that the levels of plakoglobin mRNA were similar in the wild type cells and all the β-catenin deficient clones (Fig. 7C). Thus regulation of protein stability is most likely the underlying mechanism responsible for the observed increased plakoglobin protein levels in the β-catenin deficient cells.

To verify that plakoglobin was substituting for β-catenin at the adherens junctions of β-catenin deficient clones and hence interacted functionally with E-cadherin, co-immunoprecipitation experiments were performed. When whole cell lysates were immunoprecipitated with anti-plakoglobin Abs, E-cadherin could be detected in the precipitated protein complex in both the wild type and all the β-catenin deficient clones (Fig. 7D) confirming an interaction between the two proteins. The levels of immunoprecipitated E-cadherin correlated well with the levels of plakoglobin protein present in the cells with lowest levels in wild type cells and increased levels of immunoprecipitated E-cadherin in the cells deficient of β-catenin.

**Figure 8:**
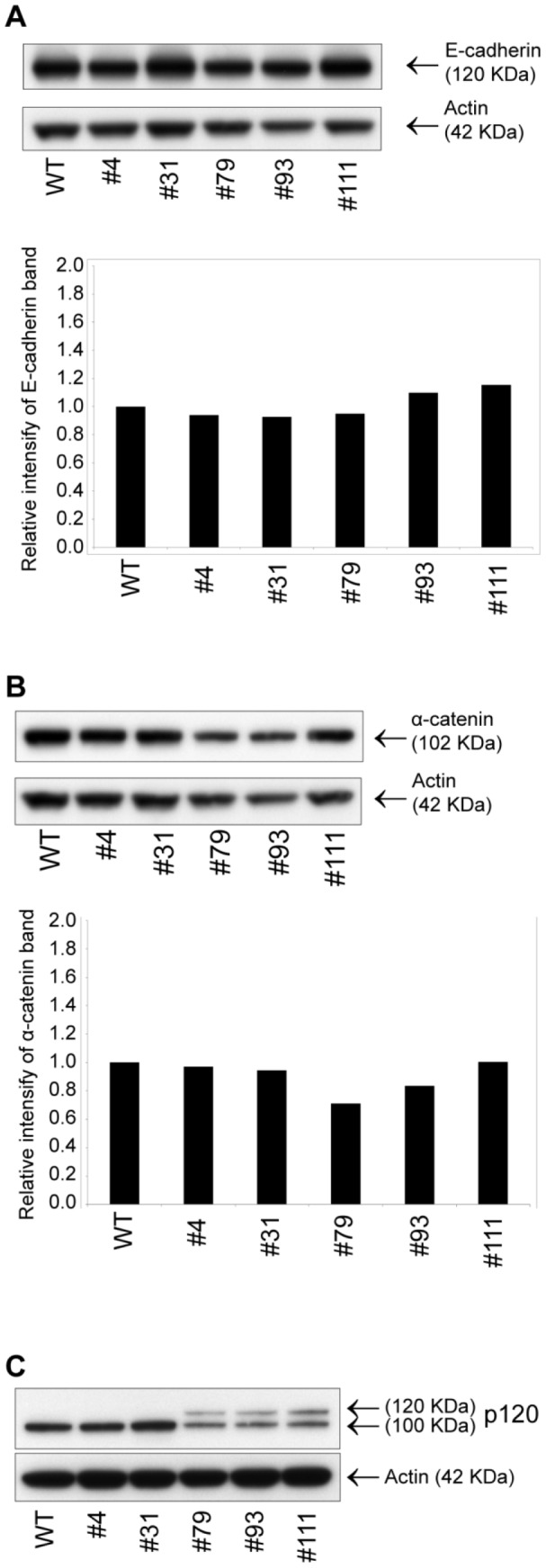
WB analysis of E-cadherin, α-catenin and p120-catenin in the *CTNNB1* gene disrupted clones. A) Quantitative WB analysis on total cell lysates (10 µg TP) from WT and gene disrupted clones using anti E-cadherin and anti actin Abs. Shown are E-cadherin and actin bands with the relative quantification below. B) Quantitative WB analysis as in A using anti α-catenin and anti actin Abs. C) WB analysis using anti p120-catenin and anti actin Abs as in A.

To determine if the core composition of adherens junctions was influenced by the complete loss of β-catenin, quantitative WB analysis on E-cadherin, α-catenin and p120-catenin was carried out. No changes in the protein levels of E-cadherin and α-catenin could be observed between the wild type and β-catenin deficient cells (Fig. 8A and 8B respectively). Notably, α-catenin was found to be down regulated in the SILAC analysis (Fig. 6). The observed discrepancy between SILAC and WB quantification of α-catenin was unexpected but may be due to intrinsic differences in methodology and sensitivity. WB analysis of p120-catenin revealed the presence of an additional longer isoform of the protein in the β-catenin deficient clones #79, #93 and #111 (Fig. 7C). In adherens junctions, p120-catenin binds to the intracellular juxamembrane domain of E-cadherin preventing internalization and degradation of E-cadherin, thus stabilizing cell adhesion [30], [31]. It is known that N-terminal splicing events can lead to expression of the p120-catenin isoforms 1-4 [32] and although the precise role of the various p120-catenin isoforms is unclear, it has been shown that the full length isoform 1 (long isoform) is predominantly expressed in highly motile cells while the truncated isoform 3 (short isoform) is abundant in epithelial cell lines [33].

Interestingly, the three β-catenin deficient clones (#79, #93 and #111) with a shift towards expression of both the long and the short p120-catenin isoforms were the same clones that displayed reduced proliferation rates (Fig. 5A).

### In the absence of β-catenin, plakoglobin is indispensable for formation of cell-cell adhesions

**Figure 9:**
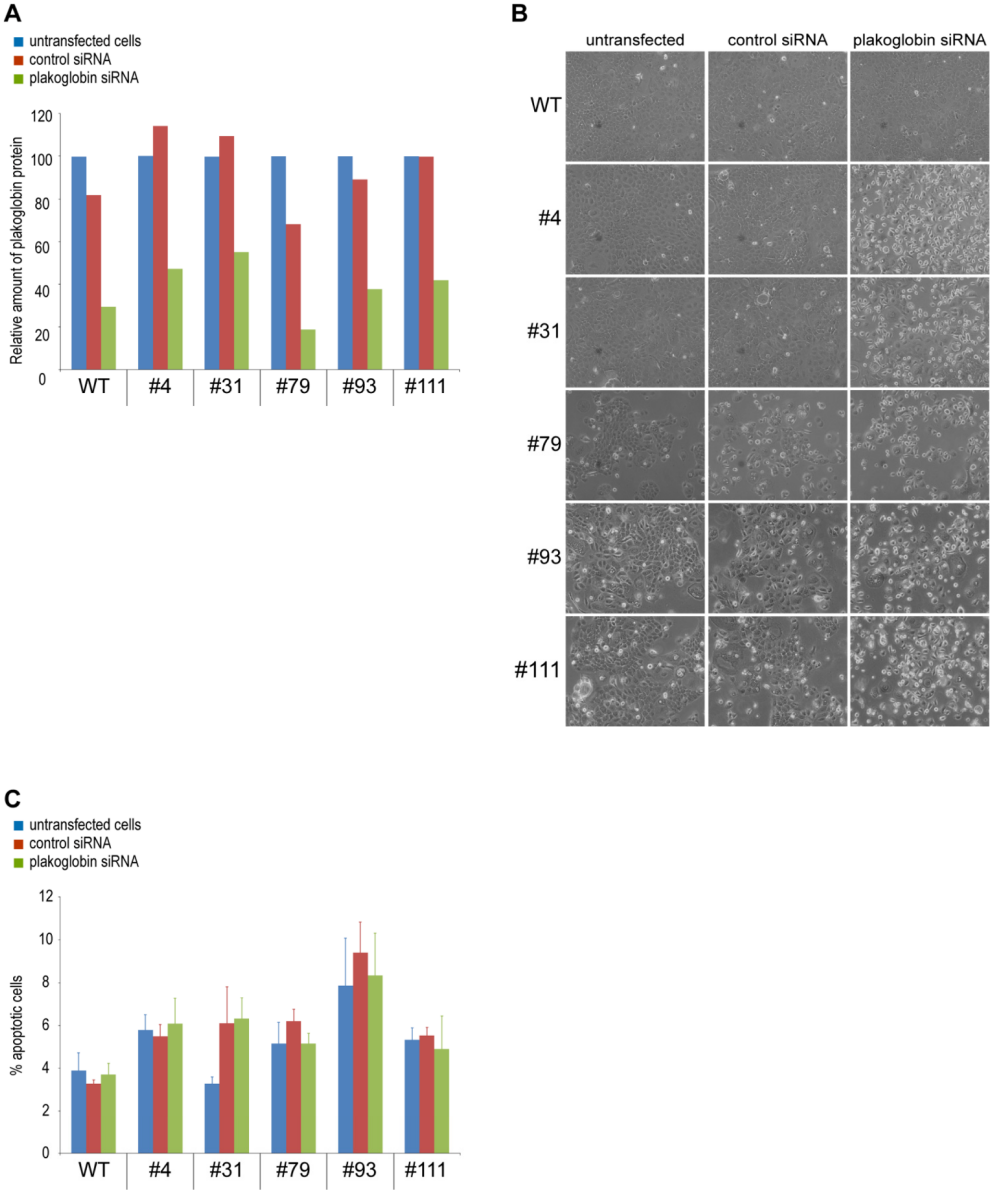
BxPC-3 cells deficient of both β-catenin and plakoglobin cannot form normal cell to cell connections. A) Quantification of plakoglobin protein levels in wild type and gene disrupted cells after transfection with control or plakoglobin siRNA. Cell lysates were harvested 72 hours after transfection and analyzed by quantitative WB. The values are normalized so that the levels of plakoglobin protein in untransfected cells are set to 100%. B) Morphology of untransfected, control and plakoglobin siRNA transfected wild type and gene disrupted clones. Phase contrast images were acquired 72 hours after siRNA transfection. C) Quantification of apoptosis levels in untransfected, control and plakoglobin siRNA transfected wild type and gene disrupted clones. Shown are the percentage AnnexinV-FITC positive cells as determined by flow cytometry. Error bars represent the SD between three parallels.

Given that plakoglobin complemented for the loss of β-catenin in adherens junctions (Fig. 7D), we next wanted to analyze the effect of reducing the amount of plakoglobin in the β-catenin deficient cells by plakoglobin siRNA treatment. As seen in Fig. 9A, cells that were treated with plakoglobin siRNA, showed a 45–81% reduction of plakoglobin protein levels. Wild type cells that had been treated with either control siRNA or plakoglobin siRNA did not display visible morphological changes and formed normal cell to cell connections (Fig. 9B, upper panel). In contrast, treatment of the β-catenin deficient clones with plakoglobin siRNA resulted in obvious morphological changes (Fig. 9B). All β-catenin deficient clones with plakoglobin siRNA mediated knockdown displayed cells with a rounded appearance having lost the ability to form normal contact with the neighboring cells thus showing a morphology compatible of cells with disrupted adherens junctions. Interestingly, the altered cell-to-cell contacts in these cells did not alter cell viability and the level of apoptosis was similar in untreated, control siRNA and plakoglobin siRNA treated cells (Fig. 9C).

### Analysis of the key adherens junction components in cells deficient for both β-catenin and plakoglobin: Internalization of E-cadherin and reduced levels of α-catenin and p120-catenin

**Figure 10:**
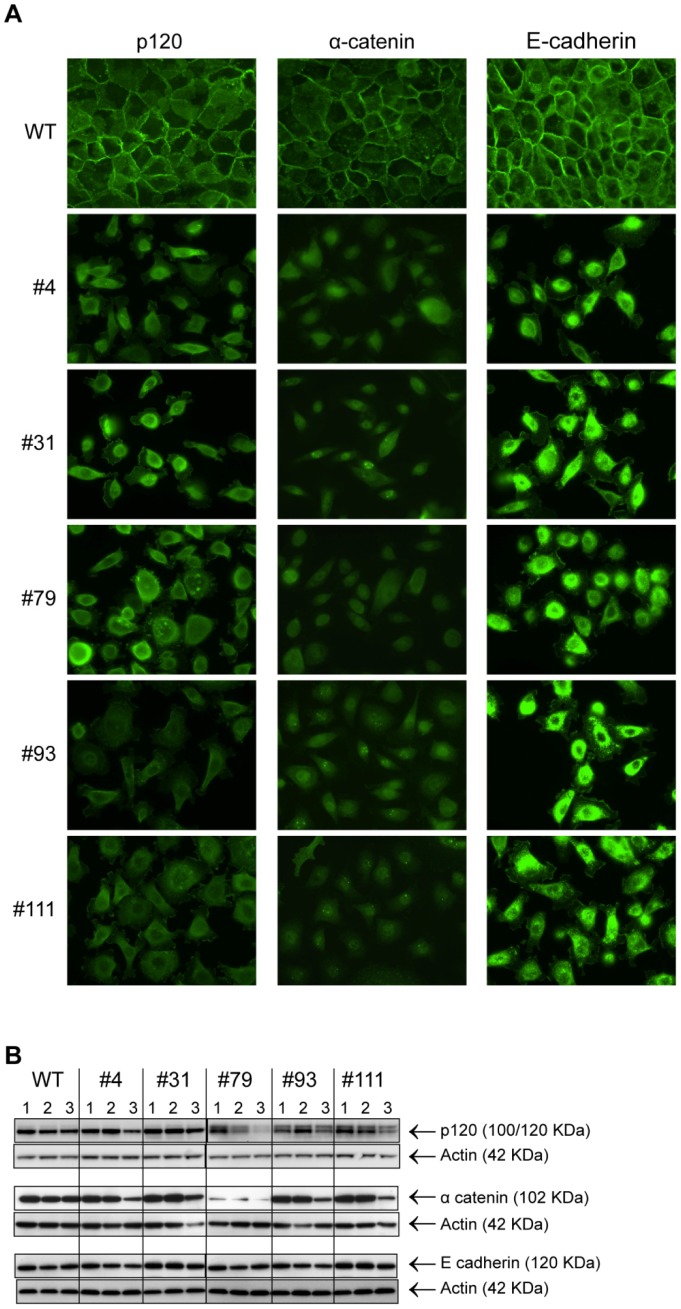
Analysis of protein localization and protein levels of p120-catenin, α-catenin and E-cadherin in cells deficient of both β-catenin and plakoglobin. A) Immunostaining of plakoglobin siRNA transfected wild type and gene disrupted cells as indicated. The cells were stained with anti p120-catenin (left column), anti α-catenin (middle column) and anti E-cadherin (right column) Abs 72 hours after siRNA transfection. Immunostaining (p120-catenin, α-catenin and E-cadherin) of untransfected and control siRNA transfected cells are included in S1 Fig. B) WB analysis of total cell lysates (10 µg TP) from untransfected (1), control siRNA (2) and plakoglobin siRNA (3) transfected wild type and gene disrupted cells as indicated. The cell lysates was prepared 72 hours after siRNA transfection and specific bands for p120-catenin (upper panel), α-catenin (middle panel) and E-cadherin (lower panel) with the corresponding actin bands are shown as indicated.

To further characterize the underlying mechanisms for the observed morphological changes in the BxPC-3 cells lacking both β-catenin and plakoglobin the localization and the amount of the key adherens junction proteins α-catenin, p120-catenin and E-cadherin was examined by immunostaining and WB analysis respectively. Immunostaining of p120-catenin revealed that following treatment of the β-catenin deficient clones with plakoglobin siRNA the localization of p120-catenin was changed from predominantly membranous to cytoplasmic (Fig. 10A left column). Furthermore, the plakoglobin siRNA treated β-catenin deficient cells displayed reduced amounts of p120-catenin protein in some sub-clones as evident both in immunostaining and in WB analysis (Fig. 10A left column and Fig. 10B upper panel). The change in localization and protein amounts was only observed in cells deficient for both β-catenin and plakoglobin and not in cells that were deficient for either β-catenin or plakoglobin (Fig. 10A, 10B and S1A Fig.).

When the localization and protein levels of α-catenin was analyzed in cells deficient for both β-catenin and plakoglobin, the result was similar to the analysis of p120-catenin, with loss of membranous localization and reduced protein levels in some sub-clones (Fig. 10A middle column and 10B middle panel and S1B Fig.). Also the localization of E-cadherin was comparably changed in cells deficient for both β-catenin and plakoglobin (Fig. 10A right column). However, WB analysis of E-cadherin revealed no changes in protein levels regardless if the cells were deficient for β-catenin, plakoglobin or both (Fig. 10B, lower panel). Cells deficient for both β-catenin and plakoglobin displayed accumulation of E-cadherin in intracellular puncta (Fig. 10A right column) that were not observed in cells deficient for solely β-catenin or plakoglobin (Fig. 10A right column and S1C Fig.).

In summary, the combined loss of β-catenin and plakoglobin in BxPC-3 cells was associated with a reduction of p120 and α-catenin together with an internalization of E-cadherin leading to an impaired ability to form normal cell to cell contact, but not affecting overall cell growth under the tested conditions.

## Discussion

Characterization of the molecular mechanisms and pathways involved in tumorigenesis is important for the successful advancement of new treatment regimens. WNT/β-catenin signaling is one of the key pathways that have been shown to be mutated in most cases of PA; nonetheless the precise roles of β-catenin, the central mediator of canonical WNT signaling are not entirely understood [8]. In this study we aimed to examine the role of β-catenin in PA by creating cell lines that were rendered entirely deficient of β-catenin. To accomplish this, ZFN technology was used to create disruptions in the *CTNNB1* gene (β-catenin) coding sequence caused by introduction of site specific genomic DSBs. In all three cell lines that were treated with ZFNs the formation of genomic DSBs as mutations in *CTNNB1* exon 3 could be observed when gDNA from the pool of transfected cells was analyzed 2 days after transfection (not shown), but only form the BxPC-3 cell line viable, β-catenin deficient clones, were obtained. The failure of isolating β-catenin deficient clones in the PANC-03.27 and PANC-1 cell lines could be due to aneuploidy or due to a reliance of the PANC-03.27 and PANC-1 cell lines on functional WNT signaling for cell proliferation and survival. Interestingly, both PANC-1 and BxPC-3 cell lines exhibit low levels of baseline WNT/β-catenin transcriptional activation [9] moreover, while the BxPC-3 cells contains wild type *KRAS* (mutated *KRAS* has been shown to drive pancreatic neoplasia [8], [34]) both the PANC-03.27 and PANC-1 cell lines contain mutations in the *KRAS* gene (COSMIC database [35] and [34]). It remains to be established if PA cell lines where *KRAS* is mutated are more dependent on functional β-catenin for growth and survival.

When using ZFNs or similar gene editing technologies that are based on introducing genomic DSBs, a risk for introducing unwanted genetic changes due to formation of DSBs at off-target sites is present [36], [37]. We did not analyze our β-catenin gene disrupted BxPC-3 clones for the presence off-target changes at a whole genome level. However, sequencing analysis of the *CTNNB1* exon 3 PCR amplicon demonstrated that four of the β-catenin gene disrupted clones (#4, #31, #79 and #93) contained small deletions (1-31 bp) exclusively in the on-target region between the binding sites for ZFN-1 and ZFN-2 (Fig. 2C) while in one of the clones (#111) the whole exon 3 region had been deleted or rearranged (Fig. 2D). The small deletions introduced in BxPC3ΔCTNNB1 clones #4, #31, #79 and #93 would all lead to frameshift mutations with introduction of PTCs in the beginning of the β-catenin coding sequence resulting in β-catenin protein deficiency (Fig. 1B and 1C).

In BxPC3ΔCTNNB1 clone #4 and #79 low levels of a truncated β-catenin protein lacking the NTD could be detected (Fig. 3A, 3B and 3C). In both clone #4 and #79 the levels of β-catenin mRNA was significantly higher compared to the rest of the BxPC3ΔCTNNB1 clones (Fig. 3D). Commonly, mRNA containing PTCs are degraded by conserved mRNA decay pathways to prevent production of potential toxic truncated proteins, [21]. The increased mRNA levels and translation of low levels of a truncated β-catenin protein in clone #4 and #79 could be due to less efficient mRNA degradation and subsequent translation initiation from start codons downstream of the PTCs. Interestingly, the 1088-melanoma cell line expresses a truncated β-catenin protein with similar size as the protein found in clone #4 and #79 [38]. Despite the detection of a truncated β-catenin in clone #4 and #79 both clones displayed similar characteristics as the complete knockout clones (#31, #93 and #111) with non-responsive TCF/LEF transcriptional activation (Fig. 4) and disruption of cell-cell contacts upon plakoglobin knockdown (Fig. 9B). Nonetheless, in the context of targeted gene disruption, translation initiation from start codons downstream of the introduced PTC have to our knowledge not been described before and the possibility for this to occur should be carefully analyzed when applying ZFNs or similar strategies in research or therapeutic applications.

Previous publications have shown that in BxPC-3 cells the baseline levels of WNT/β-catenin mediated transcriptional activation is low [9]. However, it has also been demonstrated that in response to siRNA mediated reduction of β-catenin protein levels, BxPC-3 cells displays reduced proliferation rates and increased levels of apoptosis [39]. In our work the *CTNNB1* gene disrupted BxPC-3 clones did not display increased levels of apoptosis (Fig. 5C) and reduced proliferation rates were only observed in three of the five BxPC3ΔCTNNB1 clones (Fig. 5A). A noticeable difference between protein depletion by siRNA knockdown and targeted gene disruption is levels of target protein reduction. In siRNA strategies obtaining>70% reduction in the protein levels can be difficult [40] while in gene disruption strategies the target protein is typically completely absent. Furthermore, protein depletion by siRNA mediated knockdown is transient while in targeted gene disruption clones originates from individual cells that are isolated and expanded over several passages before experiments can take place. Consequently, it is possible that the here presented BxPC3ΔCTNNB1 clones have acquired adaptive mechanisms in response to the loss of β-catenin protein that would not be present in BxPC-3 cells treated with β-catenin siRNA.

From the global expression analysis “Cell adhesion” was the GO term that was found to be most significantly enriched for both in the results from the SILAC protein analysis and microarray transcript analysis (Tables 1, 2 and S3). In the SILAC analysis the key adherens junction proteins α-catenin (CTNNA1) and p120-catenin (CTNND1) were found to be down regulated while plakoglobin (JUP) was found to be up regulated in the β-catenin deficient clones (Fig. 6). It has previously been established in various mouse cell lines that deletion of β-catenin is compensated for by increased protein levels of plakoglobin that binds to E-cadherin at the adherens junctions [41]–[45]. The same effect has also been demonstrated in human hepatoma cell lines following siRNA mediated reduction of β-catenin protein levels [46]. We observed a prominent increase in the protein levels of plakoglobin in all the BxPC3ΔCTNNB1 clones (Fig. 7B) thus establishing that plakoglobin compensates for the loss of β-catenin also in gene disrupted human PA cells. In concurrence with observations from mice cells [41], [42] the increased plakoglobin protein levels in the BxPC3ΔCTNNB1 cells was not accompanied with increased transcription levels of plakoglobin (Fig. 7C) pointing toward post translational mechanism being causal for the increased plakoglobin levels. In the literature there are contradictory results as to whether plakoglobin can regulate TCF/LEF mediated transcription [42], [47]. In the BxPC3ΔCTNNB1 clones plakoglobin was only detected at the cell membranes (Fig. 7A) and activation of WNT/β-catenin signaling through LiCl_2_ mediated inhibition of GSK3β could only be observed in wild type BxPC-3 cells (Fig. 4). Given that both β-catenin and plakoglobin have been shown to be regulated by GSK3β [48] these results are in accordance with reports demonstrating that plakoglobin alone cannot substitute β-catenin in activation of TCF/LEF regulated transcription. Interestingly however, a recent study on mouse embryonic stem cells (mESC) suggested that a minimum level of β-catenin is required for plakoglobin to be able to stimulate TCF/LEF transcriptional activation [49].

When the abundance of adherens junction proteins was analyzed, SILAC and WB derived data displayed divergent results; In the SILAC analysis α-catenin (CTNNA1) was found to be down regulated (Fig. 6) while in the quantitative WB analysis α-catenin levels was found to be unchanged (Fig. 8B) in the β-catenin deficient clones. Although protein quantification by SILAC and WB are based on fundamentally different methodologies a discrepancy between the results from the two methods was unexpected. Nonetheless, it has been shown that in β-catenin deficient mESC with concurrent increased levels of plakoglobin, levels of α-catenin were reduced and thus weakening mESC cell adhesion [42] while in β-catenin deficient mouse hepatocytes the levels of α-catenin was unchanged [41]. Binding of p120-catenin to E-cadherin is known to be important for retaining E-cadherin at the cell membrane [50] and both the long and short p120-catenin isoforms can bind to E-cadherin [51]. We confirmed in this work that the p120-catenin isoform shift observed in BxPC3ΔCTNNB1 clones #79, #93 and #111 did not influence on the levels of E-cadherin (Fig. 8A and 8C). We do not know the underlying reason for the p120-catenin isoform shift observed in BxPC3ΔCTNNB1 clones #79, #93 and #111, however, the isoform shift could not be related solely to the lack of β-catenin given that it was only observed in a subset of the BxPC3ΔCTNNB1 clones (Fig. 8C). Interestingly, a shift of expression from short to long p120-isoform has been seen during epithelial-to-mesenchymal transition [33].

Adherens junctions are dynamic structures with the ability to continuously being formed and disassembled whereby the main mechanism controlling adherens junction homeostasis is the regulation of the amount of E-cadherin present at the cell membrane [28]. Endocytosis is an important mechanism for regulating E-cadherin turnover and it has been demonstrated that binding to β-catenin and most likely plakoglobin, prevents proteosomal degradation by shielding of the PEST sequence motif on E-cadherin [52], [53]. When the protein levels of plakoglobin were reduced in the BxPC-3 cells by siRNA treatment, all the β-catenin deficient clones displayed a dispersed morphology with loss of cell to cell contacts (Fig. 9B). While the amount of p120-catenin and α-catenin were reduced in these cells, the protein levels of E-cadherin remained largely unchanged in plakoglobin siRNA treated BxPC3ΔCTNNB1 clones (Fig. 10B and S1 Fig.). However, immunostaining revealed that the localization of E-cadherin was changed from membranous to accumulation in cytoplasmatic puncta in cells that were deficient of both plakoglobin and β-catenin (Fig. 10A). Thus, our data using human BxPC-3 cells confirm previous studies performed in mice cells demonstrating a role for β-catenin or plakoglobin in trafficking of E-cadherin to the cell membrane [42], [44]. Our results show overall unchanged E-cadherin protein levels in cells that were deficient of both β-catenin and plakoglobin. This diverges from previous studies where a reduction of E-cadherin protein levels has been observed in cells that are deficient of both β-catenin and plakoglobin. Fukunaga et al. [44] demonstrated reduced E-cadherin levels in mouse F9 teratocarcinoma cells in which both the β-catenin and the plakoglobin gene had been knocked out at a genomic level, Lyashenko et al. [42] showed reduction of E-cadherin protein levels in β-catenin knockout mESC upon treatment with plakoglobin siRNA while in the publication by Wickline et al. [46] reduction of β-catenin and plakoglobin levels in Hep3B human heptoma cells by siRNA treatment led to reduced E-cadherin protein levels. The reason for the difference between these studies and our findings may be technical, e.g. the remaining amounts of plakoglobin after siRNA treatment are sufficient to protect E-cadherin form degradation, or contextual. The latter is currently being further investigated.

The data presented in this work help to identify core functions of β-catenin in PA cells, and highlight compensatory mechanisms that the tumor cells can activate to attenuate the effect of a β-catenin depletion. This has implications for designing therapeutic strategies based on selective pathway inhibitors.

## Supporting Information

S1 Fig **Immunostaining (p120-catenin, α-catenin and E-cadherin) of plakoglobin siRNA treated WT and CTNNB1 gene disrupted clones.**
(PDF)

S1 Table **Gene Ontology enrichment analysis of proteins quantified by SILAC to be up or down regulated in at least three of the β-catenin deficient clones.**
(PDF)

S2 Table **List of the most differentially regulated probes from significance analysis of microarrays (SAM) comparing wild type BxPC3 cells and the gene disrupted clones #4 and #111 (average).**
(PDF)

S3 Table **Gene Ontology enrichment analysis of the most differentially regulated transcripts between the wild type BxPC3 cells and the β-catenin deficient clones #4 and #111 (average).**
(PDF)

S4 Table **Complete annotated heat map generated from the proteins identified and quantified by SILAC in at least three β-catenin deficient clones.**
(PDF)

S5 Table **List of all proteins detected and quantified by SILAC in the individual β-catenin deficient clones.**
(XLSX)

S1 Materials and Methods **Quantitative proteonomics by SILAC.**
(DOCX)
