# Supplementary material for: Implications of Targeted Genomic Disruption of β-Catenin in BxPC-3 Pancreatic Adenocarcinoma Cells
Source: PLoS One. 2014 Dec 23;9(12):e115496. doi: 10.1371/journal.pone.0115496 (PMC4275244; doi:10.1371/journal.pone.0115496)
Supplement: S1 Fig — Immunostaining (p120-catenin, α-catenin and E-cadherin) of plakoglobin siRNA treated WT and CTNNB1 gene disrupted clones. (PDF) [file pone.0115496.s001.pdf]

Supplementary Figure S1

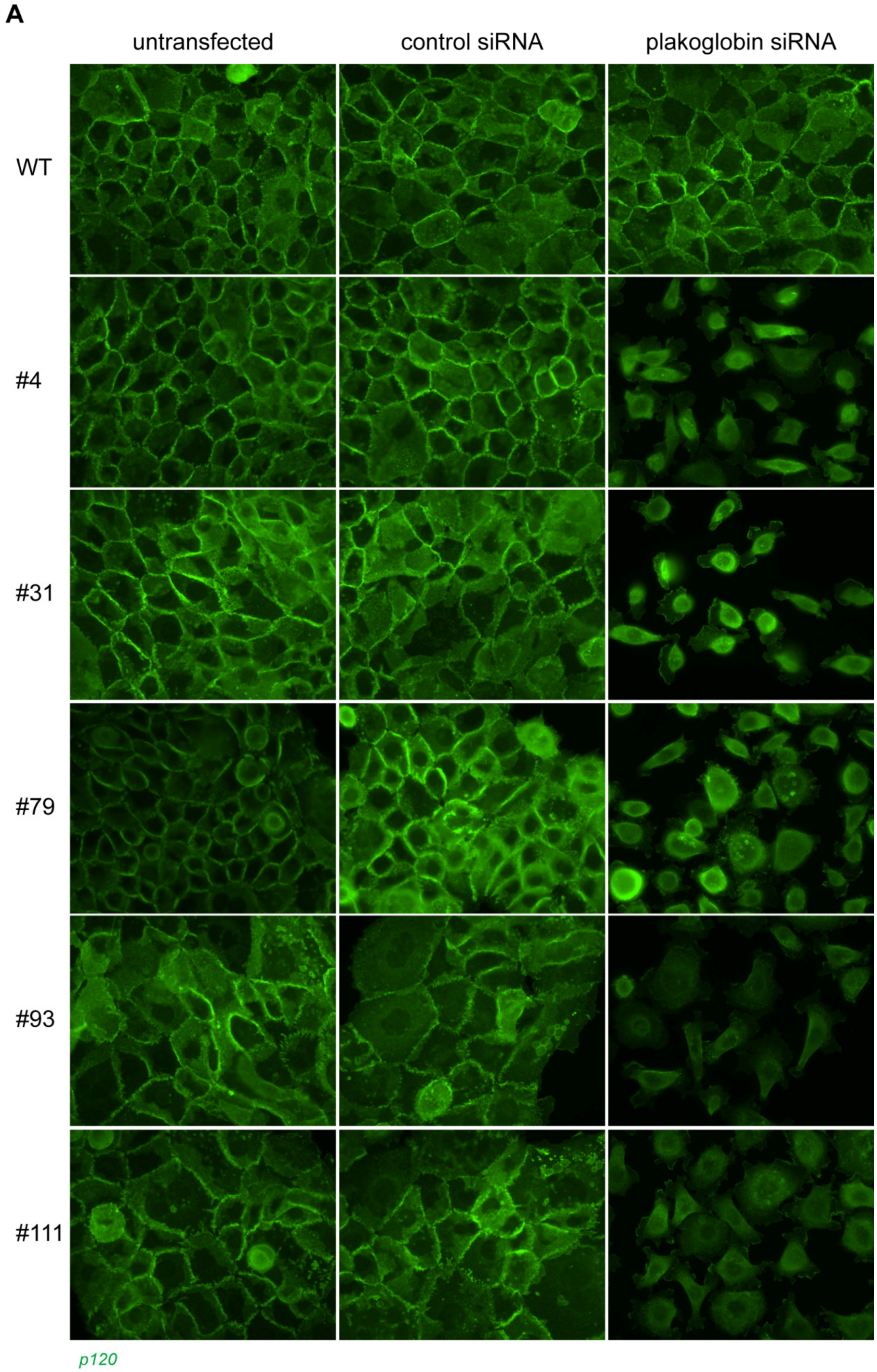

Supplementary Figure S1

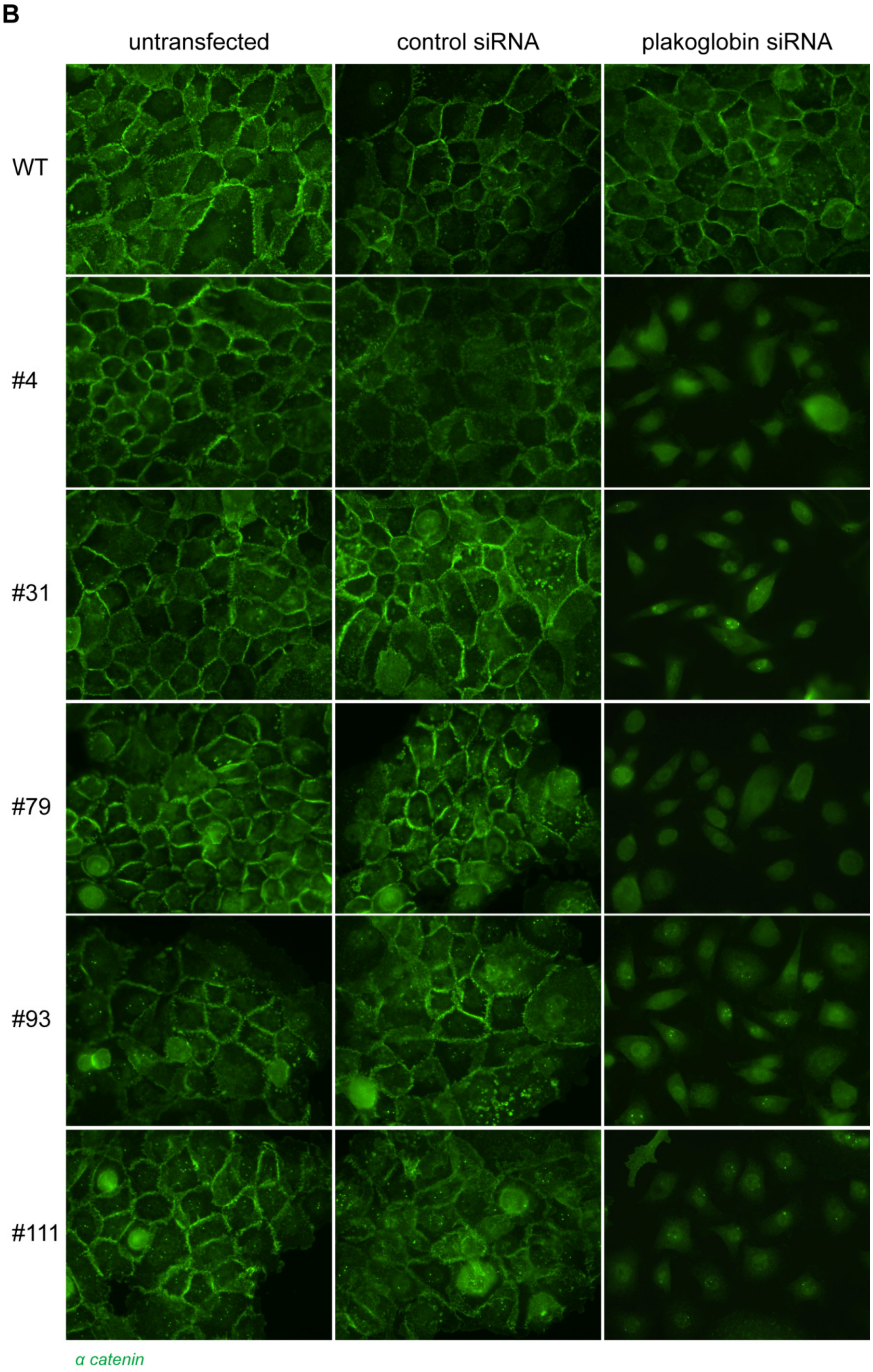

Supplementary Figure S1

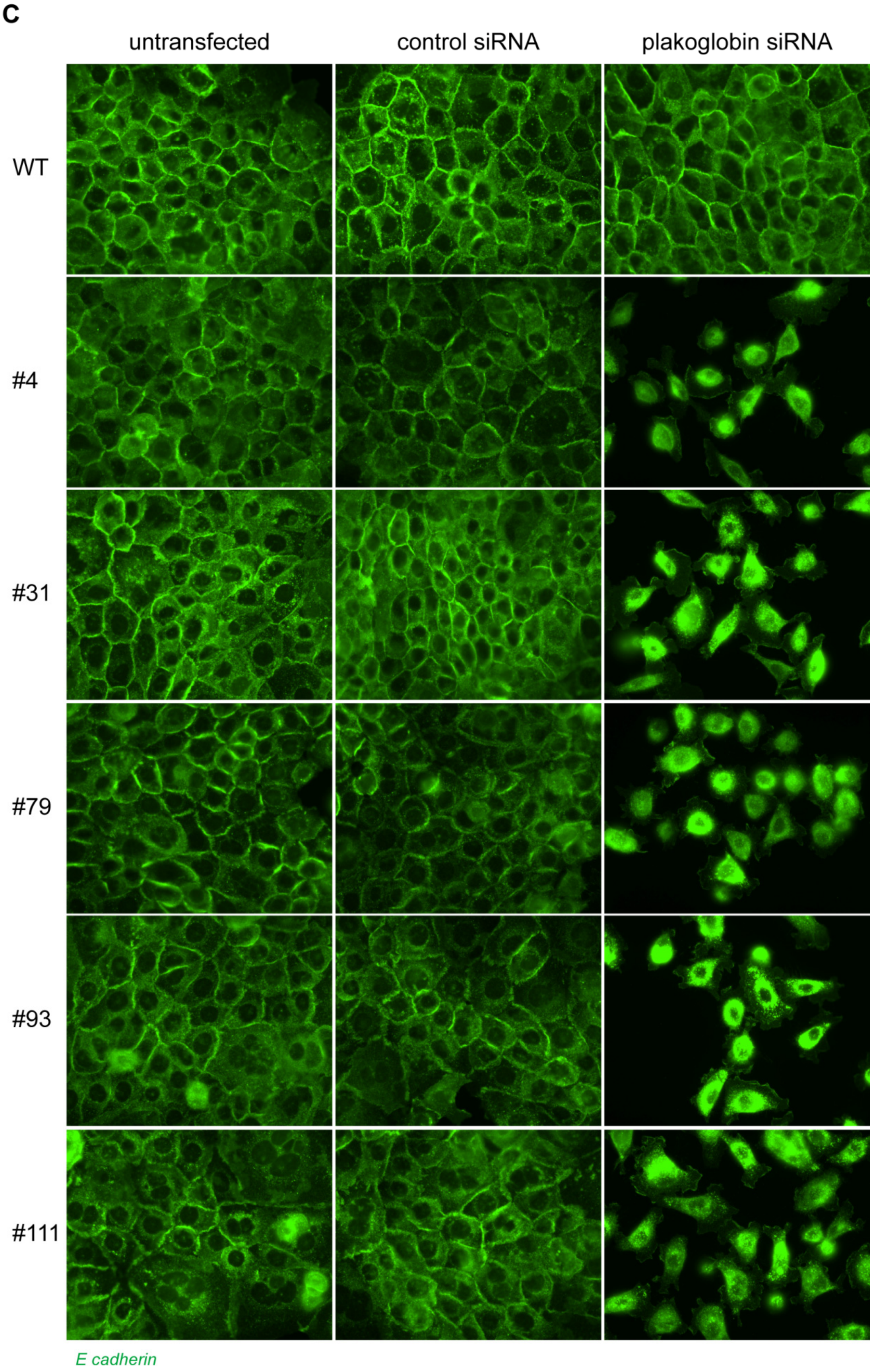

Supplementary Figure S1. Immunostaining of plakoglobin siRNA treated WT and *CTNNB1* gene disrupted clones. A) Immunostaining of untransfected, control and plakoglobin siRNA transfected WT and gene disrupted clones as indicated. The cells were stained with anti p120-catenin Ab 72 hours after siRNA transfection. B) Immunostaining as in A with anti α-catenin Ab. C) Immunostaining as in A with anti E-cadherin Ab.
