# Supplementary material for: Implications of Targeted Genomic Disruption of β-Catenin in BxPC-3 Pancreatic Adenocarcinoma Cells
Source: PLoS One. 2014 Dec 23;9(12):e115496. doi: 10.1371/journal.pone.0115496 (PMC4275244; doi:10.1371/journal.pone.0115496)
Supplement: S1 Table — Gene Ontology enrichment analysis of proteins quantified by SILAC to be up or down regulated in at least three of the β-catenin deficient clones. (PDF) [file pone.0115496.s002.pdf]

Supplementary Table 1.

Gene Ontology enrichment analysis of proteins quantified by SILAC to be up regulated in at least three of the β-catenin deficient clones (average fold change > 1.5).

Category	Term	Count	%	PValue	Proteins	List Total	Pop Hits	Pop Total	Fold Enrichment	Bonferroni	Benjamini	FDR
GOTERM_BP_2	GO:0034621~cellular macromolecular complex subunit organization	6	1,361	0,003	CSE1L, HIST1H4A, HIST2H2BE, GSN, H2AFZ, IPO9	39	357	13945	6,009	0,217	0,217	2,883
GOTERM_BP_2	GO:0043933~macromolecular complex subunit organization	8	1,814	0,003	JUP, CSE1L, HIST1H4A, HIST2H2BE, GSN, PML, H2AFZ, IPO9	39	710	13945	4,029	0,220	0,117	2,934
GOTERM_BP_2	GO:0051128~regulation of cellular component organization	6	1,361	0,008	GSN, PML, SPTBN2, RCC1, AKAP2, PIN1	39	458	13945	4,684	0,504	0,208	8,059
GOTERM_BP_2	GO:0022607~cellular component assembly	8	1,814	0,009	JUP, CSE1L, HIST1H4A, HIST2H2BE, GSN, PML, H2AFZ, IPO9	39	887	13945	3,225	0,567	0,189	9,552
GOTERM_BP_2	GO:0006323~DNA packaging	3	0,680	0,040	HIST1H4A, HIST2H2BE, H2AFZ	39	117	13945	9,168	0,976	0,527	36,156
GOTERM_BP_2	GO:0044419~interspecies interaction between organisms	4	0,907	0,041	VIM, TAP1, PML, STAT1	39	283	13945	5,054	0,979	0,473	36,931
GOTERM_BP_2	GO:0008219~cell death	6	1,361	0,044	CSE1L, GSN, LGALS1, PML, SPTBN2, STAT1	39	719	13945	2,984	0,984	0,444	38,881
GOTERM_BP_2	GO:0070271~protein complex biogenesis	5	1,134	0,048	JUP, CSE1L, GSN, PML, IPO9	39	505	13945	3,540	0,988	0,425	41,195

Gene Ontology enrichment analysis of proteins quantified by SILAC to be down regulated in at least three of the β-catenin deficient clones (average fold change < 0.67).

Category	Term	Count	%	PValue	Proteins	List Total	Pop Hits	Pop Total	Fold Enrichment	Bonferroni	Benjamini	FDR
GOTERM_BP_2	GO:0007155~cell adhesion	15	1,066	0,000	EGFR, F11R, PPFIBP1, CTNND1, ITGA3, BCAM, CTNNA1, ITGB1, ALCAM, LPXN, EZR, DSG2, PKP1, ITGAV, LAMB1	91	700	13945	3,2838	0,017	0,0166	0,168
GOTERM_BP_2	GO:0022607~cellular component assembly	15	1,066	0,002	NCBP1, TLN1, HMGB2, HIST1H1B, IDE, MCM2, CTNNA1, ITGB1, ADRM1, CDC42, LPXN, EZR, GTF2I, IPO5, SMARCA5	91	887	13945	2,5915	0,162	0,0847	1,763
GOTERM_BP_2	GO:0034621~cellular macromolecular complex subunit organization	9	0,640	0,002	ADRM1, NCBP1, HMGB2, HIST1H1B, NASP, IPO5, SMARCA5, ARID1A, MCM2	91	357	13945	3,8632	0,214	0,0772	2,393
GOTERM_BP_2	GO:0043933~macromolecular complex subunit organization	12	0,853	0,006	ADRM1, NCBP1, HMGB2, LPXN, GTF2I, HIST1H1B, NASP, IPO5, IDE, SMARCA5, ARID1A, MCM2	91	710	13945	2,5900	0,486	0,1533	6,470
GOTERM_BP_2	GO:0009058~biosynthetic process	35	2,488	0,006	HMGB2, CNBP, RBM3, EIF5, CTNND1, QARS, CAD, CNOT1, SERPINH1, MTHFD1, RPS26, MCM7, RPLP0, RPLP1, BTF3, RPL10, IMPDH2, EGFR, NASP, PADI3, POLR1C, ARID1A, MCM2, MCM3, MCM4, LPCAT4, ADRM1, RFC4, PSMC5, MRPL28, GTF2I, SMARCA5, MRPL49, PCNA, LRPPRC	91	3542	13945	1,5142	0,508	0,1324	6,887
GOTERM_BP_2	GO:0044419~interspecies interaction between organisms	7	0,498	0,010	F11R, TFRC, RPLP0, ITGAV, IPO5, PDCD6IP, ITGB1	91	283	13945	3,7904	0,677	0,1716	10,732
GOTERM_BP_2	GO:0033036~macromolecule localization	14	0,995	0,020	EGFR, NCBP1, RAB7A, TLN1, NASP, TMSB10, CDC42, NPC2, EZR, MACF1, IPO5, PCNA, PDCD6IP, LRPPRC	91	1076	13945	1,9939	0,901	0,2818	20,774
GOTERM_BP_2	GO:0007163~establishment or maintenance of cell polarity	3	0,213	0,040	CDC42, EZR, MACF1	91	49	13945	9,3821	0,990	0,4367	36,960
GOTERM_BP_2	GO:0006323~DNA packaging	4	0,284	0,040	HMGB2, HIST1H1B, SMARCA5, MCM2	91	117	13945	5,2390	0,990	0,4006	37,052
GOTERM_BP_2	GO:0032989~cellular component morphogenesis	7	0,498	0,043	EGFR, ALCAM, CDC42, EZR, MACF1, LAMB1, ITGB1	91	397	13945	2,7020	0,993	0,3934	39,483
GOTERM_BP_2	GO:0019222~regulation of metabolic process	32	2,274	0,046	HSP90AB1, NCBP1, HMGB2, CNBP, RBM3, IDE, EIF5, CTNND1, CNOT1, PDCD4, CDC42, RPS26, MCM7, ITGAV, CSDE1, BTF3, YAP1, EGFR, CDK6, ARID1A, MCM2, MCM3, MCM4, PSMC5, NPC2, MRPL28, GTF2I, SERBP1, SMARCA5, PCNA, HSPB1, LRPPRC	91	3621	13945	1,3542	0,995	0,3812	41,170
GOTERM_BP_2	GO:0034330~cell junction organization	3	0,213	0,052	CDC42, TLN1, CTNNA1	91	57	13945	8,0654	0,998	0,3970	45,658
GOTERM_BP_2	GO:0007049~cell cycle	10	0,711	0,062	EGFR, MCM7, PSMC5, MACF1, NASP, CDK6, PDCD6IP, MCM2, MCM3, ITGB1	91	776	13945	1,9748	0,999	0,4289	51,895
GOTERM_BP_2	GO:0051235~maintenance of location	3	0,213	0,064	TLN1, EZR, TMSB10	91	64	13945	7,1832	0,999	0,4146	52,918
GOTERM_BP_2	GO:0030029~actin filament-based process	5	0,355	0,070	CDC42, TLN1, EZR, TMSB10, ITGB1	91	241	13945	3,1793	1,000	0,4235	56,396
GOTERM_BP_2	GO:0051641~cellular localization	11	0,782	0,075	EGFR, CDC42, NCBP1, TLN1, EZR, NPC2, MACF1, TXNDC5, IPO5, PCNA, LRPPRC	91	928	13945	1,8164	1,000	0,4253	58,959
GOTERM_BP_2	GO:0006996~organelle organization	14	0,995	0,085	TLN1, HMGB2, HIST1H1B, NASP, ARID1A, TMSB10, MCM2, ITGB1, CDC42, EZR, HUWE1, MACF1, SMARCA5, GOLGB1	91	1332	13945	1,6106	1,000	0,4478	63,735
