# Supplementary material for: Implications of Targeted Genomic Disruption of β-Catenin in BxPC-3 Pancreatic Adenocarcinoma Cells
Source: PLoS One. 2014 Dec 23;9(12):e115496. doi: 10.1371/journal.pone.0115496 (PMC4275244; doi:10.1371/journal.pone.0115496)
Supplement: S2 Table — List of the most differentially regulated probes from significance analysis of microarrays (SAM) comparing wild type BxPC3 cells and the gene disrupted clones #4 and #111 (average). (PDF) [file pone.0115496.s003.pdf]

Supplementary Table 2.
List of the most differentially regulated probes from significance analysis of microarrays (SAM) comparing wild type BxPC3 cells and the gene disrupted clones #4 and #111 (average).
The list was generated by selecting the probes that displayed a fold change >2 and q-value=0 in the SAM analysis of all probes. The list is sorted by the d-score and genes up and down regulated in the gene disrupted clones have a positive and negative fold change value, respectively.

Probe_Id	Symbol	ILMN_GENE	Chromosome	d-score	False Significant Number	Fold Change	q-value
ILMN_1686664	MT2A	MT2A	16	22,155	0	2,45	0
ILMN_1659688	LGALS3BP	LGALS3BP	17	15,656	0	2,358	0
ILMN_2042771	PTTG1	PTTG1	5	14,42	0	2,109	0
ILMN_1672503	DPYSL2	DPYSL2	8	12,37	0	2,368	0
ILMN_2183409	SCARB1	SCARB1	12	-12,283	0	-2,43	0
ILMN_1673356	FAM83C	FAM83C	20	-11,125	0	-2,327	0
ILMN_3247895	LOC728188	LOC728188	X	10,777	0	2,446	0
ILMN_1713147	MCRS1	MCRS1	12	10,703	0	2,178	0
ILMN_1655347	SCGB1A1	SCGB1A1	11	-10,414	0	-2,055	0
ILMN_2320250	NOL6	NOL6	9	-10,404	0	-2,087	0
ILMN_1799098	LOC652846	LOC652846		10,398	0	2,289	0
ILMN_1750324	IGFBP5	IGFBP5	2	-9,748	0	-6,458	0
ILMN_1733756	COL12A1	COL12A1	6	-9,342	0	-2,076	0
ILMN_2145116	TMEM173	TMEM173	5	9,246	0	2,264	0
ILMN_1811972	MYCBP2	MYCBP2	13	-9,226	0	-2,024	0
ILMN_1678707	TAF15	TAF15	17	-9,141	0	-2,081	0
ILMN_1765641	SEMA3A	SEMA3A	7	-9,018	0	-2,024	0
ILMN_1753196	PTTG1	PTTG1	5	8,995	0	2,523	0
ILMN_1673023	EP400	EP400	12	-8,897	0	-2,068	0
ILMN_1765701	LOC399942	LOC399942	11	8,74	0	2,077	0
ILMN_2400759	CPVL	CPVL	7	8,644	0	2,023	0
ILMN_1661366	PGAM1	PGAM1	10	8,606	0	2,736	0
ILMN_1740233	UGT1A10	UGT1A10	2	8,476	0	2,096	0
ILMN_1676358	RALB	RALB	2	8,265	0	2,493	0
ILMN_2321153	MUC4	MUC4	3	-8,15	0	-2,477	0
ILMN_3247578	FAT1	FAT1	4	-8,034	0	-2,08	0
ILMN_2411915	ATG4B	ATG4B	2	7,981	0	2,191	0
ILMN_1754795	FAT1	FAT1	4	-7,879	0	-3,211	0
ILMN_1678757	BCYRN1	BCYRN1	2	-7,775	0	-3,991	0
ILMN_1695917	C5orf15	C5ORF15	5	7,679	0	2,148	0
ILMN_2395389	PSMC4	PSMC4	19	7,197	0	2,627	0
ILMN_2132982	IGFBP5	IGFBP5	2	-7,174	0	-4,242	0
ILMN_1676763	PIPSL	PIPSL	10	7,021	0	2,131	0
ILMN_2109708	ECGF1	ECGF1	22	6,793	0	2,086	0
ILMN_1795778	P4HA2	P4HA2	5	6,646	0	2,211	0
ILMN_2095610	ANXA8	ANXA8	10	6,489	0	2,124	0
ILMN_1691563	GAGE12I	GAGE12I	X	-6,241	0	-2,119	0
ILMN_1704342	UBE3C	UBE3C	7	-6,183	0	-2,136	0
ILMN_1779353	PUS7	PUS7	7	-6,17	0	-2,483	0
ILMN_2326737	PPIE	PPIE	1	6,061	0	2,363	0
ILMN_1800131	LOC652826	LOC652826		6,011	0	2,061	0
ILMN_1788108	TXNDC5	TXNDC5	6	-5,947	0	-2	0
ILMN_2332105	WRNIP1	WRNIP1	6	-5,922	0	-2,222	0
ILMN_1687887	PSMC4	PSMC4	19	5,851	0	2,251	0
ILMN_1685798	MAGEA6	MAGEA6	X	5,832	0	2,07	0
ILMN_1744765	KRT4	KRT4	12	-5,788	0	-3,158	0
ILMN_3308295	MIR205	MIR205		5,548	0	2,083	0
ILMN_3204734	LOC100134648	LOC100134648	7	5,337	0	2,551	0
ILMN_1766762	DYNLRB1	DYNLRB1	20	5,207	0	2,955	0
ILMN_1732074	LOC648210	LOC648210		5,099	0	2,925	0
ILMN_2261076	NEDD9	NEDD9	6	-5,097	0	-2,074	0
ILMN_1681301	AIM2	AIM2	1	5,083	0	2,042	0
ILMN_2371169	ZYX	ZYX	7	-5,063	0	-2,451	0
ILMN_2174127	DCBLD2	DCBLD2	3	-5,038	0	-2,605	0
ILMN_1696187	PYGL	PYGL	14	-5,02	0	-2,216	0
ILMN_1690259	RAE1	RAE1	20	4,98	0	2,121	0
ILMN_1680246	MAT2B	MAT2B	5	4,961	0	3,137	0
ILMN_1798454	MAD2L1BP	MAD2L1BP	6	4,925	0	2,136	0
ILMN_1711702	CLEC2D	CLEC2D	12	4,921	0	2,116	0
ILMN_1753449	CST1	CST1	20	4,802	0	2,783	0
ILMN_1746465	FJX1	FJX1	11	-4,758	0	-2,225	0
ILMN_1715175	MET	MET	7	-4,688	0	-2,751	0
ILMN_1795342	MLPH	MLPH	2	4,626	0	2,108	0
ILMN_1703108	UBE2L6	UBE2L6	11	4,599	0	2,721	0
ILMN_2129572	F3	F3	1	-4,593	0	-2,65	0
ILMN_1660345	NGRN	NGRN	15	4,578	0	2,387	0
ILMN_1658053	DYNLRB1	DYNLRB1	20	4,504	0	3,005	0
ILMN_2150856	SERPINB2	SERPINB2	18	-4,471	0	-2,41	0
ILMN_1664543	IFIT3	IFIT3	10	4,47	0	2,218	0
ILMN_1766650	FOXA1	FOXA1	14	-4,469	0	-2,072	0
ILMN_1829845	HS.553301	HS.553301	13	4,408	0	3,363	0
ILMN_3231944	LOC100130516	LOC100130516		-4,399	0	-6,137	0
ILMN_1784602	CDKN1A	CDKN1A	6	4,381	0	2,092	0
ILMN_1768470	EIF4G1	EIF4G1	3	-4,297	0	-2,061	0
ILMN_2405233	FAM133B	FAM133B	7	-4,278	0	-2,054	0
ILMN_2148527	H19	H19	11	-4,25	0	-7,3	0
ILMN_1756071	MFGE8	MFGE8	15	4,125	0	3,204	0
ILMN_1739645	ANLN	ANLN	7	-4,096	0	-2,037	0
ILMN_3215206	LOC100133836	LOC100133836	5	4,001	0	2,078	0
ILMN_1673880	EFEMP1	EFEMP1	2	3,963	0	2,289	0
ILMN_2073604	EBP	EBP	X	3,916	0	2,207	0
ILMN_1777765	C12orf10	C12ORF10	12	3,909	0	2,098	0
ILMN_2239754	IFIT3	IFIT3	10	3,879	0	4,952	0
ILMN_1774077	GBP2	GBP2	1	3,856	0	3,317	0
ILMN_2279635	EIF4G2	EIF4G2	11	3,832	0	2,469	0
