# Supplementary material for: Implications of Targeted Genomic Disruption of β-Catenin in BxPC-3 Pancreatic Adenocarcinoma Cells
Source: PLoS One. 2014 Dec 23;9(12):e115496. doi: 10.1371/journal.pone.0115496 (PMC4275244; doi:10.1371/journal.pone.0115496)
Supplement: S3 Table — Gene Ontology enrichment analysis of the most differentially regulated transcripts between the wild type BxPC3 cells and the β-catenin deficient clones #4 and #111 (average). (PDF) [file pone.0115496.s004.pdf]

Supplementary Table 3.
Gene Ontology enrichment analysis of the most differentially regulated transcripts between the wild type BxPC3 cells and the β-catenin deficient clones #4 and #111 (average).
The GO analysis was performed using the top 85 most regulated transcripts from Supplementary Table 2.

Category	Term	Count	%	P-Value	Genes/transcripts	List Total	Pop Hits	Pop Total	Fold Enrichment	Bonferroni	Benjamini	FDR
GOTERM_BP_2	GO:0007155~cell adhesion	9	1,4	0,0	DCBLD2, LGALS3BP, FAT1, NEDD9, COL12A1, SCARB1, MFGE8, ZYX, MUC4	55	700	13945	3,26	0,46	0,46	5,81
GOTERM_BP_2	GO:0008037~cell recognition	3	0,5	0,0	SCARB1, MFGE8, SEMA3A	55	55	13945	13,83	0,90	0,68	19,90
GOTERM_BP_2	GO:0040008~regulation of growth	5	0,8	0,0	DCBLD2, CDKN1A, NEDD9, SEMA3A, IGFBP5	55	341	13945	3,72	0,99	0,82	39,36
GOTERM_BP_2	GO:0065008~regulation of biological quality	11	1,8	0,1	DCBLD2, UGT1A10, CDKN1A, ANXA8, PYGL, TXNDC5, F3, FOXA1, MT2A, SCARB1, SEMA3A	55	1469	13945	1,90	1,00	0,80	46,58
GOTERM_BP_2	GO:0006950~response to stress	12	1,9	0,1	DCBLD2, UGT1A10, CDKN1A, TMEM173, LGALS3BP, ANXA8, F3, WRNIP1, GAGE12I, SERPINB2, SCARB1, PTTG1	55	1685	13945	1,81	1,00	0,74	47,99
GOTERM_BP_2	GO:0009605~response to external stimulus	8	1,3	0,1	DCBLD2, UGT1A10, CDKN1A, ANXA8, F3, SERPINB2, SCARB1, SEMA3A	55	914	13945	2,22	1,00	0,72	51,63
GOTERM_BP_2	GO:0042445~hormone metabolic process	3	0,5	0,1	UGT1A10, FOXA1, SCARB1	55	106	13945	7,18	1,00	0,67	52,83
GOTERM_BP_2	GO:0022402~cell cycle process	6	1,0	0,1	EIF4G2, CDKN1A, PSMC4, NEDD9, ANLN, PTTG1, LOC652826	55	565	13945	2,69	1,00	0,64	54,72
GOTERM_BP_2	GO:0045926~negative regulation of growth	3	0,5	0,1	DCBLD2, CDKN1A, SEMA3A	55	110	13945	6,91	1,00	0,60	55,22
GOTERM_BP_2	GO:0032879~regulation of localization	6	1,0	0,1	F3, SCARB1, MFGE8, SEMA3A, IGFBP5, MYCBP2	55	610	13945	2,49	1,00	0,66	64,45
GOTERM_BP_2	GO:0044419~interspecies interaction between organisms	4	0,6	0,1	EIF4G1, SCARB1, MFGE8, ZYX	55	283	13945	3,58	1,00	0,67	68,76
