# Supplementary material for: Implications of Targeted Genomic Disruption of β-Catenin in BxPC-3 Pancreatic Adenocarcinoma Cells
Source: PLoS One. 2014 Dec 23;9(12):e115496. doi: 10.1371/journal.pone.0115496 (PMC4275244; doi:10.1371/journal.pone.0115496)
Supplement: S4 Table — Complete annotated heat map generated from the proteins identified and quantified by SILAC in at least three β-catenin deficient clones. (PDF) [file pone.0115496.s005.pdf]

Supplementary Table 4.
Complete annotated heat map generated from the proteins identified and quantified by SILAC in at least three β-catenin deficient clones.
Numberes indicate fold change , NaN: not detected.

Accession number	Gene name	Average	#4	#31	#79	#93	#111	
P09914	IFIT1	9,69	NaN	NaN	11,60	9,44	8,04	
O15020	SPTBN2	8,08	1,01	21,39	NaN	NaN	1,84	
P62805	HIST1H4A	6,63	7,89	12,26	0,56	5,39	7,04	
P29728	OAS2	5,36	NaN	NaN	4,84	4,79	6,46	
Q14512	FGFBP1	4,17	NaN	NaN	5,31	4,03	3,16	
Q96P70	IPO9	2,81	0,86	6,92	NaN	0,65	NaN	
P21589	NT5E	2,72	1,14	0,53	5,17	2,81	3,97	
P08670	VIM	2,65	4,66	0,40	2,31	4,12	1,75	
O75694	NUP155	2,45	0,80	7,29	1,12	NaN	0,59	
Q13753	LAMC2	2,26	NaN	NaN	3,42	1,80	1,55	
Q9P035	PTPLAD1	2,25	0,86	NaN	0,84	NaN	5,05	
P06396	GSN	2,25	0,66	1,91	2,64	2,06	3,96	
P42126	ECI1	2,23	1,31	NaN	NaN	3,21	2,18	
P42224	STAT1	2,20	0,98	NaN	3,64	1,98	NaN	
Q9Y2D5	AKAP2	2,20	1,58	NaN	2,03	2,66	2,53	
P55060	CSE1L	2,16	0,85	0,88	1,04	0,93	7,12	
Q03518	TAP1	2,15	NaN	0,81	NaN	2,42	3,21	
Q16778	HIST2H2BE	2,09	2,71	NaN	0,63	2,93	NaN	
P09382	LGALS1	2,09	1,45	0,91	2,66	2,87	2,56	
Q9UKM9	RALY	2,05	1,69	4,21	1,20	2,32	0,84	
Q9Y570	PPME1	2,03	4,57	0,79	NaN	0,74	NaN	
P06703	S100A6	1,79	1,10	1,06	1,57	2,04	3,20	
Q13526	PIN1	1,79	3,53	NaN	NaN	1,00	0,83	
P14923	JUP	1,74	1,59	1,46	NaN	1,17	2,73	
P29590	PML	1,72	1,43	NaN	1,60	2,13	NaN	
P02795	MT2A	1,69	NaN	0,33	2,34	2,40	NaN	
Q5QJE6	DNTTIP2	1,68	1,81	NaN	0,83	NaN	2,41	
Q96CS3	FAF2	1,68	2,81	0,88	NaN	1,35	NaN	
P18754	RCC1	1,67	0,60	2,99	1,00	3,15	0,62	
D6RD18	HNRNPAB	1,65	2,18	1,33	NaN	1,43	NaN	
A0FGR8	ESYT2	1,64	0,67	3,92	NaN	0,85	1,12	
P30740	SERPINB1	1,63	0,29	0,63	3,06	2,56	NaN	
P26885	FKBP2	1,62	NaN	0,65	NaN	3,14	1,08	
Q14108	SCARB2	1,60	NaN	1,16	NaN	1,09	2,54	
Q9BW60	ELOVL1	1,59	NaN	0,76	NaN	2,56	1,46	
O43390	HNRNPR	1,56	0,76	1,60	NaN	1,35	2,52	
P55265	ADAR	1,55	1,14	NaN	NaN	1,62	1,90	
P17900	GM2A	1,54	1,71	1,03	NaN	1,89	NaN	
P06731	CEACAM5	1,54	1,19	2,03	NaN	NaN	1,40	
Q92597	NDRG1	1,53	1,94	0,72	NaN	2,83	0,64	
Q9Y6N5	SQRDL	1,53	1,02	0,56	1,79	3,01	1,26	
Q9H9B4	SFXN1	1,53	1,96	NaN	1,59	1,03	NaN	
O00154	ACOT7	1,52	0,68	0,78	NaN	2,16	2,45	
P02545	LMNA	1,51	0,98	0,88	1,33	1,88	2,50	
P0C0S5	H2AFZ	1,50	0,78	NaN	0,84	2,88	NaN	> 1.5
P23193	TCEA1	**1,50**	1,03	2,20	NaN	1,27	NaN	
P17096	HMGA1	1,50	NaN	1,29	1,07	2,13	NaN	
P39748	FEN1	1,49	0,83	0,53	3,12	NaN	NaN	
O75367	H2AFY	1,49	2,03	NaN	1,46	1,36	1,12	
P13726	F3	1,49	0,50	0,63	2,95	1,55	1,81	
P62280	RPS11	1,48	0,76	1,38	4,03	0,69	0,54	
O00541	PES1	1,48	1,06	1,71	NaN	1,66	NaN	
Q9BRT2	MNF1	1,47	NaN	NaN	1,04	0,93	2,44	

P49959	MRE11A	1,47	1,84	1,38	NaN	1,18	NaN
P30838	ALDH3A1	1,46	1,64	0,67	0,65	1,59	2,76
P43034	PAFAH1B1	1,46	1,45	1,11	NaN	NaN	1,81
Q9BX68	HINT2	1,45	0,91	1,48	2,02	1,39	NaN
P31150	GDI1	1,44	0,79	0,94	NaN	2,16	1,88
P21796	VDAC1	1,43	1,99	1,08	1,33	1,61	1,13
P25787	PSMA2	1,41	0,87	2,30	0,94	1,24	1,70
P14866	HNRNPL	1,41	1,07	1,25	1,34	1,50	1,88
Q00688	FKBP3	1,40	0,89	1,42	1,78	1,69	1,23
O00116	AGPS	1,40	0,98	1,15	1,70	2,07	1,10
P46940	IQGAP1	1,39	0,80	0,85	1,03	2,55	1,73
Q86WV6	TMEM173	1,39	2,17	0,96	NaN	1,03	1,40
Q9Y6C9	MTCH2	1,39	0,79	NaN	1,43	1,43	1,90
Q8IY81	FTSJ3	1,39	NaN	NaN	1,73	1,35	1,08
Q16637	SMN1	1,38	2,57	0,48	NaN	1,10	NaN
P53041	PPP5C	1,38	2,65	0,93	1,08	0,87	NaN
P30405	PPIF	1,38	0,93	1,17	1,44	1,97	NaN
P30038	ALDH4A1	1,37	1,11	0,81	NaN	2,20	NaN
Q9Y277	VDAC3	1,37	1,51	1,22	1,24	1,63	1,25
Q9Y2T2	AP3M1	1,37	1,18	1,30	NaN	0,91	2,09
P58107	EPPK1	1,37	3,35	0,76	NaN	0,70	0,66
P45880	VDAC2	1,36	1,10	1,06	1,56	1,95	1,14
Q6P179	ERAP2	1,36	2,27	NaN	NaN	0,82	0,98
P07910	HNRNPC	1,35	2,36	NaN	0,88	NaN	0,82
Q9NQR4	NIT2	1,34	0,80	0,96	3,48	0,81	0,67
P28838	LAP3	1,34	1,02	0,69	2,53	1,26	1,21
Q9NSD9	FARSB	1,32	1,81	0,99	1,16	NaN	NaN
P30043	BLVRB	1,32	0,95	1,60	NaN	1,40	NaN
O43795	MYO1B	1,32	0,98	0,91	NaN	1,62	1,75
P51659	HSD17B4	1,31	2,03	1,00	1,29	1,13	1,12
P20810	CAST	1,31	1,98	NaN	0,73	NaN	1,22
Q92599	SEPT8	1,31	1,71	1,07	NaN	1,15	NaN
Q01469	FABP5	1,30	0,85	0,80	1,69	2,36	0,82
P14324	FDPS	1,30	1,38	0,85	1,10	NaN	1,88
P48147	PREP	1,30	0,71	0,96	NaN	1,94	1,60
P63151	PPP2R2A	1,30	1,12	0,92	1,86	NaN	NaN
Q53H12	AGK	1,30	1,90	1,15	NaN	0,85	NaN
P27348	YWHAQ	1,30	0,99	0,91	1,22	1,61	1,75
P12429	ANXA3	1,29	0,98	0,91	1,50	1,29	1,79
P19367	HK1	1,29	1,26	1,22	NaN	1,73	0,93
Q13838	DDX39B	1,28	NaN	1,21	NaN	0,92	1,72
P00403	MT-CO2	1,28	1,31	1,88	NaN	NaN	0,66
P21291	CSRP1	1,27	1,12	1,05	1,40	1,31	1,49
P35080	PFN2	1,27	NaN	NaN	1,18	1,49	1,13
P48735	IDH2	1,27	0,87	1,42	NaN	1,51	NaN
Q9H4L5	OSBPL3	1,26	2,11	0,73	1,72	0,66	1,10
P10301	RRAS	1,26	0,83	2,21	NaN	1,10	0,89
O14974	PPP1R12A	1,26	0,62	NaN	1,24	1,91	NaN
P17931	LGALS3	1,25	1,61	1,07	1,00	1,29	1,30
P50570	DNM2	1,25	NaN	0,85	NaN	1,02	1,87
P35237	SERPINB6	1,24	0,77	0,69	2,22	1,00	1,50
P11021	HSPA5	1,23	0,74	0,77	1,26	1,65	1,75
P53396	ACLY	1,23	0,58	0,97	1,35	NaN	2,03
O43707	ACTN4	1,23	0,73	2,04	0,82	1,62	0,95
O95571	ETHE1	1,23	1,12	1,35	NaN	0,50	1,94
Q9Y224	C14orf166	1,22	1,88	0,61	NaN	1,18	NaN
P54578	USP14	1,22	0,67	0,85	1,75	1,61	1,23
Q9Y3B3	TMED7	1,22	1,92	0,62	1,22	NaN	1,12
P12814	ACTN1	1,22	0,83	0,93	0,94	1,70	1,69

Q9Y6M1	IGF2BP2	1,22	0,92	0,84	1,33	1,78	NaN
Q08380	LGALS3BP	1,21	1,11	1,02	1,07	0,80	2,07
O43837	IDH3B	1,21	1,08	1,41	1,41	0,94	NaN
Q96KR1	ZFR	1,21	1,60	NaN	NaN	0,74	1,28
Q96PK6	RBM14	1,20	0,57	1,00	NaN	1,75	1,50
P63261	ACTG1	1,20	0,80	NaN	0,79	1,22	1,99
Q15050	RRS1	1,20	1,06	1,20	NaN	1,34	NaN
Q9Y2Z4	YARS2	1,20	0,96	1,07	1,35	1,41	NaN
Q9P0K7	RAI14	1,20	1,18	0,98	NaN	NaN	1,43
P26639	TARS	1,19	1,26	1,62	1,12	0,78	NaN
P13746	HLA-A	1,19	0,55	NaN	1,93	1,08	NaN
P50453	SERPINB9	1,19	0,83	NaN	1,62	1,11	NaN
P51858	HDGF	1,19	0,84	1,12	1,23	1,37	1,37
P45973	CBX5	1,18	1,62	1,62	NaN	0,89	0,60
P99999	CYCS	1,18	1,60	1,06	1,24	1,20	0,80
P63104	YWHAZ	1,18	0,97	0,86	1,28	1,41	1,38
P51991	HNRNPA3	1,18	1,10	1,48	1,06	1,45	0,80
Q92499	DDX1	1,17	1,09	0,90	NaN	1,16	1,55
P30044	PRDX5	1,17	1,09	0,88	1,05	1,20	1,65
P60983	GMFB	1,17	0,86	1,80	NaN	0,86	NaN
P00367	GLUD1	1,17	1,13	0,83	0,97	1,55	1,38
Q96KP4	CNDP2	1,17	0,85	0,85	2,56	0,70	0,90
Q53GQ0	HSD17B12	1,17	1,08	NaN	1,71	0,72	NaN
P43307	SSR1	1,17	1,23	1,33	NaN	0,94	NaN
O15212	PFDN6	1,17	0,82	1,67	1,01	NaN	NaN
O14745	SLC9A3R1	1,16	0,78	NaN	1,52	1,19	NaN
Q9NSE4	IARS2	1,16	0,79	1,51	1,09	1,32	1,09
Q99470	SDF2	1,16	0,79	NaN	1,42	NaN	1,27
Q14257	RCN2	1,16	1,10	1,77	NaN	0,60	NaN
P61019	RAB2A	1,16	1,05	1,28	1,28	1,19	0,98
P17655	CAPN2	1,16	NaN	0,86	1,37	0,95	1,44
Q13151	HNRNPA0	1,15	1,71	1,00	NaN	0,75	NaN
P29466	CASP1	1,15	0,91	0,68	NaN	1,87	1,15
Q9UNH7	SNX6	1,15	1,25	NaN	1,24	0,56	1,55
P36952	SERPINB5	1,15	1,03	0,78	1,02	1,51	1,40
O75832	PSMD10	1,15	1,44	1,04	0,99	1,11	NaN
P46063	RECQL	1,14	1,37	0,94	NaN	1,12	NaN
Q99436	PSMB7	1,14	0,96	1,66	NaN	0,81	NaN
O75531	BANF1	1,14	0,99	1,29	0,91	1,04	1,47
P08134	RHOC	1,14	1,01	0,89	NaN	1,28	1,37
Q9BPW9	DHRS9	1,14	1,09	1,22	NaN	NaN	1,10
P38117	ETFB	1,13	1,18	1,15	NaN	1,29	0,92
Q6NUK1	SLC25A24	1,13	1,02	0,84	1,37	1,15	1,29
P09960	LTA4H	1,13	0,86	1,21	NaN	1,43	1,03
O60763	USO1	1,13	0,77	NaN	1,58	1,29	0,88
Q9Y281	CFL2	1,13	0,74	0,60	NaN	1,47	1,69
Q86UX7	FERMT3	1,12	0,76	0,72	1,55	1,04	1,55
P55084	HADHB	1,12	1,18	1,08	NaN	1,11	NaN
P68366	TUBA4A	1,12	0,96	0,92	0,98	1,29	1,46
Q9NZB2	FAM120A	1,12	0,65	NaN	NaN	1,24	1,47
Q01081	U2AF1	1,12	1,39	1,14	NaN	0,83	NaN
O75396	SEC22B	1,12	0,93	1,06	1,34	1,21	1,05
Q99848	EBNA1BP2	1,12	1,32	1,17	NaN	1,19	0,79
P50502	ST13	1,12	0,96	0,86	1,53	NaN	NaN
P61081	UBE2M	1,12	1,02	0,93	NaN	1,58	0,93
Q01082	SPTBN1	1,11	0,86	1,09	0,84	0,93	1,85
P00558	PGK1	1,11	1,02	0,88	0,83	0,99	1,85
P31949	S100A11	1,11	0,93	1,00	0,88	0,83	1,93
Q93009	USP7	1,11	1,78	1,00	NaN	0,56	NaN

O43657	TSPAN6	1,11	1,32	1,56	NaN	0,45	NaN
P34932	HSPA4	1,11	1,55	0,73	1,02	1,00	1,25
P15559	NQO1	1,11	1,41	0,87	NaN	1,05	NaN
O43237	DYNC1LI2	1,11	1,23	1,34	NaN	0,75	NaN
Q9NR12	PDLIM7	1,11	1,54	0,74	1,44	1,01	0,80
Q9NR45	NANS	1,11	0,85	1,07	0,94	1,56	NaN
Q99426	TBCB	1,10	0,76	0,70	1,74	1,22	1,10
Q56VL3	OCIAD2	1,10	1,90	0,75	NaN	0,66	NaN
P62829	RPL23	1,10	1,62	0,80	NaN	0,88	NaN
P62937	PPIA	1,10	0,92	0,75	0,84	0,97	2,02
Q00610	CLTC	1,10	0,96	0,93	1,02	1,25	1,34
P07741	APRT	1,10	1,02	0,75	NaN	1,53	NaN
Q27J81	INF2	1,10	0,78	0,63	0,93	NaN	2,05
Q00059	TFAM	1,10	NaN	1,11	NaN	0,84	1,34
Q14192	FHL2	1,10	1,25	1,34	NaN	NaN	0,70
Q15691	MAPRE1	1,10	0,93	1,22	NaN	1,14	NaN
Q9BVK6	TMED9	1,10	1,25	1,14	0,90	NaN	NaN
P22626	HNRNPA2B1	1,10	0,93	1,12	1,12	1,43	0,88
Q7Z406	MYH14	1,10	0,58	2,72	NaN	0,40	0,68
Q9HB07	C12orf10	1,09	1,30	NaN	0,85	1,30	0,93
Q16629	SRSF7	1,09	0,86	1,35	NaN	1,07	NaN
P36957	DLST	1,09	1,12	0,81	NaN	1,35	NaN
Q15459	SF3A1	1,09	1,10	0,93	1,35	1,39	0,68
P50914	RPL14	1,09	1,28	0,93	1,21	0,94	NaN
O95831	AIFM1	1,09	0,75	1,05	NaN	NaN	1,47
O75533	SF3B1	1,09	1,08	NaN	NaN	0,87	1,31
P78406	RAE1	1,09	0,67	1,03	NaN	1,56	NaN
Q12905	ILF2	1,09	1,08	0,91	0,85	1,50	NaN
P11387	TOP1	1,09	0,90	1,22	NaN	1,47	0,75
P00505	GOT2	1,08	0,88	0,97	1,08	1,44	1,05
O95573	ACSL3	1,08	0,77	0,91	1,20	0,98	1,56
Q9H4M9	EHD1	1,08	0,86	0,94	1,14	1,13	1,35
P11216	PYGB	1,08	0,88	1,01	1,17	1,31	1,04
P31930	UQCRC1	1,08	1,16	1,20	0,98	1,47	0,59
Q8N1F7	NUP93	1,08	0,69	1,62	1,11	1,20	0,78
Q14008	CKAP5	1,08	0,78	NaN	1,21	0,79	1,54
P31040	SDHA	1,08	1,35	0,83	0,89	1,12	1,20
Q06323	PSME1	1,08	0,90	1,23	1,15	0,89	1,22
Q9NUJ1	ABHD10	1,08	1,15	1,04	1,07	1,05	NaN
Q06481	APLP2	1,08	0,97	0,78	NaN	NaN	1,48
O00151	PDLIM1	1,07	0,91	0,79	0,90	1,32	1,45
P14625	HSP90B1	1,07	0,77	0,81	1,24	1,42	1,12
P60981	DSTN	1,07	0,88	0,91	1,03	1,36	1,18
P15104	GLUL	1,07	0,66	NaN	0,85	1,70	NaN
Q9UHB6	LIMA1	1,07	1,46	0,79	0,96	NaN	NaN
Q9Y4W6	AFG3L2	1,07	1,27	1,31	NaN	1,27	0,43
P25788	PSMA3	1,07	0,81	NaN	1,24	1,00	1,22
Q99829	CPNE1	1,07	0,69	1,04	1,48	1,02	1,10
P38646	HSPA9	1,06	1,85	0,86	0,75	0,96	0,89
P13804	ETFA	1,06	1,02	1,17	0,91	1,23	0,97
O95834	EML2	1,06	0,94	0,88	1,15	1,27	NaN
P05026	ATP1B1	1,06	1,27	0,73	NaN	1,18	NaN
P05141	SLC25A5	1,06	1,15	0,88	1,06	1,39	0,82
O15554	KCNN4	1,06	0,77	1,15	NaN	0,90	1,42
P49755	TMED10	1,06	0,97	1,41	0,95	0,86	1,10
Q96HE7	ERO1L	1,06	0,61	0,83	1,53	1,16	1,16
P54136	RARS	1,06	1,33	0,77	1,10	1,10	0,98
P22307	SCP2	1,05	1,06	0,88	1,19	1,04	1,10
P19256	CD58	1,05	NaN	0,96	NaN	1,08	1,12

O43143	DHX15	1,05	1,21	0,81	NaN	1,13	NaN
P05455	SSB	1,05	0,72	1,26	NaN	1,43	0,79
Q9BS26	ERP44	1,05	0,82	1,38	1,28	0,72	NaN
O43678	NDUFA2	1,05	1,05	1,43	NaN	NaN	0,67
O14818	PSMA7	1,05	0,93	0,85	1,08	1,36	1,02
O15260	SURF4	1,05	0,71	0,69	NaN	1,08	1,71
P12081	HARS	1,05	1,73	0,49	NaN	NaN	0,92
Q9UKK9	NUDT5	1,05	0,90	0,92	NaN	1,32	NaN
Q15363	TMED2	1,05	0,82	0,95	1,25	1,13	1,08
P62258	YWHAE	1,05	0,90	0,79	1,23	1,29	1,02
Q6NZI2	PTRF	1,04	0,99	0,64	1,19	0,99	1,41
O76094	SRP72	1,04	NaN	1,29	NaN	0,90	0,94
Q02218	OGDH	1,04	1,06	0,77	NaN	1,30	NaN
Q9NZ08	ERAP1	1,04	2,03	0,60	NaN	0,55	0,99
O60610	DIAPH1	1,04	1,50	0,81	1,15	0,91	0,83
P23381	WARS	1,04	0,96	0,58	1,71	0,93	1,02
P30041	PRDX6	1,04	0,84	NaN	1,03	1,09	1,19
P55735	SEC13	1,04	0,84	1,51	0,94	0,86	NaN
Q13492	PICALM	1,04	0,99	0,86	NaN	0,89	1,41
Q13523	PRPF4B	1,04	1,00	1,17	NaN	NaN	0,94
P62873	GNB1	1,04	1,07	0,98	1,22	0,73	1,18
Q14764	MVP	1,04	0,84	1,03	1,23	1,11	0,97
P25705	ATP5A1	1,04	1,17	0,88	0,87	1,26	1,00
Q92878	RAD50	1,04	1,07	0,91	1,20	0,96	NaN
Q9P206	KIAA1522	1,03	0,53	1,21	NaN	0,73	1,67
P62820	RAB1A	1,03	0,94	0,62	1,49	1,11	1,00
O95202	LETM1	1,03	0,93	1,45	NaN	0,71	NaN
P68371	TUBB4B	1,03	0,89	1,12	1,14	0,92	1,08
P22695	UQCRC2	1,03	1,18	0,88	NaN	1,03	NaN
Q9HB71	CACYBP	1,03	0,87	0,90	1,28	1,06	1,04
P07355	ANXA2	1,03	1,03	0,63	0,90	0,89	1,69
P62316	SNRPD2	1,03	0,99	0,77	1,32	NaN	NaN
Q9BUJ2	HNRNPUL1	1,03	1,13	0,86	NaN	1,09	NaN
P07814	EPRS	1,03	0,89	0,78	1,24	1,00	1,22
Q9UMS4	PRPF19	1,03	0,98	0,67	2,11	0,80	0,57
Q99623	PHB2	1,02	1,37	1,03	0,94	NaN	0,74
Q8WVM8	SCFD1	1,02	0,90	1,05	NaN	1,28	0,85
P49411	TUFM	1,02	0,91	0,75	1,16	1,30	0,97
Q13813	SPTAN1	1,02	0,79	1,04	0,72	0,87	1,67
O94905	ERLIN2	1,02	0,79	1,71	0,78	0,79	NaN
P55809	OXCT1	1,02	0,95	1,07	1,06	0,99	NaN
O00567	NOP56	1,02	1,03	1,26	NaN	1,10	0,67
Q9BSJ8	ESYT1	1,02	0,79	0,90	NaN	0,97	1,40
Q8N3F8	MICALL1	1,01	0,74	1,37	NaN	0,64	1,31
P09874	PARP1	1,01	0,75	0,74	1,36	0,96	1,26
P10768	ESD	1,01	0,97	0,66	1,02	1,28	1,14
O94760	DDAH1	1,01	0,43	NaN	NaN	1,42	1,19
Q5K651	SAMD9	1,01	1,07	0,70	NaN	1,27	NaN
P10599	TXN	1,01	0,90	0,86	0,86	0,97	1,47
P23526	AHCY	1,01	0,77	0,93	1,45	1,00	0,91
P55786	NPEPPS	1,01	0,85	1,11	1,26	0,86	0,98
Q08945	SSRP1	1,01	0,72	0,69	0,93	1,24	1,48
P53618	COPB1	1,01	0,91	0,67	1,11	0,95	1,42
Q9BQE3	TUBA1C	1,01	1,01	1,15	NaN	0,87	NaN
Q14204	DYNC1H1	1,01	0,78	0,66	1,06	1,05	1,50
P21281	ATP6V1B2	1,01	1,03	0,82	1,36	1,19	0,64
P09622	DLD	1,01	0,69	1,00	NaN	1,35	0,99
Q92530	PSMF1	1,01	0,76	1,34	0,92	NaN	NaN
Q07157	TJP1	1,01	1,22	NaN	NaN	0,90	0,90

P52565	ARHGDIA	1,01	0,82	1,10	1,21	1,19	0,71
P04083	ANXA1	1,01	0,97	0,80	0,86	0,97	1,43
Q9Y2Z0	SUGT1	1,00	0,92	0,76	NaN	0,91	1,43
P41252	IARS	1,00	0,93	0,74	1,40	0,97	0,98
O60437	PPL	1,00	0,94	1,21	0,46	0,78	1,63
P20339	RAB5A	1,00	0,66	1,82	NaN	0,53	NaN
P53634	CTSC	1,00	1,02	1,22	NaN	0,77	NaN
Q9Y4K1	AIM1	1,00	0,70	0,94	0,93	1,44	NaN
Q14103	HNRNPD	1,00	0,55	1,38	NaN	1,03	1,05
P49327	FASN	1,00	0,72	0,65	2,78	0,31	0,55
Q14258	TRIM25	1,00	1,16	1,08	0,96	1,03	0,78
P07919	UQCRH	1,00	1,19	0,97	NaN	0,84	NaN
P16152	CBR1	1,00	0,99	1,22	0,58	1,22	0,99
P16949	STMN1	1,00	0,87	1,16	0,88	0,94	1,15
O14950	MYL12B	1,00	0,89	1,00	0,81	NaN	1,30
Q9UGI8	TES	1,00	0,69	1,01	0,98	1,02	1,29
O00515	LAD1	1,00	0,74	1,48	NaN	0,77	NaN
Q9Y4L1	HYOU1	1,00	0,69	0,88	NaN	1,42	NaN
P33176	KIF5B	0,99	1,06	0,83	1,26	0,83	NaN
Q9H4G0	EPB41L1	0,99	0,84	1,61	NaN	NaN	0,53
P46976	GYG1	0,99	1,17	1,12	0,86	NaN	0,82
P04632	CAPNS1	0,99	0,80	0,84	0,87	1,08	1,37
P06748	NPM1	0,99	1,51	0,78	0,70	0,97	1,00
P21980	TGM2	0,99	0,84	1,03	0,93	0,78	1,38
Q03252	LMNB2	0,99	0,59	1,10	1,18	1,30	0,79
O94903	PROSC	0,99	1,12	0,73	NaN	NaN	1,12
O95292	VAPB	0,99	0,95	1,09	NaN	0,93	NaN
P20618	PSMB1	0,99	0,90	1,08	0,94	0,96	1,07
O14980	XPO1	0,99	0,83	0,87	1,76	0,70	0,79
Q02809	PLOD1	0,99	0,81	1,22	0,87	1,06	NaN
Q16851	UGP2	0,99	0,98	0,68	1,11	1,03	1,14
Q9UBR2	CTSZ	0,99	0,93	1,23	NaN	0,81	0,98
O94973	AP2A2	0,99	1,28	0,79	NaN	0,89	NaN
O75643	SNRNP200	0,98	0,90	0,93	1,01	0,88	1,20
P27824	CANX	0,98	1,59	0,76	0,80	0,93	0,84
P49591	SARS	0,98	1,38	0,67	NaN	0,90	NaN
P51553	IDH3G	0,98	1,00	1,00	NaN	0,95	NaN
Q9Y4P3	TBL2	0,98	0,60	NaN	NaN	1,41	0,94
P10586	PTPRF	0,98	0,44	1,32	NaN	0,82	1,35
Q6P2E9	EDC4	0,98	1,03	1,08	NaN	1,17	0,65
P07737	PFN1	0,98	1,00	0,82	0,99	1,01	1,09
P26038	MSN	0,98	1,12	0,67	0,99	1,37	0,76
Q9BPW8	NIPSNAP1	0,98	0,73	1,11	NaN	1,10	NaN
O95232	LUC7L3	0,98	0,81	1,35	NaN	0,78	NaN
P10606	COX5B	0,98	1,60	0,84	NaN	NaN	0,50
Q92614	MYO18A	0,98	NaN	0,90	NaN	0,91	1,13
P23284	PPIB	0,98	0,78	0,85	0,77	0,84	1,66
Q9Y262	EIF3L	0,98	0,99	0,39	NaN	1,56	NaN
P36542	ATP5C1	0,98	1,27	0,93	0,81	0,97	0,91
Q9H223	EHD4	0,98	0,90	0,76	1,38	1,08	0,77
P30040	ERP29	0,98	0,72	0,89	NaN	1,57	0,73
P41250	GARS	0,98	0,71	0,63	1,46	1,11	NaN
O60506	SYNCRIP	0,98	0,79	0,80	0,91	1,04	1,34
P13987	CD59	0,98	0,95	1,22	0,79	0,99	0,93
P10155	TROVE2	0,98	1,07	0,86	NaN	1,01	0,96
P61923	COPZ1	0,97	0,88	0,73	0,93	1,05	1,28
Q1KMD3	HNRNPUL2	0,97	0,81	0,82	1,05	1,35	0,84
Q9Y3D7	PAM16	0,97	0,97	1,22	NaN	0,73	NaN
Q13148	TARDBP	0,97	0,83	1,01	NaN	1,08	NaN

Q9H0D6	XRN2	0,97	1,08	0,84	NaN	1,00	NaN
P29218	IMPA1	0,97	0,94	0,81	1,17	NaN	NaN
Q9NWV4	C1orf123	0,97	0,83	0,66	1,43	NaN	NaN
P11177	PDHB	0,97	1,04	1,09	NaN	0,81	0,95
P16615	ATP2A2	0,97	0,94	0,52	1,20	1,11	1,08
Q14116	IL18	0,97	1,00	1,19	NaN	0,81	0,88
P61224	RAP1B	0,97	0,99	0,93	NaN	0,99	NaN
Q5JWF2	GNAS	0,97	1,25	1,13	0,69	0,81	NaN
Q9P1F3	ABRACL	0,97	1,05	1,06	NaN	0,80	NaN
P09651	HNRNPA1	0,97	0,88	1,03	0,94	0,90	1,09
Q9GZS3	WDR61	0,97	0,90	0,95	0,79	0,84	1,36
P13010	XRCC5	0,97	0,88	0,75	NaN	1,34	0,90
P50402	EMD	0,97	0,96	0,87	0,83	1,21	NaN
Q9P2J5	LARS	0,97	1,50	0,82	NaN	0,88	0,67
P18031	PTPN1	0,97	0,92	0,94	1,42	0,59	NaN
Q92688	ANP32B	0,97	0,93	0,87	NaN	1,05	1,02
Q15717	ELAVL1	0,97	0,89	0,79	NaN	1,22	NaN
Q12907	LMAN2	0,97	1,14	0,90	0,86	NaN	NaN
P42166	TMPO	0,97	0,62	0,99	1,65	0,79	0,78
P62879	GNB2	0,97	0,77	0,92	NaN	1,19	0,98
P35270	SPR	0,96	0,84	1,26	0,82	0,94	NaN
P00441	SOD1	0,96	0,67	0,62	0,92	0,98	1,63
P60900	PSMA6	0,96	0,92	0,69	1,12	1,02	1,07
P12236	SLC25A6	0,96	1,36	0,75	NaN	0,78	NaN
P84095	RHOG	0,96	0,84	0,91	NaN	1,00	1,10
Q6XQN6	NAPRT1	0,96	0,62	0,70	1,26	0,98	1,25
O95881	TXNDC12	0,96	0,92	1,20	0,74	0,98	NaN
Q9UJZ1	STOML2	0,96	0,73	0,99	0,84	0,80	1,44
Q15907	RAB11B	0,96	0,99	0,90	1,02	1,07	0,82
P61011	SRP54	0,96	0,84	0,88	NaN	1,16	NaN
Q14697	GANAB	0,96	0,83	0,75	1,03	0,89	1,30
P08107	HSPA1A	0,96	0,85	0,96	0,98	1,07	0,93
P78527	PRKDC	0,96	0,93	0,85	0,89	0,88	1,24
O76003	GLRX3	0,96	0,87	0,77	0,95	1,28	0,92
P48444	ARCN1	0,96	0,80	0,91	NaN	1,04	1,08
Q92696	RABGGTA	0,96	0,66	1,12	NaN	0,96	1,09
O60825	PFKFB2	0,96	0,86	NaN	0,79	NaN	1,22
O00764	PDXK	0,96	0,73	0,79	0,96	1,24	1,06
P30101	PDIA3	0,96	0,71	0,82	0,88	1,18	1,19
P52907	CAPZA1	0,96	0,95	0,98	0,94	1,01	0,90
P47755	CAPZA2	0,95	0,58	0,78	NaN	0,99	1,47
Q00341	HDLBP	0,95	0,75	NaN	1,72	0,70	0,65
Q16181	SEPT7	0,95	1,00	0,73	NaN	1,03	1,06
P09661	SNRPA1	0,95	0,91	1,02	0,69	1,11	1,04
P43243	MATR3	0,95	1,00	0,73	1,01	0,90	1,13
P30153	PPP2R1A	0,95	1,26	0,90	0,95	0,88	0,78
Q14974	KPNB1	0,95	0,79	0,94	1,03	1,17	0,84
P51572	BCAP31	0,95	1,04	NaN	0,91	NaN	0,91
Q9ULC4	MCTS1	0,95	1,15	0,84	NaN	NaN	0,87
Q13011	ECH1	0,95	1,12	0,96	0,76	0,97	NaN
Q99714	HSD17B10	0,95	0,87	NaN	1,05	0,91	0,98
Q16666	IFI16	0,95	1,06	0,65	1,20	0,99	0,86
Q99459	CDC5L	0,95	0,95	1,30	0,86	0,96	0,69
P50238	CRIP1	0,95	0,50	1,46	NaN	0,89	NaN
P62333	PSMC6	0,95	0,86	0,61	NaN	1,38	NaN
Q15029	EFTUD2	0,95	1,12	1,01	NaN	0,72	NaN
Q86VP6	CAND1	0,95	0,81	0,94	NaN	1,10	NaN
Q13541	EIF4EBP1	0,95	0,67	0,89	NaN	1,26	0,98
Q9HC38	GLOD4	0,95	1,08	NaN	0,60	0,83	1,29

P29034	S100A2	0,95	1,20	0,78	0,95	0,88	0,94
P62318	SNRPD3	0,95	0,90	1,41	0,74	0,89	0,81
P55072	VCP	0,95	0,63	0,89	0,90	0,98	1,34
P28066	PSMA5	0,95	0,94	1,02	0,92	1,02	0,84
P40926	MDH2	0,95	0,94	0,87	0,97	1,18	0,78
Q92616	GCN1L1	0,95	0,71	0,68	1,11	0,96	1,28
P52888	THOP1	0,95	1,06	NaN	0,98	1,02	0,73
Q9BXP5	SRRT	0,95	0,87	1,41	NaN	0,84	0,67
P46459	NSF	0,95	0,76	1,16	0,92	NaN	NaN
O60664	PLIN3	0,95	0,83	1,05	0,66	0,98	1,21
P37802	TAGLN2	0,95	0,77	0,73	0,89	1,10	1,24
Q16658	FSCN1	0,95	0,84	0,73	0,99	1,22	0,95
P49792	RANBP2	0,95	1,00	1,04	1,06	0,89	0,74
P11279	LAMP1	0,95	1,08	1,02	NaN	0,70	0,98
Q15417	CNN3	0,95	0,87	0,54	1,33	NaN	1,04
O00429	DNM1L	0,94	0,87	0,62	NaN	1,02	1,27
P20042	EIF2S2	0,94	0,96	1,12	NaN	0,78	0,92
Q08211	DHX9	0,94	1,00	0,77	0,98	1,01	0,96
F8VVM2	SLC25A3	0,94	1,24	0,85	NaN	0,74	NaN
P63167	DYNLL1	0,94	0,84	NaN	1,34	NaN	0,65
P60709	ACTB	0,94	0,82	0,50	NaN	1,08	1,37
P16070	CD44	0,94	1,45	1,07	0,65	0,62	0,92
P35579	MYH9	0,94	0,85	0,73	1,06	0,98	1,09
P07384	CAPN1	0,94	0,83	0,87	0,93	1,14	0,94
P15374	UCHL3	0,94	0,81	0,91	NaN	0,98	1,06
P40121	CAPG	0,94	0,74	0,69	1,05	1,23	0,99
Q9Y3I0	C22orf28	0,94	1,01	0,80	NaN	0,96	0,99
Q9Y617	PSAT1	0,94	0,47	0,81	1,54	0,66	1,21
P54819	AK2	0,94	1,09	0,72	NaN	1,15	0,79
Q08J23	NSUN2	0,94	1,07	0,92	NaN	0,70	1,06
Q96I99	SUCLG2	0,94	1,17	1,06	NaN	0,36	1,16
P00390	GSR	0,94	0,84	NaN	NaN	1,20	0,77
P00492	HPRT1	0,94	0,96	1,10	NaN	0,75	NaN
P00568	AK1	0,94	0,90	1,20	NaN	0,71	NaN
P42285	SKIV2L2	0,94	1,17	0,82	NaN	0,82	NaN
P49589	CARS	0,94	1,16	0,82	NaN	NaN	0,83
P35606	COPB2	0,94	0,78	1,05	0,87	0,88	1,10
P27797	CALR	0,94	0,62	0,77	0,96	1,16	1,17
P31947	SFN	0,93	0,98	0,69	0,89	1,24	0,87
P05120	SERPINB2	0,93	1,06	0,79	1,40	0,28	1,14
Q9H4A4	RNPEP	0,93	1,01	0,99	NaN	0,80	NaN
P28072	PSMB6	0,93	0,91	0,93	NaN	0,96	0,93
P60228	EIF3E	0,93	0,83	0,70	NaN	0,97	1,23
P31946	YWHAB	0,93	1,07	0,95	0,97	0,85	0,82
P53621	COPA	0,93	1,04	0,72	0,97	0,87	1,06
Q15019	SEPT2	0,93	0,90	0,68	1,17	1,08	0,83
O00299	CLIC1	0,93	0,86	0,78	1,01	1,08	0,92
Q04917	YWHAH	0,93	0,78	0,81	NaN	1,20	NaN
Q13310	PABPC4	0,93	0,95	0,91	NaN	0,93	NaN
Q9P2E9	RRBP1	0,93	1,01	1,10	0,74	1,03	0,77
Q13243	SRSF5	0,93	1,18	1,43	NaN	0,55	0,56
O95373	IPO7	0,93	0,84	1,05	NaN	0,90	NaN
P13798	APEH	0,93	0,89	1,48	0,54	0,44	1,29
P52597	HNRNPF	0,93	0,89	0,91	0,69	0,77	1,38
Q07065	CKAP4	0,93	0,78	0,62	1,15	1,17	0,92
P08195	SLC3A2	0,93	0,56	NaN	0,99	1,44	0,72
Q15637	SF1	0,93	0,71	1,25	NaN	0,82	NaN
Q9BVI4	NOC4L	0,93	0,86	1,14	NaN	0,78	NaN
P23528	CFL1	0,93	0,86	0,75	0,94	1,06	1,02

Q9NY33	DPP3	0,93	1,04	0,77	0,93	1,00	0,89
P27105	STOM	0,92	1,24	0,71	0,91	0,84	NaN
P43246	MSH2	0,92	0,71	0,98	NaN	1,08	NaN
P62834	RAP1A	0,92	1,00	NaN	0,88	0,89	NaN
P06865	HEXA	0,92	1,48	1,07	NaN	0,54	0,60
P49748	ACADVL	0,92	1,08	NaN	0,84	0,64	1,13
P28482	MAPK1	0,92	0,66	1,15	NaN	0,70	1,18
P62701	RPS4X	0,92	0,94	1,04	1,00	0,71	NaN
Q12906	ILF3	0,92	0,97	0,84	0,97	0,94	0,89
P42765	ACAA2	0,92	0,95	0,75	0,97	0,90	1,04
O43396	TXNL1	0,92	1,59	0,68	NaN	0,49	NaN
O75436	VPS26A	0,92	1,07	0,95	0,97	NaN	0,69
Q15436	SEC23A	0,92	0,56	0,99	NaN	NaN	1,21
O76021	RSL1D1	0,92	1,09	0,79	NaN	1,15	0,65
Q9UBE0	SAE1	0,92	0,88	0,87	NaN	0,81	1,12
Q9UQE7	SMC3	0,92	0,82	0,85	NaN	0,77	1,24
O60925	PFDN1	0,92	1,12	0,76	0,88	NaN	NaN
O75439	PMPCB	0,92	1,23	0,89	0,64	NaN	NaN
Q05048	CSTF1	0,92	NaN	1,48	0,83	0,45	NaN
Q6P2Q9	PRPF8	0,92	1,03	0,63	NaN	1,10	NaN
P61970	NUTF2	0,92	0,89	0,45	0,80	0,94	1,52
O95456	PSMG1	0,92	0,82	0,84	1,37	0,65	NaN
Q96TA1	FAM129B	0,92	0,75	0,81	NaN	0,83	1,29
P40939	HADHA	0,92	1,06	0,81	0,86	1,06	0,81
P07858	CTSB	0,92	1,21	1,01	0,73	1,15	0,49
Q5JRX3	PITRM1	0,92	0,96	0,94	0,73	1,11	0,85
P25789	PSMA4	0,92	0,92	0,66	NaN	0,72	1,37
Q8N163	KIAA1967	0,92	1,15	0,61	NaN	0,83	1,08
Q14376	GALE	0,92	0,87	0,95	0,81	1,04	NaN
Q3MHD2	LSM12	0,92	0,77	NaN	1,18	1,09	0,63
O00743	PPP6C	0,92	1,09	NaN	0,93	0,73	NaN
P52948	NUP98	0,92	1,19	NaN	NaN	0,89	0,67
P10515	DLAT	0,92	0,73	0,78	NaN	1,24	NaN
O00505	KPNA3	0,92	1,28	0,77	NaN	0,70	NaN
P07437	TUBB	0,92	0,93	0,56	0,85	0,92	1,32
O75083	WDR1	0,92	0,84	1,07	0,71	0,95	1,01
P13489	RNH1	0,92	0,84	0,78	1,13	1,04	0,79
O14737	PDCD5	0,91	0,83	NaN	0,85	0,96	1,02
Q9UFN0	NIPSNAP3A	0,91	0,56	0,78	NaN	1,40	0,92
P22314	UBA1	0,91	0,83	0,75	0,96	0,94	1,09
P52209	PGD	0,91	0,73	1,04	1,09	0,82	0,89
P26599	PTBP1	0,91	0,93	0,78	0,82	0,89	1,15
P30085	CMPK1	0,91	0,72	0,94	NaN	NaN	1,08
Q7L2H7	EIF3M	0,91	0,75	0,96	1,03	NaN	NaN
P55327	TPD52	0,91	0,65	1,31	0,78	NaN	NaN
Q86V81	ALYREF	0,91	0,98	1,33	0,64	NaN	0,70
P50416	CPT1A	0,91	1,47	0,90	NaN	0,75	0,53
P48047	ATP5O	0,91	1,18	0,99	0,66	0,88	0,85
Q9H0U4	RAB1B	0,91	1,00	0,74	0,99	NaN	NaN
Q9H1B7	IRF2BPL	0,91	1,50	NaN	NaN	0,60	0,63
P62314	SNRPD1	0,91	0,76	1,03	0,94	NaN	NaN
Q08AF3	SLFN5	0,91	0,78	NaN	NaN	1,04	0,91
B4DLN1	SLC25A10	0,91	0,75	0,89	NaN	1,43	0,57
P62244	RPS15A	0,91	1,28	0,95	NaN	0,63	0,78
P06132	UROD	0,91	1,00	0,81	0,45	1,83	0,45
P19338	NCL	0,91	0,89	0,78	0,92	1,00	0,95
Q6ZVM7	TOM1L2	0,91	0,67	0,97	1,08	NaN	NaN
P49588	AARS	0,91	0,62	0,70	1,07	0,94	1,20
P06576	ATP5B	0,91	1,22	0,79	0,78	0,99	0,75

Q16513	PKN2	0,91	0,63	0,87	1,12	1,00	NaN
P63241	EIF5A	0,90	0,90	0,93	0,81	1,28	0,60
P62899	RPL31	0,90	1,08	1,01	NaN	0,62	NaN
Q8TAT6	NPLOC4	0,90	0,77	0,82	NaN	1,12	NaN
Q6UX04	CWC27	0,90	0,90	0,79	0,85	1,07	NaN
P16435	POR	0,90	0,75	1,13	0,92	0,81	NaN
O14579	COPE	0,90	1,09	0,59	NaN	0,89	1,04
P40925	MDH1	0,90	1,19	0,52	NaN	1,06	0,84
P09211	GSTP1	0,90	0,92	0,78	0,72	1,12	0,97
Q06830	PRDX1	0,90	0,79	0,85	0,90	1,12	0,85
P62826	RAN	0,90	0,99	0,78	1,02	0,95	0,77
P53999	SUB1	0,90	1,09	0,76	NaN	0,85	NaN
Q16401	PSMD5	0,90	0,84	0,69	NaN	1,00	1,07
P40227	CCT6A	0,90	0,83	0,78	0,93	0,62	1,34
P62942	FKBP1A	0,90	0,78	0,80	0,90	1,07	0,95
Q15365	PCBP1	0,90	0,96	0,70	1,10	0,87	0,87
P35658	NUP214	0,90	0,37	0,91	NaN	1,42	NaN
Q9UBQ7	GRHPR	0,90	0,53	1,46	NaN	0,71	NaN
P51571	SSR4	0,90	0,92	0,91	1,09	NaN	0,67
P12830	CDH1	0,90	0,86	1,92	0,45	0,36	NaN
Q14847	LASP1	0,90	0,95	1,05	NaN	0,76	0,83
Q7L014	DDX46	0,90	1,16	0,77	NaN	1,05	0,61
Q9HCC0	MCCC2	0,90	1,33	NaN	NaN	0,53	0,83
Q9UBL3	ASH2L	0,90	0,57	0,56	1,56	NaN	NaN
Q07955	SRSF1	0,90	0,95	1,33	0,65	0,81	0,74
Q00839	HNRNPU	0,90	0,88	0,75	0,92	1,06	0,87
Q9NZM1	MYOF	0,90	1,15	0,75	0,94	0,83	0,81
P15121	AKR1B1	0,89	0,73	1,08	0,81	0,90	0,95
P37837	TALDO1	0,89	0,82	0,94	0,87	0,95	0,89
Q13155	AIMP2	0,89	0,78	0,70	1,55	0,87	0,57
P17858	PFKL	0,89	0,85	0,94	NaN	0,89	NaN
P18859	ATP5J	0,89	1,11	NaN	0,65	NaN	0,92
P37198	NUP62	0,89	0,80	NaN	NaN	0,87	1,01
P62495	ETF1	0,89	1,24	0,83	NaN	0,61	NaN
Q13907	IDI1	0,89	0,60	NaN	NaN	0,77	1,31
Q14019	COTL1	0,89	0,84	0,98	NaN	0,87	0,88
Q9Y5Y2	NUBP2	0,89	1,05	0,66	1,28	0,58	NaN
O75347	TBCA	0,89	1,17	0,68	0,96	0,78	0,87
Q969H8	C19orf10	0,89	0,70	0,92	0,98	1,08	0,77
P23434	GCSH	0,89	0,86	1,27	NaN	0,54	NaN
Q29RF7	PDS5A	0,89	1,26	NaN	0,60	NaN	0,81
Q9BRA2	TXNDC17	0,89	1,21	1,02	NaN	0,82	0,51
P60903	S100A10	0,89	0,89	0,82	NaN	0,80	1,05
Q15149	PLEC	0,89	1,14	0,87	0,80	0,99	0,64
Q9UQ80	PA2G4	0,89	0,91	0,83	1,02	1,10	0,58
P30520	ADSS	0,89	0,77	0,96	0,86	0,70	1,15
Q15181	PPA1	0,89	0,63	0,85	0,91	1,16	NaN
Q86UE4	MTDH	0,89	1,22	0,37	NaN	1,07	NaN
P15927	RPA2	0,89	1,08	NaN	0,46	NaN	1,12
O43264	ZW10	0,89	1,13	1,10	NaN	0,43	NaN
Q9Y314	NOSIP	0,89	1,21	NaN	0,74	0,71	NaN
Q6PKG0	LARP1	0,89	1,26	0,65	NaN	0,75	NaN
P30084	ECHS1	0,89	1,02	0,90	0,73	0,92	0,86
Q9H299	SH3BGRL3	0,89	1,01	0,83	0,80	1,10	0,69
P21964	COMT	0,89	1,14	0,41	1,38	0,76	0,74
Q07666	KHDRBS1	0,89	0,97	0,77	0,89	1,05	0,75
P48739	PITPNB	0,89	0,91	0,84	NaN	1,00	0,79
P50897	PPT1	0,88	0,76	1,10	NaN	0,84	0,84
Q9NRX4	PHPT1	0,88	1,06	0,84	0,70	0,78	1,04

P43304	GPD2	0,88	0,79	0,65	NaN	NaN	1,21
Q8IXI1	RHOT2	0,88	0,95	0,84	NaN	0,86	NaN
Q9UNL2	SSR3	0,88	1,04	0,80	NaN	0,81	NaN
Q92544	TM9SF4	0,88	NaN	0,83	NaN	1,05	0,77
O14979	HNRPDL	0,88	0,67	1,70	0,58	NaN	0,58
P54105	CLNS1A	0,88	1,09	1,23	NaN	0,52	0,69
P08758	ANXA5	0,88	1,10	0,81	0,66	0,89	0,95
P26641	EEF1G	0,88	0,85	0,75	0,94	1,00	0,87
O96008	TOMM40	0,88	0,82	0,75	NaN	1,07	NaN
O75947	ATP5H	0,88	1,22	0,81	0,62	0,87	NaN
P55795	HNRNPH2	0,88	0,86	0,85	NaN	0,96	0,85
P84103	SRSF3	0,88	0,95	0,77	0,79	1,08	0,81
O15347	HMGB3	0,88	1,06	1,23	0,62	0,93	0,56
P07339	CTSD	0,88	1,15	1,25	0,67	0,71	0,62
Q14165	MLEC	0,88	0,97	NaN	NaN	0,83	0,84
Q8NFU3	TSTD1	0,88	0,67	NaN	NaN	0,59	1,38
Q96FW1	OTUB1	0,88	0,78	1,16	0,87	0,97	0,62
Q9NQG5	RPRD1B	0,88	0,70	0,91	0,84	1,03	0,91
P23743	DGKA	0,88	1,15	0,95	0,89	0,75	0,65
P62979	RPS27A	0,88	0,90	1,04	0,83	0,82	0,80
Q14134	TRIM29	0,88	0,68	0,99	0,86	0,98	NaN
Q9Y5B9	SUPT16H	0,88	0,72	0,84	NaN	1,07	0,88
P35613	BSG	0,88	0,91	0,75	0,96	0,89	NaN
Q15067	ACOX1	0,88	0,65	NaN	NaN	0,86	1,12
Q5JTH9	RRP12	0,88	0,59	NaN	1,14	NaN	0,90
Q99798	ACO2	0,88	1,20	1,01	0,67	0,81	0,69
Q9UNZ2	NSFL1C	0,88	0,87	0,63	1,01	1,07	0,80
P62753	RPS6	0,88	1,16	0,91	NaN	0,68	0,75
Q15046	KARS	0,88	0,95	0,86	0,87	0,82	NaN
Q12797	ASPH	0,87	0,80	0,81	0,92	0,86	0,98
Q9Y446	PKP3	0,87	0,76	0,85	0,66	1,02	1,08
Q15185	PTGES3	0,87	0,90	0,98	0,92	0,79	0,78
O95782	AP2A1	0,87	0,85	1,04	NaN	0,73	NaN
Q01581	HMGCS1	0,87	1,36	0,63	NaN	0,63	NaN
Q15005	SPCS2	0,87	0,73	NaN	NaN	1,04	0,85
O75369	FLNB	0,87	0,92	0,74	0,99	0,84	NaN
Q9UHD8	SEPT9	0,87	1,10	0,79	NaN	0,84	0,76
P06733	ENO1	0,87	0,99	0,69	0,72	1,01	0,95
Q96C19	EFHD2	0,87	0,85	1,01	0,79	0,96	0,75
P17980	PSMC3	0,87	0,82	0,75	1,05	0,81	0,93
P20700	LMNB1	0,87	1,22	0,95	0,72	0,73	0,73
O43169	CYB5B	0,87	0,83	1,35	NaN	0,60	0,70
P09972	ALDOC	0,87	0,67	0,84	NaN	1,22	0,75
P27694	RPA1	0,87	0,85	1,52	0,50	0,87	0,61
Q01844	EWSR1	0,87	0,99	1,11	NaN	0,60	0,78
Q5VV41	ARHGEF16	0,87	0,64	0,99	NaN	0,98	NaN
Q9BT78	COPS4	0,87	1,00	0,81	NaN	0,80	NaN
Q9HAW8	UGT1A10	0,87	1,05	1,47	NaN	0,09	NaN
Q9UK76	HN1	0,87	0,94	1,06	0,72	1,00	0,63
O00303	EIF3F	0,87	1,06	0,73	NaN	0,82	NaN
Q99613	EIF3C	0,87	0,89	1,15	0,95	0,77	0,59
Q92896	GLG1	0,87	0,88	1,23	0,60	0,82	0,81
P31948	STIP1	0,87	0,84	0,95	0,79	0,78	0,98
Q9H0E2	TOLLIP	0,87	0,86	NaN	0,64	0,58	1,39
P33240	CSTF2	0,87	0,89	0,98	NaN	0,73	NaN
Q14498	RBM39	0,87	0,74	0,77	1,09	NaN	NaN
O00592	PODXL	0,87	1,23	1,02	NaN	0,35	NaN
P09525	ANXA4	0,87	0,82	1,02	0,74	0,83	0,92
Q7Z2W4	ZC3HAV1	0,87	0,71	0,77	1,25	0,99	0,61

A0AVT1	UBA6	0,86	NaN	0,53	0,95	0,94	1,04
Q5T4S7	UBR4	0,86	0,92	0,79	1,01	0,84	0,76
P63173	RPL38	0,86	0,91	0,96	NaN	0,72	NaN
P05198	EIF2S1	0,86	0,95	0,90	NaN	0,74	NaN
P12955	PEPD	0,86	0,99	0,79	NaN	0,97	0,70
Q92841	DDX17	0,86	0,89	1,00	0,82	1,00	0,60
P22102	GART	0,86	0,74	0,67	0,94	0,72	1,23
P35241	RDX	0,86	1,32	0,68	NaN	0,58	NaN
P13667	PDIA4	0,86	0,41	0,80	0,98	1,35	0,76
P14314	PRKCSH	0,86	0,94	0,87	0,77	0,85	0,87
Q6YN16	HSDL2	0,86	0,83	0,93	NaN	0,82	NaN
Q9NVA2	SEPT11	0,86	0,83	NaN	NaN	0,86	0,89
P61981	YWHAG	0,86	0,90	0,48	NaN	1,26	0,80
O00571	DDX3X	0,86	1,12	0,71	NaN	0,56	1,05
P84090	ERH	0,86	0,84	1,05	NaN	0,80	0,75
P11766	ADH5	0,86	0,84	1,00	0,82	0,90	0,73
Q09028	RBBP4	0,86	0,88	0,84	NaN	1,07	0,64
Q9HAV7	GRPEL1	0,86	0,87	NaN	NaN	0,96	0,74
Q9Y285	FARSA	0,86	0,70	NaN	NaN	1,12	0,75
P17812	CTPS1	0,86	0,66	0,73	0,87	0,93	1,09
P35659	DEK	0,86	0,81	1,07	NaN	0,66	0,88
Q07020	RPL18	0,85	1,23	0,77	0,78	0,64	NaN
P17174	GOT1	0,85	0,95	0,79	0,87	0,69	0,97
Q00796	SORD	0,85	0,47	0,75	0,95	1,11	0,99
P46734	MAP2K3	0,85	0,80	0,99	NaN	0,77	NaN
Q9P289	MST4	0,85	0,83	0,63	NaN	1,10	NaN
Q15056	EIF4H	0,85	1,05	0,78	NaN	0,73	NaN
P22061	PCMT1	0,85	0,76	0,69	0,84	1,12	NaN
O15144	ARPC2	0,85	0,95	1,55	0,49	0,42	NaN
O43852	CALU	0,85	0,56	0,73	1,10	1,15	0,72
P04406	GAPDH	0,85	0,99	0,83	0,88	0,62	0,94
Q6P4A8	PLBD1	0,85	1,08	0,60	NaN	NaN	0,87
P14174	MIF	0,85	0,97	0,92	0,87	0,86	0,63
P30048	PRDX3	0,85	0,85	0,86	0,63	1,11	0,80
O94979	SEC31A	0,85	1,10	0,64	NaN	1,07	0,59
Q96QK1	VPS35	0,85	0,77	1,33	0,62	0,68	NaN
P82673	MRPS35	0,85	0,86	0,85	NaN	0,84	NaN
Q13177	PAK2	0,85	0,63	NaN	1,11	0,81	NaN
Q9Y266	NUDC	0,85	0,84	0,69	1,19	0,82	0,71
O15269	SPTLC1	0,85	0,70	1,00	1,16	0,52	0,87
P56537	EIF6	0,85	0,90	0,71	1,11	0,84	0,69
Q15434	RBMS2	0,85	0,84	0,57	NaN	1,14	NaN
P0CW22	RPS17L	0,85	0,74	0,73	1,40	0,86	0,51
Q13347	EIF3I	0,85	0,95	0,75	0,86	1,00	0,68
P22087	FBL	0,85	0,85	NaN	0,77	1,14	0,63
P07954	FH	0,85	0,80	0,83	NaN	0,91	NaN
P54725	RAD23A	0,85	0,90	0,84	NaN	0,80	NaN
P54727	RAD23B	0,85	0,83	1,02	0,69	NaN	NaN
Q99439	CNN2	0,85	1,00	0,93	0,55	0,68	1,07
P48637	GSS	0,85	0,75	0,93	0,96	0,79	0,80
P07237	P4HB	0,85	0,75	0,75	0,82	1,06	0,85
P60866	RPS20	0,84	0,73	1,12	0,88	0,65	NaN
Q9BWD1	ACAT2	0,84	0,64	0,79	NaN	0,70	1,25
P28074	PSMB5	0,84	0,69	0,84	NaN	0,82	1,03
Q14166	TTLL12	0,84	0,75	0,84	0,96	0,83	NaN
P67936	TPM4	0,84	0,73	0,99	0,90	0,74	0,86
P06744	GPI	0,84	0,89	0,90	0,66	0,81	0,96
Q8IZP0	ABI1	0,84	0,89	0,85	0,79	NaN	NaN
Q8N4X5	AFAP1L2	0,84	0,75	1,07	0,71	NaN	NaN

O14744	PRMT5	0,84	0,77	0,81	NaN	0,95	NaN
Q9Y371	SH3GLB1	0,84	0,81	0,76	NaN	NaN	0,96
Q92973	TNPO1	0,84	0,97	0,88	NaN	0,86	0,66
P55263	ADK	0,84	0,95	0,93	0,85	0,64	NaN
Q04760	GLO1	0,84	1,29	0,85	0,74	0,63	0,70
P34896	SHMT1	0,84	NaN	NaN	0,72	1,07	0,73
P54577	YARS	0,84	0,79	0,86	0,72	1,14	0,69
Q13263	TRIM28	0,84	0,86	1,08	0,77	0,69	0,80
P00338	LDHA	0,84	0,87	0,67	0,91	1,06	0,69
P35232	PHB	0,84	0,98	0,76	0,98	0,81	0,66
P62158	CALM1	0,84	0,77	0,79	0,83	0,81	0,99
Q13185	CBX3	0,84	0,87	0,75	0,87	0,86	NaN
Q9UHB9	SRP68	0,84	1,05	0,94	0,56	0,80	NaN
Q4VC31	CCDC58	0,84	0,86	0,86	NaN	0,79	NaN
Q9H8Y8	GORASP2	0,84	1,26	0,46	NaN	0,79	NaN
Q14683	SMC1A	0,84	1,04	0,80	NaN	0,67	NaN
Q32Q12	NME1-NME2	0,84	0,77	0,86	NaN	0,88	NaN
Q8N1G4	LRRC47	0,84	0,70	NaN	1,11	0,70	NaN
Q15084	PDIA6	0,84	0,68	0,68	0,96	1,12	0,74
Q13162	PRDX4	0,84	0,57	0,90	1,26	0,74	0,71
P11310	ACADM	0,83	0,99	0,83	NaN	0,62	0,90
Q9Y2A7	NCKAP1	0,83	0,95	0,78	NaN	0,69	0,92
Q13200	PSMD2	0,83	1,22	0,73	0,87	0,62	0,73
P61604	HSPE1	0,83	0,95	0,97	0,77	0,80	0,68
P25786	PSMA1	0,83	0,86	0,59	0,96	0,87	0,89
Q9BUL8	PDCD10	0,83	0,99	0,91	NaN	0,60	NaN
P51665	PSMD7	0,83	0,96	0,84	NaN	0,70	NaN
P35573	AGL	0,83	1,10	0,70	NaN	0,80	0,73
P83731	RPL24	0,83	0,91	1,12	NaN	0,67	0,63
Q9UM54	MYO6	0,83	0,56	1,20	NaN	0,41	1,16
P11142	HSPA8	0,83	0,96	0,73	0,78	0,83	0,86
Q96AG4	LRRC59	0,83	1,08	0,73	NaN	0,68	NaN
Q9UL25	RAB21	0,83	0,85	NaN	NaN	0,43	1,21
O95372	LYPLA2	0,83	1,02	1,08	NaN	0,61	0,61
Q99471	PFDN5	0,83	0,82	0,77	1,19	0,54	NaN
P04080	CSTB	0,83	0,81	1,10	0,67	0,75	0,82
P07108	DBI	0,83	0,96	1,20	0,63	0,57	0,79
P12956	XRCC6	0,83	0,93	0,80	0,78	0,91	0,73
E9PAV3	NACA	0,83	0,97	0,95	0,57	NaN	NaN
O94832	MYO1D	0,83	NaN	0,90	NaN	0,67	0,92
P61353	RPL27	0,83	1,04	0,93	0,63	0,72	NaN
Q13247	SRSF6	0,83	1,10	0,83	0,77	NaN	0,62
P04075	ALDOA	0,83	1,04	0,79	0,76	0,84	0,72
P08559	PDHA1	0,83	1,07	0,80	0,86	0,60	0,81
P04844	RPN2	0,83	0,91	0,76	0,79	0,71	0,97
P13796	LCP1	0,83	0,85	0,67	0,76	0,94	0,92
P43490	NAMPT	0,83	0,66	0,84	0,98	0,73	0,93
P39023	RPL3	0,83	0,84	0,86	1,02	0,85	0,57
P23246	SFPQ	0,83	0,96	0,76	0,70	1,04	0,68
P38606	ATP6V1A	0,83	0,82	0,94	NaN	0,64	0,91
P45974	USP5	0,83	0,87	0,69	NaN	0,63	1,12
Q15392	DHCR24	0,83	NaN	1,40	NaN	0,35	0,73
P31937	HIBADH	0,83	0,55	1,04	NaN	0,89	NaN
Q86X55	CARM1	0,83	0,62	NaN	NaN	1,17	0,69
P52272	HNRNPM	0,83	0,98	0,93	0,67	0,94	0,61
Q15369	TCEB1	0,83	1,12	0,80	0,72	0,61	0,88
P13807	GYS1	0,83	0,91	0,79	0,90	NaN	0,70
Q9UJS0	SLC25A13	0,82	0,53	0,99	NaN	0,82	0,96
P31939	ATIC	0,82	0,80	0,75	0,98	0,79	0,80

P10809	HSPD1	0,82	0,81	0,85	0,72	1,04	0,70
P04843	RPN1	0,82	1,00	0,80	0,78	0,77	0,77
Q7KZF4	SND1	0,82	0,59	0,76	0,90	1,05	0,82
P12694	BCKDHA	0,82	NaN	1,17	0,66	0,64	NaN
P09234	SNRPC	0,82	0,53	1,10	0,87	NaN	0,79
P29508	SERPINB3	0,82	0,96	1,31	NaN	0,57	0,45
Q15758	SLC1A5	0,82	0,39	1,26	0,73	1,19	0,54
Q86UP2	KTN1	0,82	0,84	0,79	0,94	0,86	0,68
P35998	PSMC2	0,82	0,79	0,87	0,78	0,63	1,04
P55036	PSMD4	0,82	0,55	1,12	0,79	NaN	NaN
Q12800	TFCP2	0,82	0,93	NaN	0,83	0,79	0,73
O75934	BCAS2	0,82	1,00	NaN	NaN	0,76	0,70
P62241	RPS8	0,82	0,91	0,88	0,82	0,86	0,62
Q14980	NUMA1	0,82	0,71	0,79	0,81	0,87	0,91
P00491	PNP	0,82	0,97	0,76	0,78	0,89	0,69
P22234	PAICS	0,82	0,78	0,71	1,01	0,83	0,76
Q15293	RCN1	0,82	0,88	0,85	NaN	0,80	0,74
P50395	GDI2	0,82	0,96	0,70	NaN	0,85	0,76
Q15437	SEC23B	0,82	0,99	0,79	NaN	0,67	NaN
Q01813	PFKP	0,82	1,14	0,57	NaN	0,74	NaN
Q9BQL6	FERMT1	0,82	0,75	0,88	NaN	0,82	NaN
Q9BZZ5	API5	0,82	0,99	0,76	NaN	0,70	NaN
P04899	GNAI2	0,82	1,09	0,91	0,66	0,80	0,62
P35268	RPL22	0,81	0,85	0,83	0,82	0,76	NaN
Q96HC4	PDLIM5	0,81	0,80	0,77	0,86	0,82	0,82
P25685	DNAJB1	0,81	NaN	1,01	NaN	0,69	0,74
P58546	MTPN	0,81	0,61	0,83	NaN	1,00	NaN
P63000	RAC1	0,81	0,83	0,72	NaN	0,89	NaN
P11717	IGF2R	0,81	1,31	0,97	NaN	0,16	NaN
Q9H2U2	PPA2	0,81	0,78	0,78	NaN	0,88	NaN
P50552	VASP	0,81	0,97	0,93	NaN	0,76	0,59
Q01130	SRSF2	0,81	0,90	0,97	0,71	0,67	NaN
Q9P2I0	CPSF2	0,81	1,17	0,77	NaN	0,77	0,54
O00170	AIP	0,81	1,00	0,71	0,72	NaN	NaN
P11413	G6PD	0,81	0,88	1,01	0,61	0,64	0,91
P11940	PABPC1	0,81	1,05	0,74	0,91	NaN	0,54
P61106	RAB14	0,81	0,77	0,92	NaN	0,76	0,79
Q9C0C2	TNKS1BP1	0,81	0,75	1,20	0,78	0,69	0,63
P60174	TPI1	0,81	0,96	0,76	0,69	0,76	0,88
Q15233	NONO	0,81	0,94	0,71	0,95	0,70	0,75
Q9Y2B0	CNPY2	0,81	0,78	0,59	0,62	1,36	0,70
Q9BRX8	FAM213A	0,81	0,75	NaN	NaN	0,84	0,84
P17987	TCP1	0,81	0,88	0,81	0,87	0,71	0,77
P21926	CD9	0,81	0,65	0,89	0,72	0,64	1,14
O75131	CPNE3	0,81	0,76	0,89	NaN	0,98	0,60
P55884	EIF3B	0,81	0,97	0,64	0,76	0,86	NaN
P17844	DDX5	0,81	1,05	0,58	NaN	0,49	1,11
Q10713	PMPCA	0,81	0,81	0,95	NaN	0,66	NaN
Q15223	PVRL1	0,81	1,14	0,72	NaN	0,56	NaN
Q9P2B2	PTGFRN	0,81	NaN	0,84	NaN	1,03	0,55
P46777	RPL5	0,81	0,91	0,96	0,83	0,68	0,65
P12532	CKMT1A	0,81	0,91	1,08	NaN	0,64	0,59
P13073	COX4I1	0,80	1,24	1,02	NaN	0,48	0,48
P18206	VCL	0,80	0,75	0,71	0,69	0,78	1,09
Q8N6H7	ARFGAP2	0,80	0,80	0,99	NaN	NaN	0,62
Q9UNF0	PACSIN2	0,80	NaN	0,96	0,78	0,67	NaN
P29373	CRABP2	0,80	0,38	0,82	0,89	0,72	1,20
P23396	RPS3	0,80	0,93	0,93	0,75	0,65	0,75
P62081	RPS7	0,80	0,77	0,71	0,80	0,78	0,95

O00232	PSMD12	0,80	1,20	0,50	NaN	0,59	0,91
P18124	RPL7	0,80	0,95	0,96	0,80	0,73	0,56
P62424	RPL7A	0,80	0,89	1,22	0,71	0,68	0,50
P30050	RPL12	0,80	0,76	0,93	NaN	0,71	NaN
P18669	PGAM1	0,80	0,81	0,80	0,77	1,05	0,57
P61289	PSME3	0,80	0,86	NaN	0,92	0,61	NaN
O43583	DENR	0,80	1,00	0,53	1,16	0,63	0,66
O75223	GGCT	0,80	0,86	0,77	0,88	0,67	NaN
P62917	RPL8	0,80	0,76	0,71	NaN	0,61	1,10
P49023	PXN	0,79	0,65	0,79	NaN	0,68	1,06
O95994	AGR2	0,79	0,74	1,59	0,39	0,32	0,93
P23229	ITGA6	0,79	0,89	0,81	0,63	0,92	0,72
Q99832	CCT7	0,79	0,86	0,82	0,87	0,67	0,75
Q07021	C1QBP	0,79	0,81	1,02	0,71	0,99	0,44
Q92598	HSPH1	0,79	0,96	0,69	0,84	0,82	0,66
Q9NVI7	ATAD3A	0,79	0,81	0,72	NaN	NaN	0,85
O60701	UGDH	0,79	0,58	0,80	0,97	0,82	NaN
P68104	EEF1A1	0,79	0,71	NaN	0,94	0,83	0,69
Q9NTK5	OLA1	0,79	0,86	0,78	NaN	0,75	0,78
Q9P258	RCC2	0,79	0,85	0,98	0,92	0,67	0,54
P54920	NAPA	0,79	1,00	0,78	0,89	0,50	0,79
Q9Y678	COPG1	0,79	0,87	0,77	0,80	0,90	0,62
Q93052	LPP	0,79	0,85	0,84	NaN	0,68	NaN
Q9UHX1	PUF60	0,79	0,84	NaN	0,74	0,79	NaN
P34897	SHMT2	0,79	0,86	0,96	0,63	0,85	0,65
Q13131	PRKAA1	0,79	0,53	0,87	NaN	0,88	0,88
P61160	ACTR2	0,79	0,92	1,07	0,49	0,91	0,55
P30086	PEBP1	0,79	0,72	0,71	0,83	0,86	0,82
P07195	LDHB	0,79	0,93	0,84	0,78	0,59	0,80
Q6Y7W6	GIGYF2	0,79	0,93	NaN	0,72	0,97	0,53
Q9UKY7	CDV3	0,79	0,70	1,12	0,76	0,57	NaN
P40429	RPL13A	0,79	0,89	0,81	NaN	0,66	NaN
P15880	RPS2	0,79	0,87	0,73	0,87	0,80	0,66
P29144	TPP2	0,79	0,63	0,66	0,72	1,26	0,66
Q9Y3F4	STRAP	0,79	0,76	0,60	1,32	0,57	0,68
Q92817	EVPL	0,79	0,84	1,23	0,61	0,61	0,64
P39656	DDOST	0,78	0,89	0,76	NaN	0,78	0,71
P63244	GNB2L1	0,78	0,87	0,73	0,82	0,91	0,59
B4DDR3		0,78	0,84	NaN	0,79	NaN	0,72
O95757	HSPA4L	0,78	0,67	0,85	NaN	0,83	NaN
P61513	RPL37A	0,78	0,94	0,98	0,60	0,61	NaN
Q9NZL4	HSPBP1	0,78	0,86	NaN	0,86	0,83	0,58
O15371	EIF3D	0,78	0,75	0,60	1,11	0,91	0,54
P60842	EIF4A1	0,78	0,95	0,72	0,98	0,74	0,52
P31943	HNRNPH1	0,78	1,01	0,98	0,60	0,84	0,48
Q04837	SSBP1	0,78	0,67	0,77	0,77	0,94	0,76
P14618	PKM	0,78	1,06	0,63	0,71	0,69	0,82
P54709	ATP1B3	0,78	0,61	1,26	NaN	0,97	0,28
P84098	RPL19	0,78	1,01	1,08	NaN	0,55	0,48
P17301	ITGA2	0,78	0,93	0,78	0,81	0,60	NaN
P61978	HNRNPK	0,78	0,87	0,82	0,76	0,67	NaN
P25398	RPS12	0,78	0,87	0,81	0,68	0,78	0,76
P29692	EEF1D	0,78	0,79	0,76	0,77	0,88	0,70
Q99497	PARK7	0,78	0,93	0,75	0,60	0,89	0,72
P46778	RPL21	0,78	0,93	0,69	0,77	0,72	NaN
Q9P0V9	SEPT10	0,78	0,79	0,83	NaN	0,73	0,76
O95359	TACC2	0,78	0,70	0,89	NaN	0,74	NaN
P50991	CCT4	0,78	0,85	0,62	0,85	0,80	0,76
O43399	TPD52L2	0,78	0,65	0,74	0,78	0,98	0,73

P62750	RPL23A	0,77	0,87	0,93	0,82	0,76	0,49
P16144	ITGB4	0,77	0,87	0,76	0,70	0,95	0,59
Q92945	KHSRP	0,77	0,78	1,03	0,75	0,73	0,58
Q96CW1	AP2M1	0,77	0,88	0,55	1,17	0,71	0,56
O00560	SDCBP	0,77	0,80	0,76	NaN	0,76	NaN
P07203	GPX1	0,77	0,82	0,99	NaN	0,51	NaN
Q12996	CSTF3	0,77	NaN	0,71	NaN	0,66	0,95
P61313	RPL15	0,77	1,19	0,77	NaN	0,57	0,56
Q9HDC9	APMAP	0,77	1,00	0,72	NaN	0,87	0,50
Q9Y4Y9	LSM5	0,77	0,77	0,49	0,75	1,08	NaN
P21333	FLNA	0,77	0,79	0,64	0,88	0,75	0,80
Q13283	G3BP1	0,77	1,22	0,63	0,63	0,54	0,84
P61247	RPS3A	0,77	0,84	0,82	0,69	0,73	0,78
O60443	DFNA5	0,77	0,80	0,63	NaN	0,88	NaN
P30626	SRI	0,77	0,56	0,88	NaN	0,87	NaN
Q9NR31	SAR1A	0,77	0,67	0,65	NaN	0,99	NaN
P13797	PLS3	0,77	0,86	0,69	0,71	0,82	0,77
P32119	PRDX2	0,77	0,85	0,97	0,73	0,47	0,82
P26373	RPL13	0,77	0,82	0,72	0,99	0,72	0,59
P46783	RPS10	0,77	0,91	0,91	0,65	0,63	0,74
P21399	ACO1	0,77	0,44	1,09	0,77	NaN	NaN
P49368	CCT3	0,77	0,80	0,79	0,70	0,74	0,80
P60660	MYL6	0,77	0,78	0,68	0,85	0,64	0,88
Q01518	CAP1	0,77	1,14	0,68	0,75	0,71	0,55
O00159	MYO1C	0,76	1,00	0,52	0,82	0,68	0,80
P46782	RPS5	0,76	0,83	0,77	0,55	0,78	0,88
P49915	GMPS	0,76	0,92	0,75	NaN	0,64	0,73
P31153	MAT2A	0,76	1,54	0,80	0,59	0,44	0,43
P62913	RPL11	0,76	0,92	0,83	0,82	0,73	0,49
P07900	HSP90AA1	0,76	0,84	0,73	0,81	0,77	0,64
Q14152	EIF3A	0,76	0,86	0,62	NaN	0,79	NaN
Q9NQC3	RTN4	0,76	0,47	NaN	0,89	0,91	NaN
Q99460	PSMD1	0,76	0,99	0,65	NaN	0,63	NaN
Q9Y230	RUVBL2	0,76	0,69	0,80	NaN	0,78	NaN
P63220	RPS21	0,76	0,85	0,98	0,69	0,72	0,54
O43278	SPINT1	0,75	0,99	1,18	0,45	NaN	0,40
P78344	EIF4G2	0,75	0,74	0,74	0,78	NaN	NaN
Q16531	DDB1	0,75	0,81	0,69	0,73	0,78	NaN
P13639	EEF2	0,75	0,80	0,71	0,81	0,69	0,75
Q02878	RPL6	0,75	1,07	0,76	NaN	0,42	NaN
Q9UJU6	DBNL	0,75	0,90	1,20	0,62	0,56	0,47
O75874	IDH1	0,75	0,64	1,08	NaN	0,53	NaN
P46013	MKI67	0,75	1,04	NaN	NaN	0,22	0,99
Q9H2G2	SLK	0,75	0,63	NaN	NaN	0,98	0,63
P18621	RPL17	0,75	0,78	0,93	NaN	0,53	NaN
P67870	CSNK2B	0,75	0,78	NaN	0,70	0,76	NaN
Q99733	NAP1L4	0,75	0,64	0,68	0,87	0,56	0,98
Q3KQU3	MAP7D1	0,75	0,69	0,88	0,84	0,78	0,54
O00425	IGF2BP3	0,75	0,57	0,87	NaN	0,88	0,66
P56192	MARS	0,75	1,02	0,66	NaN	0,73	0,57
O60547	GMDS	0,74	1,01	1,05	0,51	NaN	0,41
P52566	ARHGDIB	0,74	0,89	0,66	0,66	0,63	0,88
O75937	DNAJC8	0,74	0,59	0,73	NaN	0,91	NaN
O14773	TPP1	0,74	0,83	0,99	NaN	NaN	0,41
Q9NP92	MRPS30	0,74	1,01	0,53	NaN	0,69	NaN
Q9BR76	CORO1B	0,74	0,82	0,40	0,88	0,72	0,89
P62888	RPL30	0,74	0,78	0,97	0,77	0,66	0,53
P62263	RPS14	0,74	0,96	1,01	0,90	0,46	0,38
P05023	ATP1A1	0,74	1,02	0,73	0,61	0,69	0,65

Q96I24	FUBP3	0,74	0,87	0,66	0,65	0,84	0,68
P20674	COX5A	0,74	1,05	0,67	NaN	0,50	NaN
Q16719	KYNU	0,74	0,50	0,71	NaN	1,01	NaN
Q12931	TRAP1	0,74	0,94	0,77	NaN	0,48	0,77
Q13501	SQSTM1	0,74	1,25	0,66	0,57	0,66	0,55
P61158	ACTR3	0,74	0,91	1,35	0,45	0,48	0,50
P61586	RHOA	0,74	0,86	0,93	0,59	0,69	0,62
P26368	U2AF2	0,74	0,84	0,65	0,78	0,73	0,69
P23588	EIF4B	0,74	0,63	1,07	NaN	0,65	0,60
O00231	PSMD11	0,74	0,96	NaN	0,76	0,61	0,62
O43823	AKAP8	0,74	1,09	0,60	0,52	NaN	NaN
P09429	HMGB1	0,74	0,81	0,99	0,61	0,70	0,57
P48643	CCT5	0,74	0,85	0,63	0,70	0,75	0,75
O95433	AHSA1	0,74	0,69	0,53	0,90	0,81	0,75
P42566	EPS15	0,73	0,62	0,79	NaN	0,70	0,83
Q9UQB8	BAIAP2	0,73	0,65	0,49	0,93	NaN	0,87
Q01105	SET	0,73	0,81	0,94	0,69	0,58	0,65
Q9GZP8	IMUP	0,73	0,70	0,58	NaN	0,92	NaN
Q9UKS6	PACSIN3	0,73	0,84	NaN	NaN	0,71	0,65
Q13435	SF3B2	0,73	0,78	1,08	NaN	0,80	0,27
O94826	TOMM70A	0,73	0,99	0,84	0,71	0,67	0,45
O75607	NPM3	0,73	0,73	NaN	NaN	0,47	0,99
Q8TBC4	UBA3	0,73	0,81	0,69	NaN	0,69	NaN
P36578	RPL4	0,73	0,89	0,78	0,68	0,75	0,55
Q02790	FKBP4	0,73	0,71	0,83	0,90	0,67	0,54
P78371	CCT2	0,73	0,86	0,81	0,65	0,69	0,63
Q8N3U4	STAG2	0,73	0,58	0,98	NaN	0,72	0,63
O00487	PSMD14	0,73	0,99	0,69	NaN	0,64	0,59
P15170	GSPT1	0,73	1,05	0,87	NaN	0,64	0,35
Q9Y3U8	RPL36	0,73	NaN	1,07	NaN	0,52	0,59
P18077	RPL35A	0,72	0,83	0,84	NaN	0,73	0,50
Q03135	CAV1	0,72	0,53	0,51	NaN	0,73	1,13
Q6NZY4	ZCCHC8	0,72	0,59	1,03	0,67	NaN	0,61
Q02543	RPL18A	0,72	0,91	0,68	0,88	0,66	0,49
P14550	AKR1A1	0,72	0,66	0,68	0,77	0,82	0,69
P50990	CCT8	0,72	0,89	0,73	0,88	0,58	0,54
Q9Y265	RUVBL1	0,72	0,74	0,80	0,63	0,81	0,64
P39687	ANP32A	0,72	0,82	0,85	0,50	NaN	NaN
Q8WXF1	PSPC1	0,72	0,48	0,64	0,89	0,88	NaN
Q9Y3A5	SBDS	0,72	0,86	0,72	NaN	0,58	NaN
P47895	ALDH1A3	0,72	0,72	0,73	0,90	0,60	0,65
P39019	RPS19	0,72	0,81	0,83	0,65	0,61	0,70
Q14247	CTTN	0,72	0,84	0,80	0,68	0,59	0,69
P41567	EIF1	0,72	0,90	0,82	NaN	0,44	NaN
Q6FI81	CIAPIN1	0,72	0,58	1,13	NaN	NaN	0,45
P10644	PRKAR1A	0,72	0,69	0,58	0,65	NaN	0,96
O43175	PHGDH	0,72	0,29	0,55	0,82	0,85	1,08
P62328	TMSB4X	0,72	0,69	0,82	NaN	0,61	0,75
P40763	STAT3	0,72	0,61	0,67	0,87	NaN	NaN
P52701	MSH6	0,72	0,62	NaN	NaN	0,54	0,99
Q5TFE4	NT5DC1	0,72	0,60	0,71	NaN	0,84	NaN
P09758	TACSTD2	0,72	0,63	1,00	0,53	0,51	0,91
P12270	TPR	0,72	0,76	0,83	0,69	0,74	0,56
P11172	UMPS	0,71	0,94	0,77	NaN	0,39	0,76
P62249	RPS16	0,71	0,82	0,76	0,75	0,69	0,55
P14854	COX6B1	0,71	1,29	0,83	0,43	0,54	0,48
Q9HB21	PLEKHA1	0,71	1,29	NaN	NaN	0,56	0,29
Q16822	PCK2	0,71	0,37	NaN	0,70	0,79	0,99
Q9NZN4	EHD2	0,71	0,74	0,61	0,54	0,70	0,97

Q16543	CDC37	0,71	0,68	0,78	0,74	0,86	0,49	
P23921	RRM1	0,71	1,13	0,78	NaN	0,22	NaN	
Q92882	OSTF1	0,71	0,63	0,87	0,54	0,76	0,74	
P62861	FAU	0,71	0,88	NaN	0,67	0,63	0,65	
Q13085	ACACA	0,71	0,87	0,94	NaN	0,31	NaN	
Q9NP79	VTA1	0,71	0,78	0,75	NaN	0,59	NaN	
P53582	METAP1	0,71	0,67	0,90	0,55	0,86	0,55	
O15305	PMM2	0,70	0,69	0,95	NaN	0,48	0,70	
P24534	EEF1B2	0,70	0,85	0,59	0,68	0,65	0,75	
Q8NBF2	NHLRC2	0,70	0,80	0,54	NaN	0,77	NaN	
O60493	SNX3	0,70	0,69	1,01	NaN	0,41	NaN	
Q96P48	ARAP1	0,70	0,76	0,88	NaN	0,47	NaN	
Q08257	CRYZ	0,70	0,82	0,25	NaN	1,04	NaN	
Q9Y696	CLIC4	0,70	0,90	0,69	0,57	0,65	NaN	
Q04637	EIF4G1	0,70	0,59	0,88	0,79	0,54	NaN	
P32969	RPL9	0,70	0,89	0,68	0,74	0,67	0,52	
P41091	EIF2S3	0,70	NaN	0,69	NaN	0,77	0,64	
Q96AE4	FUBP1	0,70	0,94	NaN	0,45	0,71	NaN	
Q8IVF2	AHNAK2	0,70	0,40	1,05	0,86	0,58	0,60	
P62906	RPL10A	0,70	1,05	0,77	0,43	0,74	0,50	
Q15393	SF3B3	0,70	0,86	0,75	0,56	0,62	NaN	
Q96EY1	DNAJA3	0,70	0,98	NaN	0,63	NaN	0,48	
Q9H3U1	UNC45A	0,70	0,78	NaN	NaN	0,84	0,47	
P42330	AKR1C3	0,69	1,13	0,98	0,31	0,64	0,41	
P06454	PTMA	0,69	0,84	1,06	0,48	0,59	0,50	
Q70J99	UNC13D	0,69	0,71	0,66	NaN	NaN	0,71	
O43776	NARS	0,69	0,72	0,79	NaN	0,62	0,64	
Q15785	TOMM34	0,69	0,79	NaN	0,88	0,48	0,62	
P62857	RPS28	0,69	0,86	0,94	0,56	0,55	0,55	
O95336	PGLS	0,69	0,68	0,61	0,60	0,76	0,80	
O95817	BAG3	0,69	0,86	0,51	NaN	0,70	NaN	
P55209	NAP1L1	0,69	0,66	0,74	0,70	0,57	0,78	
P62847	RPS24	0,69	0,76	0,43	0,61	NaN	0,96	
P61960	UFM1	0,69	0,59	0,69	0,45	1,03	NaN	
Q15654	TRIP6	0,69	0,57	0,81	0,87	0,66	0,53	
P63208	SKP1	0,69	0,95	NaN	0,64	0,50	0,66	
Q9BY32	ITPA	0,69	0,92	0,36	NaN	0,78	NaN	
Q92520	FAM3C	0,69	0,60	0,68	NaN	0,74	0,72	
P50995	ANXA11	0,68	0,79	0,90	0,56	0,45	0,71	
O14617	AP3D1	0,68	0,66	0,59	NaN	0,79	NaN	
P43686	PSMC4	0,68	0,79	0,61	0,75	0,67	0,58	
Q9H444	CHMP4B	**0,68**	0,71	NaN	NaN	0,60	0,73	
Q9UBT2	UBA2	0,68	0,64	0,86	0,57	0,75	0,58	
P08865	RPSA	0,68	0,76	NaN	0,59	0,79	0,58	
O95299	NDUFA10	0,68	1,11	0,84	0,53	0,29	0,63	
P35637	FUS	0,68	0,83	NaN	0,32	0,89	NaN	
P36871	PGM1	0,68	0,75	0,69	0,50	0,68	0,77	
Q8IZ83	ALDH16A1	0,68	0,66	0,82	NaN	0,55	NaN	
Q9NYL9	TMOD3	0,68	0,61	0,82	NaN	0,60	NaN	
P08581	MET	0,68	0,78	0,69	NaN	0,56	NaN	
Q9Y4E8	USP15	0,67	0,92	0,37	0,76	0,65	NaN	
O00629	KPNA4	0,67	0,66	0,76	NaN	0,60	NaN	
Q99615	DNAJC7	0,67	1,01	0,68	0,47	0,53	NaN	
P05387	RPLP2	0,67	0,78	0,83	0,56	0,65	0,54	
P61221	ABCE1	0,67	0,60	0,58	NaN	0,66	0,84	
Q09161	NCBP1	0,67	0,71	0,70	0,65	0,62	NaN	**< 0.67**
Q53EL6	PDCD4	0,67	0,72	0,73	0,51	0,95	0,43	
P05388	RPLP0	0,67	0,78	0,73	0,62	0,73	0,48	
O00410	IPO5	0,67	0,76	0,66	NaN	0,53	0,72	

P20290	BTF3	0,67	1,20	0,64	0,42	0,41	NaN
P47897	QARS	0,67	0,89	0,76	NaN	0,39	0,63
P27708	CAD	0,67	0,43	0,91	NaN	0,66	NaN
Q9NQP4	PFDN4	0,67	0,59	0,81	NaN	NaN	0,60
O15143	ARPC1B	0,67	0,98	0,80	0,60	0,39	0,56
Q07960	ARHGAP1	0,67	0,77	0,71	0,63	0,77	0,45
P08238	HSP90AB1	0,66	0,66	0,68	0,75	0,70	0,53
P78347	GTF2I	0,66	0,79	0,82	NaN	0,38	NaN
P35249	RFC4	0,66	0,59	NaN	0,65	NaN	0,75
P15311	EZR	0,66	0,84	0,73	0,65	0,52	0,57
P46937	YAP1	0,66	0,86	0,47	0,55	NaN	0,76
O60264	SMARCA5	0,66	0,74	0,78	NaN	0,46	NaN
Q8NBS9	TXNDC5	0,66	0,65	0,73	0,42	0,45	1,05
P55010	EIF5	0,66	0,67	NaN	0,70	NaN	0,60
P62854	RPS26	0,66	0,63	0,79	NaN	0,55	NaN
Q16186	ADRM1	0,66	1,00	0,47	NaN	0,50	NaN
O14497	ARID1A	0,66	NaN	NaN	0,64	0,64	0,69
Q15404	RSU1	0,66	NaN	0,82	NaN	0,64	0,51
Q96G03	PGM2	0,66	0,65	0,79	NaN	0,53	NaN
P13693	TPT1	0,66	0,79	0,93	0,38	0,62	0,56
P29401	TKT	0,66	0,82	0,89	0,43	0,50	0,64
P11586	MTHFD1	0,65	0,72	0,73	0,61	0,58	0,63
Q5T5P2	KIAA1217	0,65	1,10	0,44	NaN	0,42	NaN
P09668	CTSH	0,65	1,07	1,17	NaN	0,27	0,10
Q14789	GOLGB1	0,65	0,48	0,65	NaN	0,81	0,67
Q99622	C12orf57	0,65	0,89	0,71	NaN	0,58	0,43
P15924	DSP	0,65	0,78	0,71	0,54	0,63	0,58
P14735	IDE	0,65	0,69	0,71	NaN	0,54	NaN
P62195	PSMC5	0,65	0,93	0,69	0,36	0,60	NaN
O75534	CSDE1	0,64	0,88	0,78	NaN	0,33	0,59
Q14444	CAPRIN1	0,64	1,05	0,75	NaN	0,27	0,51
P35221	CTNNA1	0,64	0,75	0,66	0,55	0,71	0,55
P50895	BCAM	0,64	0,41	1,18	0,34	NaN	NaN
Q13405	MRPL49	0,64	NaN	0,88	0,67	NaN	0,37
Q9Y490	TLN1	0,64	0,69	0,70	0,57	0,64	0,60
P16401	HIST1H1B	0,64	0,76	NaN	NaN	0,78	0,38
P26583	HMGB2	0,64	0,73	1,19	0,31	0,32	NaN
P62633	CNBP	0,64	NaN	1,06	0,46	0,39	NaN
Q9H0A0	NAT10	0,63	0,63	0,76	NaN	0,78	0,36
P46060	RANGAP1	0,63	0,64	0,82	0,47	0,64	0,59
O60218	AKR1B10	0,63	0,67	1,17	NaN	0,29	0,39
Q9H6S3	EPS8L2	0,63	0,66	0,83	0,44	0,58	NaN
Q86W92	PPFIBP1	0,63	0,63	0,73	0,52	NaN	NaN
Q8WUM4	PDCD6IP	0,63	0,75	0,78	NaN	0,35	NaN
P98179	RBM3	0,62	1,12	1,27	NaN	0,07	0,04
P63313	TMSB10	0,62	0,90	0,87	NaN	0,39	0,34
P33991	MCM4	0,62	0,71	0,75	NaN	0,41	NaN
Q9UPN3	MACF1	0,62	0,78	0,33	NaN	NaN	0,76
P50454	SERPINH1	0,62	0,80	0,65	0,60	0,56	0,49
P05556	ITGB1	0,62	0,80	0,74	0,48	0,44	0,63
P04792	HSPB1	0,62	0,52	1,14	0,33	0,51	0,59
Q9NR30	DDX21	0,62	0,69	0,64	NaN	0,56	0,58
P25205	MCM3	0,62	0,92	0,62	NaN	0,31	NaN
Q8TD16	BICD2	0,62	0,83	0,68	NaN	0,34	NaN
P51149	RAB7A	0,61	0,76	0,68	0,59	0,55	0,49
P60953	CDC42	0,61	0,72	0,51	NaN	0,58	0,64
O60711	LPXN	0,61	1,16	0,44	0,51	0,34	NaN
A5YKK6	CNOT1	0,61	0,73	0,82	NaN	0,28	NaN
P42704	LRPPRC	0,61	0,78	0,65	0,48	0,49	0,63

P33993	MCM7	0,60	0,86	0,69	NaN	0,25	NaN
P49736	MCM2	0,60	0,90	0,62	NaN	0,28	NaN
Q09666	AHNAK	0,60	0,70	0,73	0,45	0,55	0,56
P05386	RPLP1	0,60	0,61	0,72	0,44	0,62	NaN
Q99873	PRMT1	0,60	0,93	0,78	NaN	0,27	0,41
O60716	CTNND1	0,60	0,73	NaN	0,52	0,54	NaN
P12268	IMPDH2	0,60	0,52	0,55	0,65	0,28	0,98
Q9Y624	F11R	0,59	0,74	NaN	0,49	0,54	NaN
P49321	NASP	0,59	0,77	0,72	0,44	0,55	0,47
P27635	RPL10	0,59	0,66	0,63	0,64	NaN	0,42
Q9UQ35	SRRM2	0,58	0,79	NaN	NaN	0,37	0,59
P02786	TFRC	0,58	0,86	0,74	NaN	0,14	NaN
Q9Y6E2	BZW2	0,57	0,49	0,60	NaN	0,63	NaN
Q8NC51	SERBP1	0,57	0,68	0,72	NaN	0,38	0,51
Q14126	DSG2	0,57	0,96	NaN	0,47	0,24	0,61
P00533	EGFR	0,57	0,74	0,46	NaN	0,57	0,50
P26006	ITGA3	0,57	0,67	1,04	0,36	0,22	0,54
Q7Z6Z7	HUWE1	0,56	0,82	0,38	NaN	0,48	NaN
Q00534	CDK6	0,56	0,34	0,61	NaN	0,72	NaN
P61916	NPC2	0,56	0,62	0,65	0,40	NaN	NaN
Q13084	MRPL28	0,55	NaN	0,83	NaN	0,36	0,47
O15160	POLR1C	0,55	0,48	0,58	0,84	0,42	0,44
Q13740	ALCAM	0,55	0,63	0,61	0,47	0,48	0,54
P04181	OAT	0,55	0,67	0,75	0,29	0,58	0,44
Q15847	ADIRF	0,54	0,81	0,52	NaN	0,32	0,53
Q6YHK3	CD109	0,54	1,07	1,13	0,07	0,13	0,31
Q643R3	LPCAT4	0,53	0,45	0,42	NaN	0,73	NaN
P12004	PCNA	0,53	0,90	0,67	0,42	0,27	0,38
Q9NRV9	HEBP1	0,53	0,46	0,67	NaN	0,45	NaN
P06756	ITGAV	0,52	0,60	0,71	NaN	0,26	NaN
Q9UPT8	ZC3H4	0,50	0,28	0,60	0,38	NaN	0,75
P52895	AKR1C2	0,48	0,70	NaN	0,38	NaN	0,37
P09110	ACAA1	0,48	0,38	0,84	0,37	0,33	NaN
P07942	LAMB1	0,47	0,76	NaN	0,52	0,13	NaN
Q9ULW8	PADI3	0,46	0,59	0,28	0,13	0,60	0,72
Q13835	PKP1	0,45	NaN	0,56	NaN	0,41	0,39
Q9H910	HN1L	0,45	0,69	0,52	0,27	NaN	0,32
