# Supplementary material for: Implications of Targeted Genomic Disruption of β-Catenin in BxPC-3 Pancreatic Adenocarcinoma Cells
Source: PLoS One. 2014 Dec 23;9(12):e115496. doi: 10.1371/journal.pone.0115496 (PMC4275244; doi:10.1371/journal.pone.0115496)
Supplement: S1 Materials and Methods — Quantitative proteonomics by SILAC. (DOCX) [file pone.0115496.s007.docx]

**Supplementary Materials and Methods**

**Quantitative proteonomics by SILAC**

For proteomic nanoLC-MS analysis, BxPC-3 wild type and *CTNNB1* gene disrupted clones were cultured in RPMI 1640 SILAC media (deficient of both L-arginine and L-lysine, Thermo Fisher Scientific, MA, USA) supplemented with 10 % dialyzed FBS (Thermo Fisher Scientific), 120 U/mL penicillin streptomycin (Invitrogen, Carlsbad, USA). For labeling, 500 ml Heavy RPMI medium was prepared by adding 50 mg each of ^13^C_6_^15^N_4_ L-Arginine-HCl and ^13^C_6_ L Lysine-2HCl (Thermo Fisher Scientific) while in the Light RPMI media L-Lysine-2HCl and L-Argininine-HCl was added. The *CTNNB1* gene disrupted clones were cultured in Heavy media, whereas the wild type cells were cultured in the Light media. At about 80 % confluency, the cells were harvested, washed in phosphate buffered saline and counted. The cells were lysed in 200 µL buffer containing 0.4 % (w/v) sodium-dodecyl sulfate (SDS, Sigma-Aldrich), 10 mM Tris-HCl pH 7.6 and 10 mM dithiothretiol (DTT, Sigma-Aldrich). Briefly, the cell pellets were re-suspended in lysis buffer, heated for 15 minutes at 70^0^ C and sonicated for 1 minute. Debris was removed by centrifugation at 13 000 rpm for 10 minutes at 20⁰C (Thermo Fisher Scientific). The protein concentrations were determined using Bradford Quick Assay (Bio-Rad). The samples were then combined in a 1:1 ratio (protein concentration) of wild type and *CTNNB1* gene disrupted cells. In each replicate 100 µg protein was digested for liquid chromatography-tandem mass spectrometry (LC-MS/MS) analysis using the filter-aided sample preparation (FASP) method [1] with LysC/Trypsin-mix (Promega). The samples were desalted using 100 mg C18 resins (Agilent Technologies, CA, USA) and evaporated to dryness. The samples were reconstituted in 2% acetonitrile (ACN, VWR, Radnor, PA, USA)/0.1 % trifluoroacetic c acid (TFA, Sigma-Aldrich). About 1 μg were loaded onto the LC-MS/MS system with the human proteome as background.

Each replicate were analyzed on a QExactive mass spectrometer (Thermo Fisher Scientific) in combination with a 2D-LC-system connected to a Easy nLC1000 pump (Thermo Fisher Scientific). The samples (1 μL) were loaded with a flow-rate of 0.5 μL/min for 2 minutes on a 50 μm x 4 cm butyl-methacrylate (BuMa) monolithic precolumn. The flow was subsequently split down to 40 nL/min and the peptides separated on a 10 μm x 10 m polystyrene-divinylbenzene porous layer open tubular column (PS-DVB PLOT) using gradient elution from 4-40 % ACN /0.1 % formic acid (FA, Sigma-Aldrich) in 450 minutes and up to 95 % ACN/0.1 %FA in 20 minutes. (For further information regarding column production and system setup see [2]). The eluent were ionized by electrospray ionization (ESI) using 5 μm silica-tips (New Objective, Woburn, MA, USA) with an applied voltage of 1.3 kV. The interface temperature was set to 250°C. The ions were selected for MS/MS analysis using a top 12 data-dependent method with full MS scan range from 350 – 1850 mass to charge (m/z), resolution at 70.000, AGC target value of 5e6 and maximum inject time of 256 ms. The MS/MS resolution was set to 35.000 with AGC value of 5e5 and maximum inject time of 128 ms. The isolation window was 3.0 m/z, the number of microscans 1 and the collision energy was normalized to 25. Dynamic exclusion and peptide match were on, and charge states 1 and above 7 were not selected for fragmentation.

The raw-files were processed with Proteome Discoverer (v.1.4) from Thermo using the Sequest and Mascot algorithm against the human proteome database from UniProt/SwissProt (The UniProt Consortium
**Activities at the Universal Protein Resource (UniProt))**. Methionine oxidation, N-terminal acetylation, +6 labeling of lysines and +10 labeling of arginine were set as dynamic modifications. Carbiodomethylation of cysteines were set as static modification and a false discovery rate of 0.01 was applied. Minimum peptide confidence was set to medium and minimum 2 peptides per protein were required for identification. For quantification, the average and standard deviation based three replicates were calculated using propagation of uncertainty. Each average was normalized against the mean protein abundance and a student-t test was used to calculate the p-values. Perseus (www.maxquant.org) and excel was used to generate the heat-maps. Gene Onthology enrichment analysis was done by DAVID [3] using the GOTERM_BP_2 annotation.

**References**

1. Wisniewski JR, Zougman A, Nagaraj N, Mann M (2009) Universal sample preparation method for proteome analysis. Nat Methods 6: 359-362.

2. Rogeberg M, Vehus T, Grutle L, Greibrokk T, Wilson SR, Lundanes E (2013) Separation optimization of long porous-layer open-tubular columns for nano-LC-MS of limited proteomic samples. J Sep Sci 36: 2838-2847.

3. Huang dW, Sherman BT, Lempicki RA (2009) Systematic and integrative analysis of large gene lists using DAVID bioinformatics resources. Nat Protoc 4: 44-5
